# Linear Response Functions of Densities and Spin Densities for Systematic Modeling of the QM/MM Approach for Mono- and Poly-Nuclear Transition Metal Systems

**DOI:** 10.3390/molecules24040821

**Published:** 2019-02-25

**Authors:** Colin K. Kitakawa, Tomohiro Maruyama, Jinta Oonari, Yuki Mitsuta, Takashi Kawakami, Mitsutaka Okumura, Kizashi Yamaguchi, Shusuke Yamanaka

**Affiliations:** 1 Graduate School of Science, Osaka University, Osaka 565-0871, Japan; kitakawac15@chem.sci.osaka-u.ac.jp (C.K.K.); maruyamat16@chem.sci.osaka-u.ac.jp (T.M.); oonarij16@chem.sci.osaka-u.ac.jp (J.O.); mitsutay13@chem.sci.osaka-u.ac.jp (Y.M.); kawakami@chem.sci.osaka-u.ac.jp (T.K.); ok@chem.sci.osaka-u.ac.jp (M.O.); 2 Institute for Nanodesign, Osaka University, Osaka 560-8531, Japan; yama@chem.sci.osaka-u.ac.jp; 3 Quantum information, Quantum Biology division, Institute for Open and Transdisciplinary Research Initiatives, Osaka University, Osaka 560-8531, Japan

**Keywords:** linear response function, QM/MM, transition metal complexes, enzymes

## Abstract

We applied our analysis, based on a linear response function of density and spin density, to two typical transition metal complex systems-the reaction centers of P450, and oxygen evolving center in Photosystem II, both of which contain open-shell transition metal ions. We discuss the relationship between LRF of electron density and spin density and the types of units and interactions of the systems. The computational results are discussed in relation to quantum mechanics (QM) cluster and quantum mechanics/molecular mechanics (QM/MM) modeling that are employed to compute the reaction centers of enzymes.

## 1. Introduction

The quantum mechanics/molecular mechanics (QM/MM) approach, which was first proposed in the 1970s [1,2], is now widely applied to biochemical reactions and molecular materials [3,4,5,6]. The essential idea of this approach is that the entire system can be divided into the QM region, that is described by quantum mechanics and the MM region, that is described by using classical point charges and force fields. The region of primary interest is the QM region, where QM events, such as chemical reactions and electronic excitation processes occur. The MM region is, however, also important, because the surrounding environment, such as proteins, lipids, and solvents often strongly affect the electronic structure of the QM region. For this reason, in most contemporary QM/MM methods, the electronic structure of the QM region is calculated in the presence of the MM point charges. This “electric embedding” type of QM/MM treatment usually leads to an improved modeling of the target system as desired, but sometimes it leads to over-polarization of the QM region near the QM/MM boundaries [4], resulting in less accuracy. To begin with, the validity of the QM description critically depends on the appropriate choices of important residues, lipids, and water molecules that are included in the QM region [5]. Then it is the most important step of the QM/MM calculation to determine how we model the target system. Some of the QM/MM researchers carefully examined the convergence of their QM/MM computational results with the size of the QM region [6,7,8,9,10,11,12,13,14,15]. These types of careful approaches for the QM/MM modeling is not new: Karplus and his coworkers analyzed the contributions of residues to the reaction pathway of Triosephosphate isomerase in their study in 1991 [6]. In this type of approach, the researchers examined several models, in which the residues included in the QM regions differed for each model [6]. This charge (atom) deletion analysis (CDA) method was originally used to find residues that are important for the catalytic activity of the reaction centers [6], but it is also used to examine the accuracy of the energies of QM/MM models [7,8,9]. As an extended version of CDA, Ryde and his coworkers performed a very comprehensive analysis of the effects of QM and QM/MM modeling on the proton transfer in [Ni, Fe] hydrogenase [8]. An alternative version is the charge shift analysis (CSA) proposed by Kulik and her coworkers [9]. Instead of focusing on the energy differences among the models, they estimated the difference of the charge of non-core residues between the full enzyme model and the model in which the active site residues have been removed, as follows:

(1) $$\Delta q_{Res}=q_{Res}^{holo}-q_{Res}^{apo}.$$

Here, we use the notation holo, and apo, respectively for the former and the latter models according to their papers [9,10]. The validity of the CSA model is based on their following observations [9]: The residues that exhibited a charge redistribution when the core active site substrates were removed could be used to construct an appropriate QM region, which is consistent with the sufficiently accurate QM region that reaches at the asymptotically converged QM region. They further proposed the Fukui shift analysis (FSA) [10]: In FSA, they estimated the condensed Fukui function (CFF) [16,17] of the core active site, and then they added surrounding QM residues back, one at a time and examined the CFF values of the core site. If the change of the CFF values of the core site due to the addition of the surrounding residue is large, the residue is determined to be included in the QM region. The heart of the FSA approaches is that the CFF values are used to judge whether the peripheral residues affect the electronic structure of the reaction center.

We also proposed an approach to determine the QM/MM boundary [18,19,20,21]. Our approach shares the same goal, i.e., to determine the QM/MM boundary, but it is a simpler method than the CSA or the FSA, and is based on the linear response function (LRF) [22,23,24] defined by,

(2) $$\frac{\delta \rho \left(r\right)}{\delta v\left(r\prime \right)}.$$

In our method [18,19,20,21], $\delta v\left(r^{\prime }\right)$ is a virtual perturbation, which is applied to the point $r^{\prime }$ (a point in the peripheral regions surrounding the active site), and δρ(r), which is the density change at the QM region, due to the perturbation. If δρ(r)/δv(r′) is large enough, the replacement of the peripheral regions by MM point charges is inappropriate. Since δρ(r) includes the effects of electron inflow and outflow from the peripheral regions to the active site, Equation (2) incorporates the effects covered by the FSA described above. At first glance, the relationship between the CSA and our LRF analysis seems to be inside-out. In fact, from the viewpoint of the response theory, a deletion of the active site residues is equivalent to a perturbation that has occurred at the active site, and Equation (1), $\Delta q_{Res}=q_{Res}^{holo}-q_{Res}^{apo}$, is the density change at peripheral residues, which can be expressed by the following equation:

(3) $$\Delta q_{Res}≅\frac{\delta \rho \left(peripheral\;residues\right)}{\delta v\left(active\;site\;residues\right)}≅\frac{\delta \rho \left(r\prime \right)}{\delta v\left(r\right)}.$$

However, since the linear response function is symmetric with respect to r and r′, i.e.,

(4) $$\frac{\delta \rho \left(r\prime \right)}{\delta v\left(r\right)}=\frac{\delta \rho \left(r\right)}{\delta v\left(r\prime \right)},$$

the CSA can be considered as a special version of the LRF analysis. We previously applied our LRF analysis to the fundamental covalent bonding systems, π conjugated systems, and polypeptide systems [18,19,20]. Our approach relies on the fact that if we consider δρ(r)/δv(r′) as a propagation of the effect from the perturbation δv at r′ to the response, δρ, at r, the decay of propagation will vary depending on the types of the mediated chemical bonds, such as a covalent-bond, π conjugation, hyper-conjugation, hydrogen-bonding, d-π interactions, and through-space interactions (the examples will be shown in the results section). The reason we use the “density” changes is that density is the fundamental property in the context of the density functional theory (DFT), which determines all other electronic properties of molecular systems [25,26,27].

In this study, we extended our LRF analysis to spin-polarized systems. As is well known, the transition metal complexes, containing open-shell transition metal ions, play important roles in the catalytic activity of enzymes, and electric and magnetic properties of the molecular materials [28,29,30]. For such cases, the most stable solutions for the Kohn-Sham DFT equation [26] became spin-symmetry broken solutions, where an additional property, such as spin-density [31], on-top pair density [32], and radical density [33], is needed to identify the electronic structures, because the radical character [34] of transition metal complexes is deeply related to the functionality of the active center of such enzymes and molecular materials. These various properties describe the radical character and are related to each other via known relations [35]. The spin-density is another fundamental variable of spin-unrestricted KS-DFT, and we employed the spin-density for the LRF analysis of transition metal complexes described below. We applied our LRF analysis of both the density and the spin-density to the reaction center of P450, which is a mono-nuclear Fe(III) porphyrin complex, and the reaction center of photosystem II, Mn_4_O_5_Ca complex. The results are discussed in relation to the QM cluster and QM/MM modeling that has been implemented so far, in particular from the viewpoint of chemical-bonding and intermolecular interactions.

## 2. Theoretical Background

### 2.1. Linear Response Function of Density and Spin Density

As described in the Introduction section, we have investigated the linear response function of the density, because it determines all other electronic properties uniquely in DFT [25].

In the context of QM/MM modeling, the perturbations δv(r′), correspond to the errors, due to the replacement of the QM electronic structures of peripheral and environmental parts (r′) of large molecular systems by MM point charges. The density changes, δρ(r), correspond to the errors in the QM region (r). Then we assume that the following relation holds,

(5) $$\delta \rho \left(r\right)=\int dr\prime \frac{\delta \rho \left(r\right)}{\delta v\left(r\prime \right)}\delta v\left(r\prime \right).$$

We would like to emphasize again that δv(r′) depends on the approximation of the QM/MM modeling for the boundary region, such as the link atom method or the localized orbital method. We do not focus our attention on δv(r′), although many QM/MM developers intend to improve δv(r′) (reference [5] and references therein). Instead, our purpose is to inspect δρ(r)/δv(r′), which is related to how the errors due to the QM/MM model propagate through the target molecule. If we employed a specific approximation (for instance, the link-H atom method), then we can assume that the error due to the approximation, δv(r′), takes a specific value. Then we can regulate the error of the density at the QM event site, δρ(r), by putting the QM/MM boundary (r′) away from the QM event site (r), on the basis of the δρ(r)/δv(r′) value: As the value δρ(r)/δv(r′) decreases, the error of the corresponding QM/MM setting decreases. Another noteworthy point is that, although we assume that the calculation of the whole system can be performed, as shown in the examples in this study, δρ(r)/δv(r′) can also be useful for estimating the effects on the reaction center r on extending the system at a peripheral point r′. Although such calculations are not done below, such type of studies are interesting in relation to the substituent effects, which are often studied in the field of conceptual DFT [23,24]. We also note that density changes are the main cause of errors in dynamic behavior of the systems when we perform the molecular dynamics, based on the QM calculations, because the dynamics is determined by forces acting on the nuclei. According to Feynman’s electrostatic theorem [36], the forces depend only on the positions of nuclei and electron density. In addition, the validity of the QM/MM modeling relies on the near-sightedness of electronic matter (NEM) as proposed by Kohn and Prodan [27], which states that the perturbations, δv, at any points that are far from a specific point, do not change electronic density, δρ, at the point significantly. Thus, our LRF analysis for the QM/MM modeling can be considered a test of whether, and how, NEM holds for molecular systems.

In our analysis, we estimated the LRF by applying the simple perturbation theory to the Kohn-Sham DFT solution as,

(6) $$\frac{\delta \rho \left(r\right)}{\delta v\left(r\prime \right)}=\sum_{\sigma }^{\alpha ,\beta }\frac{\delta \rho_{}^{\sigma }\left(r\right)}{\delta v\left(r\prime \right)}=\sum_{\sigma }^{\alpha ,\beta }\sum_{i}^{N_{occ}^{\sigma }}\sum_{j}^{N_{unocc}^{\sigma }}\frac{\psi_{i\sigma }^{}\left(r\right)\psi_{j\sigma }^{}\left(r\right)\psi_{i\sigma }^{}\left(r\prime \right)\psi_{j\sigma }^{}\left(r\prime \right)}{\varepsilon_{i\sigma }^{}-\varepsilon_{j\sigma }^{}}.$$

Here, $\psi_{i\sigma }^{}\left(r\right)$ and $\psi_{j\sigma }^{}\left(r\right)$ are occupied and unoccupied Kohn-Sham orbitals with spin σ. The first, second, and third summations run over the spin variables occupied orbitals, and all unoccupied orbitals, respectively. Readers should note that the perturbation, δv(r′) is not specified and is virtual. In other words, we do not need any actual perturbations, such as point charges or electric fields to estimate δρ(r)/δv(r′). All we need are Kohn-Sham orbitals and their orbital energies. The use of the LRF is not common, but has been investigated in the field of conceptual DFT. In [22], the authors related the LRF to softness, (S), local softness (s(r)), and the kernel of local softness (s(r,r′)) as, $\delta \rho \left(r\right)/\delta v\left(r\prime \right)=-s\left(r,r^{\prime }\right)+s\left(r\right)s\left(r\prime \right)/S$ and discussed the hard and soft acid and bases (HSAB) principle on the basis of DFT. Geerlings and his coworkers showed that the LRF can be a good descriptor for the inspection of substitution effects, aromaticity, and in the chemical reactivity of molecules [23,24]. We have applied the LRF in order to determine guidelines for the appropriate QM/MM modeling, not to discuss the specific “chemistry” of the target molecules. We intend to make full use of the fact that the LRF values, δρ(r)/δv(r′), strongly depends on the numbers and types of chemical bonds lied between r and r′. For the current purpose, it would be useful to employ the condensed versions of the LRF of density, i.e., the density change at r, due to the perturbation that is applied to the J-th atom, δρ(r)/δv(J) and the density change at the I-th atom for the perturbation to the J-th atom, δρ(I)/δv(J). We describe the computational details of these condensed versions of the LRF of density in the Materials and Methods section below.

The extension of the LRF to the spin density version is straightforward as follows. If we note that the spin density, $\rho_{z}^{}\left(r\right)$, is the difference between α spin density, $\rho_{}^{\alpha }\left(r\right),$ and β spin density, $\rho_{}^{\beta }\left(r\right),$ and that the primary perturbation to the spin density is the virtual magnetic field, $v_{m}$, we have

(7) $$\frac{\delta \rho_{z}^{}\left(r\right)}{\delta v_{m}\left(r\prime \right)}=\frac{\delta \rho_{}^{\alpha }\left(r\right)}{\delta v_{m}\left(r\prime \right)}-\frac{\delta \rho_{}^{\beta }\left(r\right)}{\delta v_{m}\left(r^{\prime }\right)},$$

where the LRF of the σ spin density is given by

(8) $$\frac{\delta \rho_{}^{\sigma }\left(r\right)}{\delta v_{m}\left(r\prime \right)}=\sum_{i}^{N_{occ}^{\sigma }}\sum_{j}^{N_{unocc}^{\sigma }}\frac{\psi_{i\sigma }^{}\left(r\right)\psi_{j\sigma }^{}\left(r\right)\psi_{i\sigma }^{}\left(r\prime \right)\psi_{j\sigma }^{}\left(r\prime \right)}{\varepsilon_{i\sigma }^{}-\varepsilon_{j\sigma }^{}}.$$

This formulation is not new. For instance, Fias et al. formulated the LRF of spin density in terms of $\delta \rho_{}^{\sigma }\left(r\right)/\delta v_{}^{\sigma \prime }\left(r\prime \right)$ for all the combination, $\left(\sigma ,\;\sigma^{\prime }\right)=\left(\alpha ,\;\alpha \right)\;,\left(\alpha ,\;\beta \right)\;,\left(\beta ,\;\alpha \right)\;,\left(\beta ,\beta \right)$ and inspected the LRF of spin density of atoms from the viewpoint of shell-structure and electron-electron repulsion in atoms [37]. Nevertheless, the chemistry, based on the LRF of spin density, has not been investigated thoroughly so far, even in the field of the conceptual DFT. One of the reasons is that many researchers consider the spin-density in Kohn-Sham (KS) DFT as an artifact of the theory: In fact, the KS-DFT solutions, exhibiting a singlet bi-radical do not have spin symmetry, although the spin symmetry condition must be satisfied for the non-relativistic many-electrons systems. Still, we would like to emphasize that the correct description of spin density is essential for understanding electronic structures of transition metal complexes, because it is closely related to the effective bond-order and the radical character of the solutions. In the next section, we will describe the relation between the spin density and the effective bond-order, that can be derived from the KS DFT solutions, and an interpretation of spin density as an indicator of electron-electron correlation effects, that are important for computing transition metal complexes containing open-shell transition metal ions.

### 2.2. Spin Density as an Indicator of Correlation Effects in Kohn-Sham DFT

Closed shell molecules can be described by pairs of electrons in the KS orbitals that share the same spatial distribution. However, the closed-shell picture often breaks down in the case of transition metal complexes, containing open-shell transition metal ions, yielding more stable broken-symmetry (BS) solutions of spin-unrestricted KS-DFT (UKS-DFT) [38,39]. Typically, the BS solutions of UKS-DFT are expressed with the bonding and anti-bonding closed-shell orbitals as,

(9a) $$\psi_{i}^{\alpha }\left(r\right)=cos\theta_{i}\;\phi_{i}^{bond}\left(r\right)+sin\theta_{i}\;\phi_{i}^{antibond}\left(r\right),$$

(9b) $$\psi_{i}^{\beta }\left(r\right)=cos\theta_{i}\;\phi_{i}^{bond}\left(r\right)-sin\theta_{i}\;\phi_{i}^{antibond}\left(r\right).$$

Note that the antibonding orbitals are mixed with the bonding orbitals in BS solutions implying that the BS solutions suffer the instability of chemical bonds of the open-shell species [34]. In fact, the first-order density matrix of the BS solutions given by Equations (9a) and (9b) can be diagonalized in the form,

(10) $$\rho \left(r,r^{\prime }\right)=\sum_{i}^{}\left\{\left(1+cos2\theta_{i}\right)\phi_{i}^{bond}\left(r\right)\phi_{i}^{bond}\left(r^{\prime }\right)+\left(1-cos2\theta_{i}\right)\phi_{i}^{antibond}\left(r\right)\phi_{i}^{antibond}\left(r^{\prime }\right)\right\}\;=\sum_{i}^{}\left(n_{i}^{bond}\phi_{i}^{bond}\left(r\right)\phi_{i}^{bond}\left(r^{\prime }\right)+n_{i}^{antibond}\phi_{i}^{antibond}\left(r\right)\phi_{i}^{antibond}\left(r^{\prime }\right)\right)$$

Thus the effective bond-order (b) can be expressed as

(11) $$b\equiv \sum_{i}^{}b_{i}^{}=\sum_{i}^{}\frac{n_{i}^{bond}-n_{i}^{antibond}}{2}=\sum_{i}^{}cos2\theta_{i}=\sum_{i}^{}T_{i}$$

Here,$\;T_{i}$ is defined as the overlap integral between $\psi_{i}^{\alpha }\left(r\right)$ and $\psi_{i}^{\beta }\left(r\right)$,

(12) $$T_{i}^{}\equiv \langle \psi_{i}^{\alpha }|\psi_{i}^{\beta }\rangle .$$

We should note that $T_{i}^{}$ takes a value from 1 (the closed-shell solutions) to 0 (the completely localized singlet biradical), and so do $b_{i}^{}$. Using Equations (9a) and (9b), together with Equation (12), the spin density can be expressed by

(13) $$\rho_{z}^{}\left(r\right)=2\sum_{i}^{}\sqrt{1-T_{i}^{2}}\;\;\phi_{i}^{bond}\left(r\right)\phi_{i}^{antibond}\left(r\right)$$

If we consider a two-electron two-site model, bonding and antibonding orbitals are given by

(14a) $$\phi_{}^{bond}\left(r\right)=\frac{1}{\sqrt{2}}\left(\chi_{1}^{}\left(r\right)+\chi_{2}^{}\left(r\right)\right)$$

(14b) $$\phi_{}^{antibond}\left(r\right)=\frac{1}{\sqrt{2}}\left(\chi_{1}^{}\left(r\right)-\chi_{2}^{}\left(r\right)\right)$$

and the spin density reduces to

(15) $$\rho_{z}^{}\left(r\right)=\sqrt{1-b^{2}}\;\chi_{1}^{}\left(r\right)\chi_{1}^{}\left(r\right)-\sqrt{1-b^{2}}\;\chi_{2}^{}\left(r\right)\chi_{2}^{}\left(r\right).$$

The first and the second terms on the right side are the spin densities on the 1st, and the 2nd sites, respectively. Equation (15) implies that the spin density includes the information concerning the effective bond-order for radical systems. A simple spin-projection method to recover the spin-symmetry of the BS KS-DFT solutions has also been derived [34,38,39]. In this case, the effective bond-order of the spin-projected solution is rewritten in the form, $B=\sum_{i}^{}2T_{i}/\left(1+T_{i}^{2}\right)$. As an index for the radical character of the spin-projected solutions, the di-radical character is defined as y≡1−B [34,35]. Recently, the di-radical character was used to design molecular materials exhibiting hyper-polarizability [40]. However, such properties based on the spin-projection procedure are beyond the scope of this paper. Instead, we note the implication of spin density of the BS-KS-DFT solutions, as pointed out by Perdew et al.: spin density is an alternative variable of the on-top pair density, which is an indicator of the correct description of correlation effects between α and β spins for magnetic systems [32], via the following relation,

(16) $${\rho_{2}^{\alpha \beta }\left(r;r\prime \right)|}_{r=r\prime }=\frac{1}{2}\left(\rho \left(r\right)\rho \left(r\right)-\rho_{z}^{}\left(r\right)\rho_{z}^{}\left(r\right)\right)$$

This property indicates the degree of correlation between α and β electrons. Whether the spin-projection is applied to the BS KS-DFT solution or not, it is needed to describe the spin-polarized state of transition metal complexes appropriately. This is the reason why we focus on spin density. Of course, for closed shell systems, the BS KS-DFT solutions reduce to the spin restricted KS-DFT solutions. Thus the spin density, di-radical character, and the on-top pair density reduce to zero. Of course, $\delta \rho_{z}^{}\left(r\right)/\delta v_{m}\left(r\prime \right)$ also vanishes. In the next section, we will present the computational results of the reaction centers of metallo-protein that contain open-shell transition metal ions. We will focus on the use of the LRF of density and spin density to determine the QM/MM modeling to obtain the appropriate KS-DFT solutions. 

## 3. Results and Discussion

We will present the computational results for two types of transition-metal complexes. For all computations of LRFs, we used a modified version of GAMESS [41]. The B3LYP functional is employed for all DFT calculations [42]. Basis sets for each transition metal complexes and other computational details are described in Materials and Methods section.

### 3.1. Heme System~Reaction Center of P450

#### 3.1.1. The Target System

**Figure 1:**
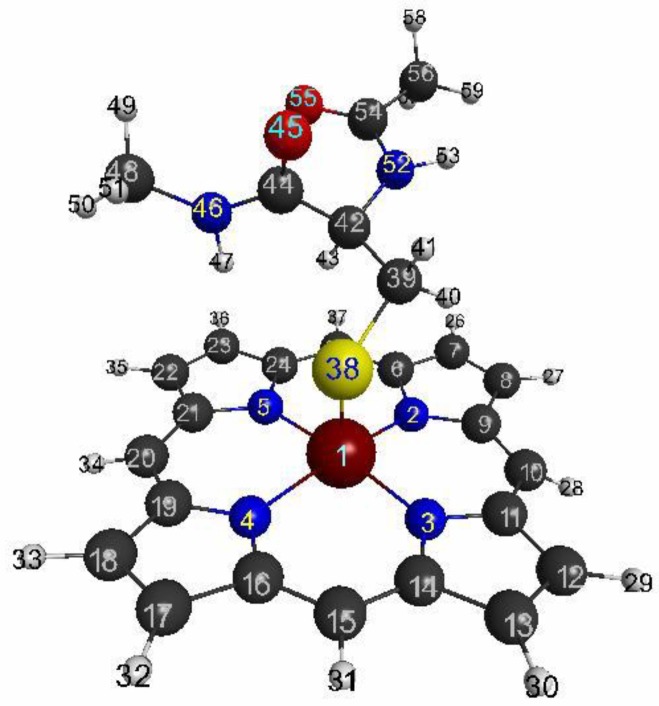
Geometry of the active site of P450 (pdb entry:5vws). The numbering of the atoms is used for the following figures to show linear response functions.

Metalloporphyrins play various roles, such as transportation of O_2_, catalase, monooxidase, and electron transfer in living bodies [43]. Thus, as a typical mono nuclear transition metal complex, we chose metalloporphyrin, that is extracted from the active center of P450 (pdb entry:5vws) [44], of which the geometry is shown in Figure 1. The same, but a larger picture is shown in Figure S1. The numbers, indicated on the atoms in the figures, are used in the following visualizations of LRFs and the discussion. To confirm the numbering of the atoms, please refer to Figure S1.

**Figure 2:**
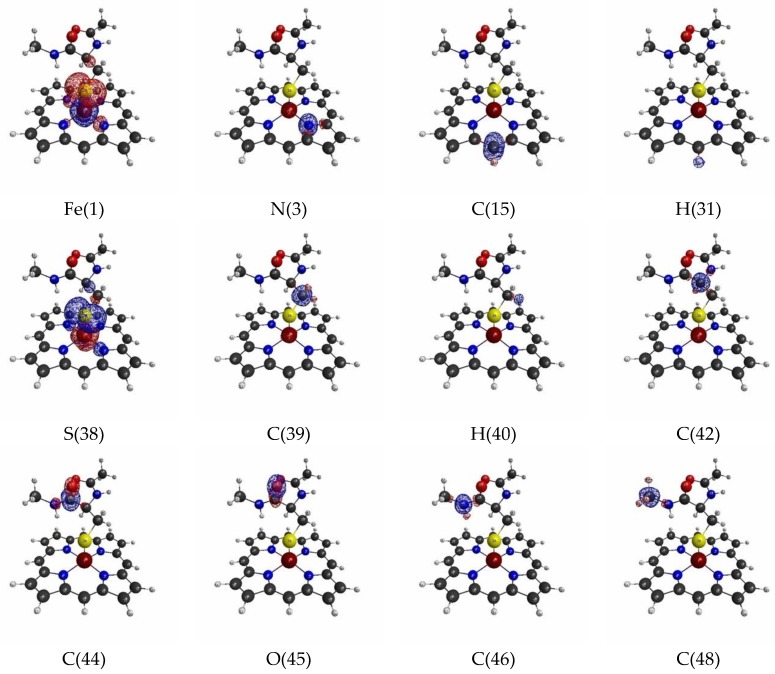
Isosurfaces of linear response functions of density for the perturbations on atomic sites of P450 (pdb entry:5vws). The threshold of δρ(r)/δv(I) is ±0.01 (+: blue, −: red). The atomic symbols and those numbers in parentheses below the figures indicate the atoms (I) to which the virtual perturbations are applied.

The isosurfaces of the LRFs for the threshold setting with δρ(r)/δv(I)=±0.1 to the perturbations that are applied to the atomic sites are shown in Figure 2. In the context of QM/MM modeling, roughly speaking, the region where the responses appear in this figure should be included in the QM region for this threshold. As we can see from Figure 2, almost all responses are localized near the perturbation site. However, when the perturbation is applied to the Fe ion (Fe(1)), the density changes are obviously spread over the S ion of the thiol group, and vice versa. This implies that the Fe(III) and S(-I) are strongly coupled to each other. In fact, it is known that the part of residues ligated from the above and/or below sides regulate the functionality of the Heme core, and the Cys of P450 is a typical example [43]. Interestingly, although δρ(r)/δv(Fe(1)) is also delocalized over the porphyrin ring (Por), the coupling between Fe(III) and S ions seems to be stronger than that between Fe(III) and the porphyrin ring from the viewpoint of LRF of electron density. Also, we note that the perturbation to a site in a specific amide plane yields density changes over the atoms in the same plane (for instance, C(42), C(44), O(45)). In other words, the LRF with this threshold detects the firm units, where the sites are coupled with strong covalent and/or ionic bonds.

#### 3.1.2. Linear Response Functions of Electron Density of P450

**Figure 3:**
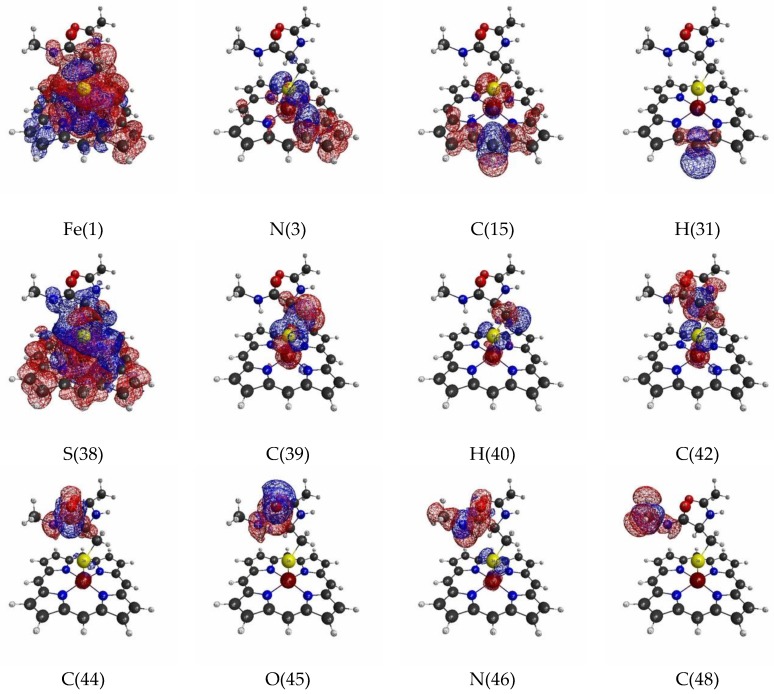
Isosurfaces of linear response functions of density for the perturbations on atomic sites of P450 (pdb entry:5vws). The threshold of δρ(r)/δv(I) is ±0.01 (+: blue, −: red). The atomic symbols and those numbers in parentheses below the figures indicate the atoms (I) to which the virtual perturbations are applied.

Next, Figure 3 shows the isosurfaces of the LRFs with the threshold δρ(r)/δv(I)=±0.01. As suggested in the Theoretical Background section, as the value of the threshold decreases, a large QM region is required to satisfy the threshold of the errors on density. In this “tighter” threshold, the responses naturally become more delocalized than those for δρ(r)/δv(I)=±0.1. The result for when the perturbation is applied to Fe(III) shown in Figure 2 (δρ(r)/δv(Fe(1))) is remarkable. Because the Fe(III) and S(-I) ions are coupled with strong ionic bonds, the responses are spread over the heavy atoms in the porphyrin ring, together with the residues of amino acids up to the N(46) atom. Another point of interest is that the propagation of the effects are spread over through the two sp3 junctions (C(39) and C(42) atoms). This must be via the hyperconjugation path consisting of S(38), C(39), C(42), and C(44) atoms. The interaction between the plus charge at H(47) in the NH and the minus charge at S(38) must also facilitate this delocalization. In fact, the δρ(r)/δv(N(46)) is also spread to the S(-I) site. As pointed out above, because the Fe(III) and S(-I) are strongly coupled with each other, the extensive delocalization of the density fluctuations is also observed in δρ(r)/δv(S(38))=±0.01. Compared to the case of the loose threshold (δρ(r)/δv(I)=±0.1), there are other differences. First, the considerably strong coupling between Fe(III) and the heavy atoms in the porphyrin ring is observed for δρ(r)/δv(C(15)). Second, the delocalization effects over hyperconjugation through the sp3 junctions at the C carbons are also observed (δρ(r)/δv(C(39)), δρ(r)/δv(C(40)), (r)/δv(C(42)). In summary, the LRF with this threshold detects the hyperconjugation and weak bonds in the whole molecule, by which the electronic structure of the whole molecule must be regulated. Thus, it is strongly recommended that the coupled units presented in our computational results of δρ(r)/δv(I)=±0.01 are not be divided or partially replaced by capped H atoms. For instance, from δρ(r)/δv(S(38))=±0.01 shown in Figure 3, the replacement of the terminal C(48)H_3_ by H is acceptable, but the replacement of the C(42)N(52)H(43)C(44) by CH_3_ should be avoided.

**Figure 4:**
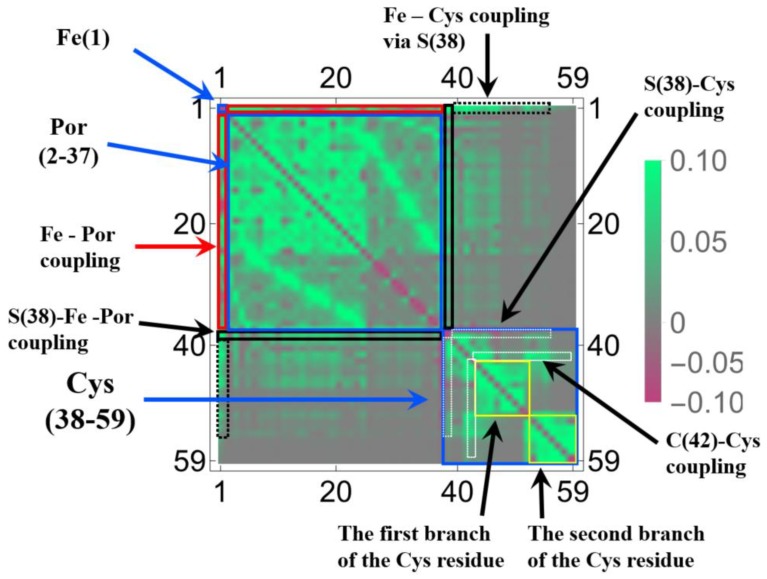
The heat map for the matrix representation of condensed version of linear response functions of electron density,$\;{\left\{\delta \rho \left(I\right)/\delta v\left(J\right)\right\}}_{I,J=1∼59}$, for P450. The number of the axes corresponds to the numbering of the atoms indicated in Figure 1. The functional units and the couplings among units are also indicated (see text for the details).

**Figure 5:**
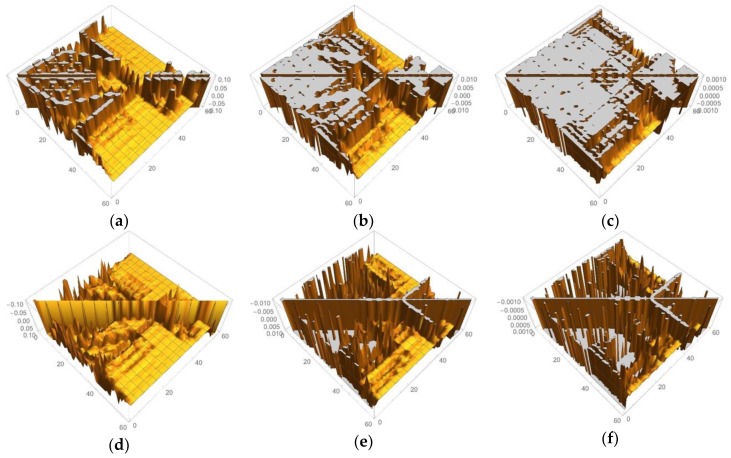
Matrix representation of condensed version of linear response functions of electron density for P450 with the six cutting thresholds. (**a**) δρ(I)/δv(J)<0.1. (**b**) δρ(I)/δv(J)<0.01. (**c**) δρ(I)/δv(J)<0.001, (**d**) δρ(I)/δv(J)>−0.1. (**e**) δρ(I)/δv(J)>−0.01. (**f**) δρ(I)/δv(J)>−0.001.

In Figure 1 and Figure 2, we selected the important isosurfaces of LRFs to reduce the numbers of the figures. For more comprehensive data (but with almost equivalent figures removed), please see Figures S2 and S3. The tighter the threshold, the more delocalized isosurfaces are observed. We also plotted δρ(r)/δv(I)=±0.001 in Figure S4. For convenience, the more compact representation of Figures S2–S4 is shown in Figure 4 as a heat-map representation of the matrix, ${\left\{\delta \rho \left(I\right)/\delta v\left(J\right)\right\}}_{I,J=1∼59}$. It is obvious from this figure that there are three units, the Fe ion, the porphyrin ring (Por), and the His residue as the diagonal blocks, which are indicated by blue squares. The strong coupling between the Fe ion and the Por ring, which is described above, and the coupling between the Por ring and the His residue are indicated in black rectangles. A noteworthy point is that the perturbation to Fe(1) is spread not only to Por, which is indicated by the red square, but even into His via S(38), which is indicated by the dotted black rectangles. These dotted black rectangles correspond to isosurfaces of δρ(r)/δv(Fe(1))=±0.01 and δρ(r)/δv(S(38))=±0.01 shown in Figure 2. One of the reasons for such a delocalized character of δρ(r)/δv(Fe(1)) can be explained as follows. In the diagonal block that corresponds to the Cys residue, we can see sub-structures indicated by yellow squares, which correspond to two branches of the Cys residue (C(44)-H(50)) and (C(52)-H(59)). There are two major types of off-diagonal blocks in this diagonal block of the Cys residue: One is between S(38) and these two branches, and another is between the root of the two branches, i.e., C(42), and the two branches, both of which are shown as dotted white rectangles. Obviously, these paths mediate the propagation of the effects, due to the perturbation from the Fe(1)-S(38) unit to the two blanches of the Cys residue. In order to discuss the relationship between the structures of the LRF and the threshold for the LRF values more clearly, we plotted the sections for δρ(I)/δv(J)=0.1, δρ(I)/δv(J)=0.01, and δρ(I)/δv(J)=0.001 in Figure 5a–c, respectively. For completeness, we also plotted δρ(I)/δv(J)=−0.1, δρ(I)/δv(J)=−0.01, and δρ(I)/δv(J)=−0.001 in Figure 5d–f, respectively. Because the minus value of δρ(I)/δv(J) indicates an increase in electron density, δρ(I), due to the attractive perturbation, δv(J), the section for δρ(I)/δv(J)=−0.1 is localized at the diagonal part as shown in Figure 5d. Thus, the delocalization of the LRFs is more clearly seen from Figure 5a–c than from Figure 5d–f. We can see from Figure 5a that if we employ the criterion, |δρ(I)/δv(J)|<0.1, this system can be divided into several parts. However, because the Fe(III) and S(-I) ions are strongly coupled, the Fe(III) and Por cannot be separated from the Cys residue as shown in Figure 2 (δρ(r)/δv(Fe(1))=±0.1 and δρ(r)/δv(S(38))=±0.1). This characteristic of the P450 active site is more explicitly shown in Figure 5b, which implies that the terminal CH_3_ beyond C(48) and the terminal CH_3_CO beyond N(52) can be replaced by capped H atoms. When the criterion |δρ(I)/δv(J)|<0.001 must be imposed, the whole system should be included in one QM region, which can be seen from the spread of the section shown in Figure 5c. Roughly speaking, however, this criterion means that even when the QM/MM modeling error δv(J) is 1 Hartree, the error on the active site δρ(I) is less than 0.001, which is too severe criterion. The approximation to exchange-correlation functional of DFT might lead to larger errors than the QM/MM modeling with the criterion (|δρ(I)/δv(J)|<0.001). In fact, almost all QM/MM researchers include up to the second-next residues from the reaction centers (Ref. [5] and references therein), being consistent with the criterion, |δρ(I)/δv(J)|<0.01.

#### 3.1.3. Linear Response Functions of Spin Density of P450

**Figure 6:**
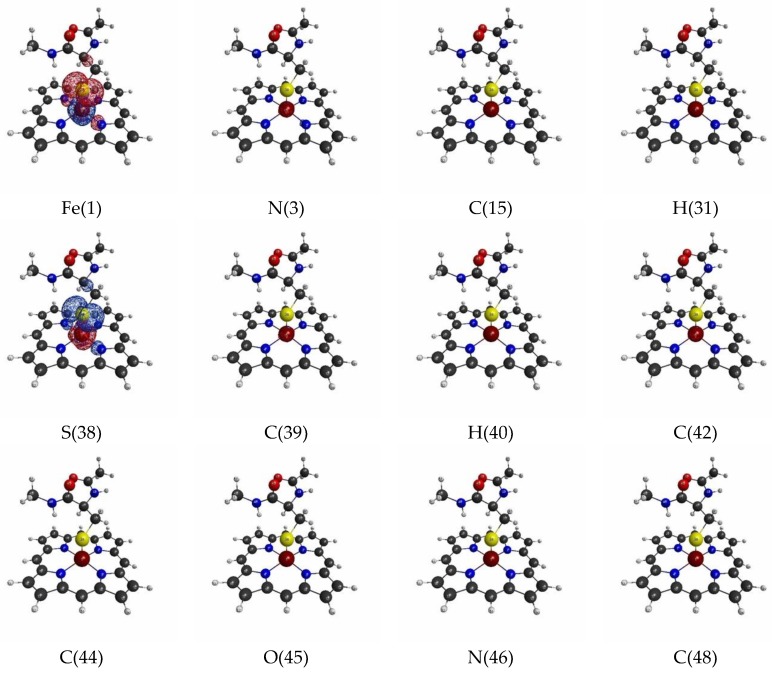
Isosurfaces of linear response functions of spin density for the perturbations on atomic sites of P450). The threshold of $\delta \rho_{z}\left(r\right)/\delta v_{m}\left(I\right)$ is ±0.1 (+: blue, −: red). The atomic symbols and those numbers in parentheses below the figures indicate the atoms (I) to which the virtual perturbations are applied.

**Figure 7:**
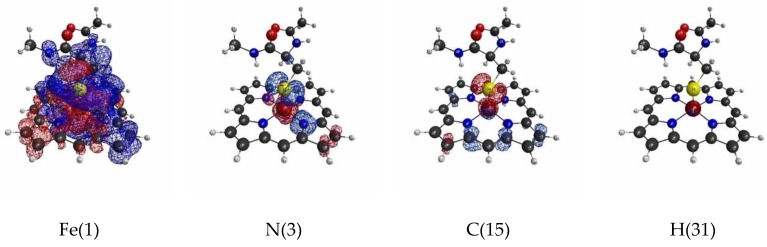

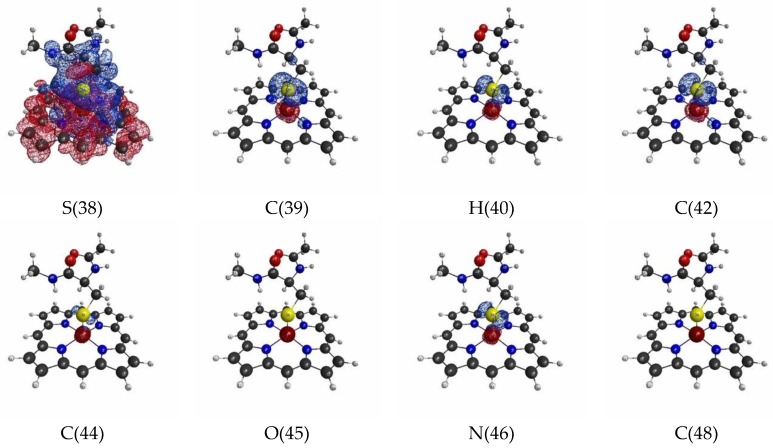
Isosurfaces of linear response functions of spin density for the perturbations on atomic sites of P450). The threshold of $\delta \rho_{z}\left(r\right)/\delta v_{m}\left(I\right)$ is ±0.01 (+: blue, −: red). The atomic symbols and those numbers in parentheses below the figures indicate the atoms (I) to which the virtual perturbations are applied.

Next, we plotted isosurfaces of LRFs of spin density with $\delta \rho_{z}\left(r\right)/\delta v_{m}=\pm 0.1$ in Figure 6. As we can see, except $\delta \rho_{z}\left(r\right)/\delta v_{m}\left(\mathrm{Fe}\left(1\right)\right)\;$and $\delta \rho_{z}\left(r\right)/\delta v_{m}\left(S\left(38\right)\right)$, the responses are not remarkable. Still, $\delta \rho_{z}\left(r\right)/\delta v_{m}\left(\mathrm{Fe}\left(1\right)\right)$ and $\delta \rho_{z}\left(r\right)/\delta v_{m}\left(S\left(38\right)\right)$ are delocalized into the porphyrin ring and C(42) of the Cys residue. The sensitivity of the sites, Fe(1) and S(38), becomes more obvious for the threshold $\delta \rho_{z}\left(r\right)/\delta v_{m}\left(I\right)=\pm 0.01$ as shown in Figure 7.

**Figure 8:**
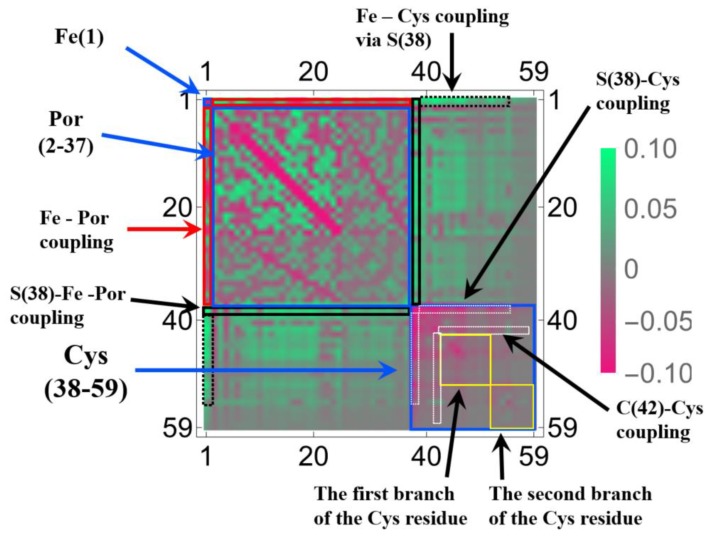
The heat map for the matrix representation of condensed version of linear response functions of the spin density ${\left\{\delta \rho_{z}^{}\left(I\right)/\delta v_{m}\left(J\right)\right\}}_{I,J=1∼59}$ for P450. The number of the axes corresponds to the numbering of the atoms indicated in Figure 1. The functional units and the couplings among units are also indicated (see text for the details).

**Figure 9:**
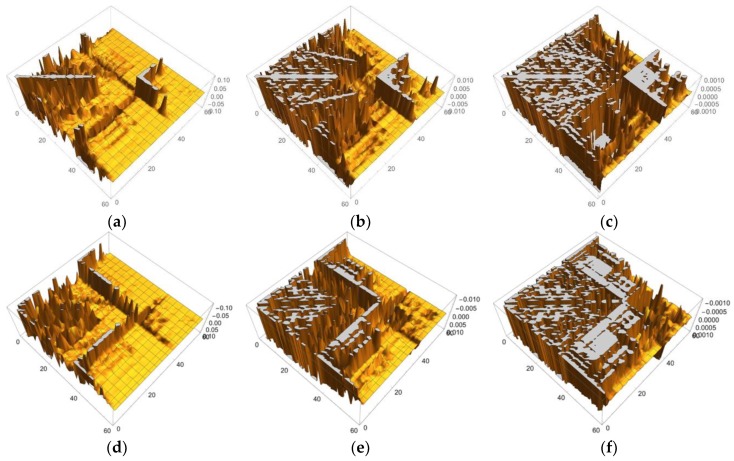
Matrix representation of condensed version of linear response functions of spin density for P450 with the six cutting thresholds. (**a**) $\delta \rho_{z}^{}\left(I\right)/\delta v_{m}\left(J\right)<0.1.$ (**b**) $\delta \rho_{z}^{}\left(I\right)/\delta v_{m}\left(J\right)<0.01$. (**c**) $\delta \rho_{z}^{}\left(I\right)/\delta v_{m}\left(J\right)<0.001$, (**d**) $\delta \rho_{z}^{}\left(I\right)/\delta v_{m}\left(J\right)>-0.1.$ (**e**) $\delta \rho_{z}^{}\left(I\right)/\delta v_{m}\left(J\right)>-0.01$. (**f**) $\delta \rho_{z}^{}\left(I\right)/\delta v_{m}\left(J\right)>-0.001$.

For reference, Figures S5–S7 plot the comprehensive data of the isosurfaces for $\delta \rho_{z}\left(r\right)/\delta v_{m}\left(I\right)=\pm 0.1$, $\delta \rho_{z}\left(r\right)/\delta v_{m}\left(I\right)=\pm 0.01$, and$\;\delta \rho_{z}\left(r\right)/\delta v_{m}\left(I\right)=\pm 0.001$, respectively. The results for $\delta \rho_{z}\left(r\right)/\delta v_{m}\left(I\right)=\pm 0.001$ shown in Figure S7 are similar to but more compact than those of δρ(r)/δv(I) shown in Figure S4. It should be noted that $\delta \rho_{z}\left(r\right)/\delta v_{m}\left(H\left(47\right)\right)\;$and $\delta \rho_{z}\left(r\right)/\delta v_{m}\left(N\left(46\right)\right)\;$shown in Figure 7 and $\delta \rho_{z}\left(r\right)/\delta v_{m}\left(N\left(52\right)\right)$ shown in Figure S6 are spread over Fe and S. On the other hand, the perturbations that are applied to terminal CH_3_ beyond C(48) and the terminal CH_3_CO beyond N(52) do not significantly affect Fe or S: This is similar to the linear response function (LRF) of electron density. On the basis of these results, the same conclusion can be drawn from the viewpoint of the use of both LRFs of density and spin density for QM/MM modeling of this system: the CH_3_ beyond C(48) and the terminal CH_3_CO beyond N(52) can be approximated as MM charges. However, the overview of matrix representation of ${\left\{\delta \rho_{z}^{}\left(I\right)/\delta v_{m}\left(J\right)\right\}}_{I,J=1∼59}$, which can be seen from Figure 8, is considerably different from the corresponding figure of the LRF of density shown in Figure 4. The first remarkable point is that the disordered checker pattern can be seen in the sub-block of the porphyrin, which indicates the alternative spin-polarization effects on the porphyrin ring. This can be also confirmed from sections shown in Figure 9a–f. For instance, the sections of Figure 9b,e compensate each other in the region of the porphyrin ring. This is the result of the alternative spin polarization effects. It is essentially parallel to the spin-polarization effects on the π-conjugation network, which has been explored in the spin-alignment rule for molecular magnetism by Yamaguchi and his coworkers [45]. The second point is that there is an anti-parallel spin-polarization effect between S(38) and Fe(1) + Por. For instance, we can observe that the large parts of the off-diagonal blocks between S(38) and Fe(1) + Por are colored in green ($\delta \rho_{z}^{}\left(I\right)/\delta v_{m}\left(S\left(38\right)\right)=-0.1$). We also notice that the dull red color is spread over most of the Cys residue as shown in Figure 8. This is because the orthogonality among sp3 orbitals causes the weakly high-spin interactions, but of which the magnitudes are quite weak, resulting in the “dull” red color. In contrast, the off-diagonal parts between S(42) and the Cys residue, which are indicated by dotted white rectangles, are considerably clear red color. The result can be more clearly seen in the L-shaped section in the region of S(38)~N(46)H(47) in Figure 9a. This section is increasingly delocalized towards two terminal carbons of the two branches of the Cys residues, as the thresholds increases to $\delta \rho_{z}^{}\left(I\right)/\delta v_{m}\left(J\right)<0.01$ (Figure 9b) and further to $\delta \rho_{z}^{}\left(I\right)/\delta v_{m}\left(J\right)<0.001$. This is consistent with the spin density distribution of the whole system as will be shown in the following discussion: In fact, the spin distribution on S(42) is approximately 0.6, and such up-spin density is slightly delocalized over the Cys residue, indicating that there is originally weak high-spin coupling between S(42) and the Cys residue.

#### 3.1.4. QM Cluster and QM/MM Calculations for Several Models of P450

**Figure 10:**
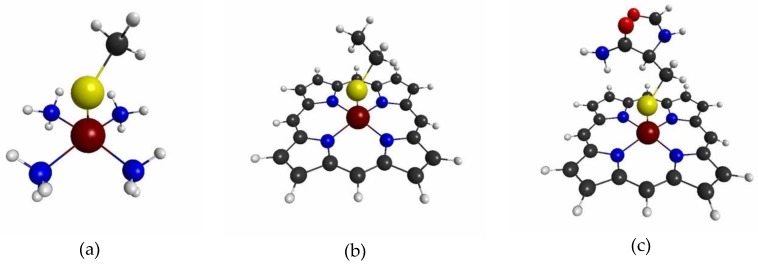
Geometries of the three models for the active site of P450: (**a**) model 1, (**b**) model 2, (**c**) model 3. We assumed that the full model is the model shown in Figure 1.

Next, we will show the actual QM/MM calculation results for several models shown in Figure 10. Note that we assumed that the reaction center (RC) of P450 is the Fe(1)S(38) unit. Model 1 shown in Figure 10a is the simplest model, where the porphyrin ring is approximated as four NH_3_ molecules that ligate to the Fe ion and the Cys residue is approximated as CH_3_S^−^ ion. In model 2, we included the porphyrin ring completely, but approximate the Cys residue as C_2_H_5_S^-^ ion. Model 3 suits the criteria

(17) $$\left|\delta \rho \left(I\right)/\delta v\left(RC\right)\right|<0.01,\;\;\;\left|\delta \rho_{z}\left(I\right)/\delta v_{m}\left(RC\right)\right|<0.01$$

the most. Here “RC” denotes the reaction center, where the accurate description of the density and spin density is required. Then, Equation (17) determines the peripheral atoms that should be included in the QM region. In all of the models, we replaced the original parts in the full model by capped H atoms. We computed both the QM cluster model and the QM/MM model, with the link atom method for models 1–3 [3,4,5]. The details of the latter calculations are described in the Materials and Methods section.

The calculated densities and spin densities of the active site are compared among these models and the full model shown in Figure 1. We assumed that the densities and spin densities of the full model are the reference values. We took the differences between the values calculated for models 1–3 and the reference values as the errors due to the modellings. Then, we listed the errors of densities on the Fe ion, the N atoms of the porphyrin ring, and the S ion of the Cys residue in Table 1, and the errors of spin densities for the same set of atoms Table 2. For the complete data of the errors on densities and spin densities, please refer to Tables S1 and S2, respectively. We can see from tables Table 1 and Table 2, that the errors due to the simplest model (Model 1), are much larger than other two models. Furthermore, in model 1, the MM charges do not improve the results in general. On the other hand, the QM cluster models based on the models 2 and 3 reduce the errors on both densities and spin densities drastically. In particular, the errors of the model 3 with QM cluster model are less than 0.005, which indicates that model 3 is nearly equivalent to the full model. The reader might notice that there remain slightly larger errors in the results of the QM/MM model, based on model 3. These results are consistent with two well-known facts of the QM cluster and the QM/MM approaches [5]: First, the QM/MM results show more slower convergence behavior than the QM cluster results as the QM size increases. Second, the inclusion of the environmental effects, by a polarized model or a MM model, requires special care for the modeling and the parameters used. These are the reasons for some cases, where the QM cluster model outperforms the QM/MM model. In this case, the chains of the Cys residue lie parallel to the porphyrin plane, and the point charges of the QM/MM model that mimic the CH_3_ beyond C(48), and the CH_3_CO beyond N(52) might slightly induce the polarization effects on the reaction center in model 3. We would like to emphasize the fact that model 3, by employing the QM cluster model, yields the results that are nearly equivalent to those of the full model. This fact implies that the boundary of the QM model according to the criteria, |δρ(I)/δv(RC)|<0.01 and $\left|\delta \rho_{z}^{}\left(I\right)/\delta v_{m}\left(\mathrm{RC}\right)\right|<0.01$, as suggested above is the appropriate boundary for calculations of the reaction center of P450. 

### 3.2. Oxygen Evolving Complex of Photosystem II.

#### 3.2.1. The Target System

**Figure 11:**
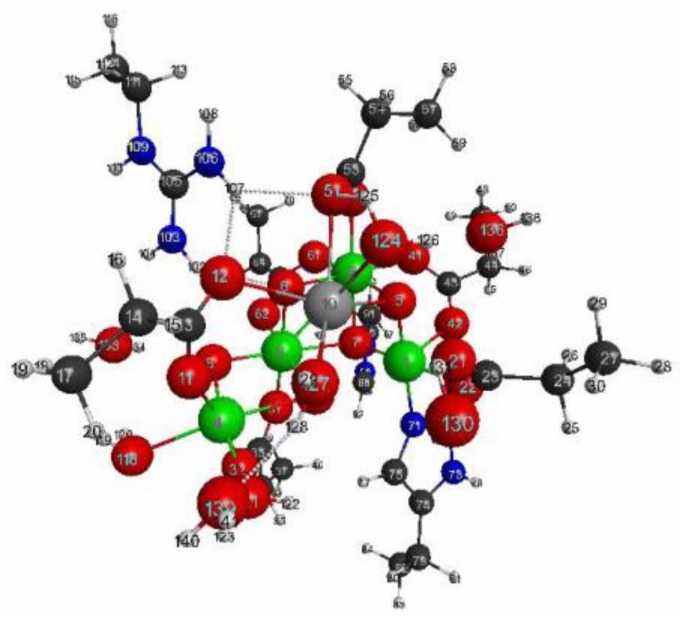
Geometry of the active site of oxygen evolving complex (OEC) (pdb entry:5b66). The numbering of the atoms is used for the following figures to show linear response functions. The larger picture is shown in Figure S8.

Next, we will examine the case of polynuclear complexes embedded in proteins. Di-μ-oxo bridged polynuclear Mn complexes, embedded in proteins, are also widely seen structural units for the reaction centers of biocatalyses, such as catalase [46], superoxide dismutase [47], and the oxygen evolving complex (OEC) in photosystem II [48,49,50,51]. Here, we choose the OEC geometry, that is extracted from the X-ray structure of the dark stable PS-II (pdb entry: 5b66) [48], of which the geometry is shown in Figure 11. The same picture, with the larger size, is shown in Figure S8 for checking the numberings of the atoms. In accordance with previous research on OEC [50,51], we assumed that the ground-state electronic configuration of the core of OEC in the dark stable (S_1_) state is Mn(III)Mn(IV)Mn(IV)Mn(III)Ca(II), which are bridged by di-μ-oxo ions (O^2^^−^) and carboxylates. All oxygen atoms that are not the parts of neither the cluster nor amino residues are assumed to be those of water molecules. Other modeling details are described in Materials and Methods section.

#### 3.2.2. Linear Response Functions of Electron Density of OEC

**Figure 12:**
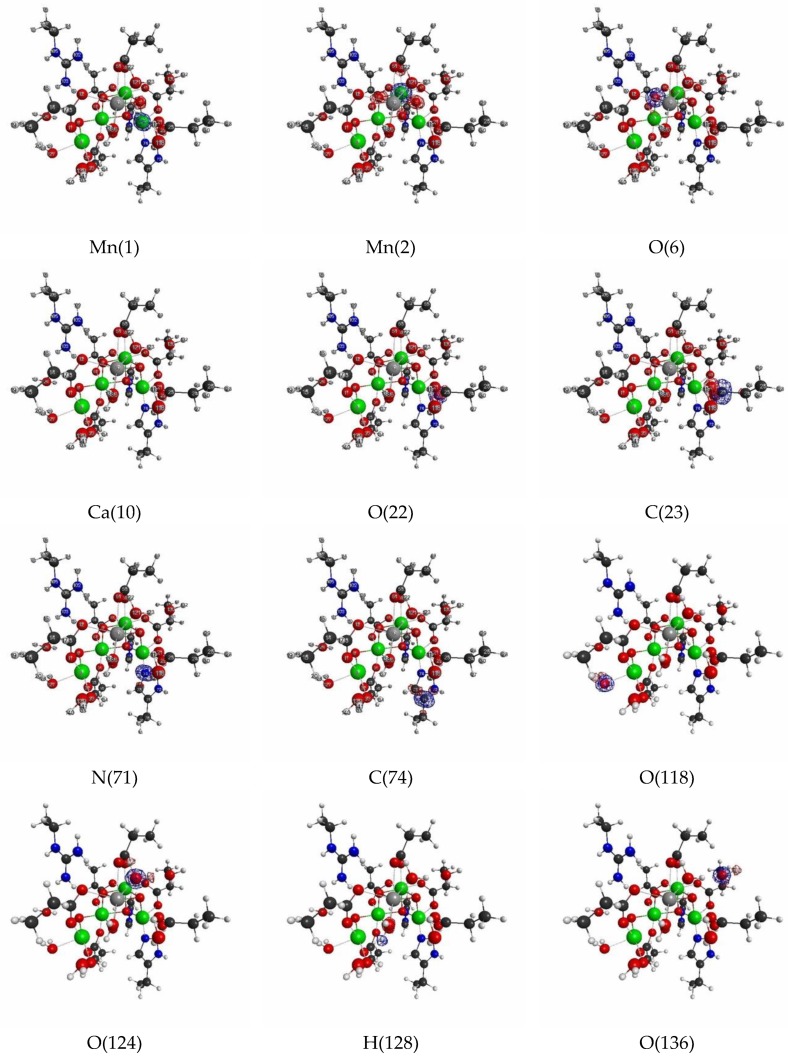
Isosurfaces of linear response functions of density for the perturbations on atomic sites of OEC (pdb entry:5b66). The threshold of δρ(r)/δv(I) is ±0.1 (+: blue, −: red). The atomic symbols and those numbers in parentheses below the figures indicate the atoms (I) to which the virtual perturbations are applied.

**Figure 13:**
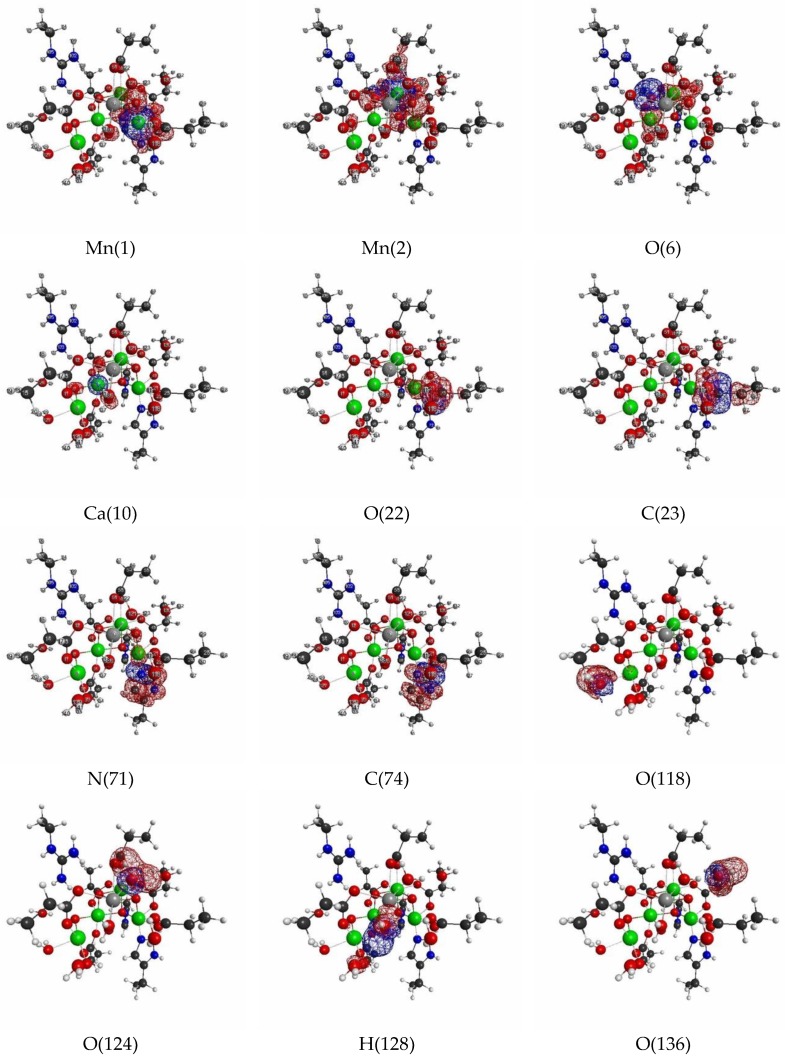
Isosurfaces of linear response functions of density for the perturbations on atomic sites of OEC (pdb entry:5b66). The threshold of δρ(r)/δv(I) is ±0.01 (+: blue, −: red). The atomic symbols and those numbers in parentheses below the figures indicate the atoms (I) to which the virtual perturbations are applied.

In Figure 12 and Figure 13, we show the selected essential isosurfaces of LRFs for our discussion. Again, the thresholds for the isosurfaces are set to be δρ(r)/δv(I)=±0.1 and δρ(r)/δv(I)=±0.01, where the variable I indicates the atom to which the virtual perturbation is applied. We only show the selected isosurfaces, due to the limits of space, but more comprehensive figures of the isosurfaces are shown in Figures S9 and S10 for δρ(r)/δv(I)=±0.1, and δρ(r)/δv(I)=±0.01, respectively. We can see from Figure 12 that even if the perturbation is applied to either the Mn, or the O ions, in the core cluster (δρ(r)/δv(Mn(2)) and (δρ(r)/δv(O(6))), the density fluctuation propagates at most to the nearest ions for the threshold, δρ(r)/δv(I)=±0.1. This implies that even the partitioning of the core cluster is possible at this approximation level. This is a very rough approximation, but the speculated mono- and di-nuclear models of OEC in the 1990s can be considered examples of such models (δρ(r)/δv(I)=±0.1) [52], which were inevitable back then, because even the number of ions consisting the core cluster of the OEC was not completely determined before 2010 [48]. On the other hand, as shown in Figure 10, the isosurface for δρ(r)/δv(Mn(2))=±0.01 spreads beyond C(55), the carbon atom of the carboxylate that bridges between Mn(2) and Ca(10), and reaches at the next sp3 junction, C(54). This implies that the approximation of glutamic acid and aspartic acid residues, as formic acids, is not appropriate. In fact, many researchers model glutamic acid or aspartic acid residues as acetic acids [5,50,51]. Note that the isosurface for δρ(r)/δv(Mn(3))=±0.001 are slightly delocalized to the two sp3 junctions away, C(47) from the C(43)O(41)O(42) carboxylate, as shown in Figure S10. Thus, “the propionic acid” approximation for the carboxylate type of residues is appropriate, if we employ the criterion, δρ(r)/δv(I)=±0.001. Conversely, we can see from δρ(r)/δv(C(23)), where O(22) of the carboxylate, C(23)O(22)O(24), ligates to Mn(1), δρ(r)/δv(C(23)) is delocalized over Mn(1) as shown in Figure 13. In contrast, the coupling between His residue and Mn ion is smaller than that between Glu or Asp residue and Mn ion as shown in δρ(r)/δv(N(71)) and δρ(r)/δv(C(74)) in Figure 13.

As for the interaction between water molecules and the OEC cluster, there are three possibilities: (i) Mn ion and water molecule, (ii) Ca ion and water molecule, and (iii) oxygen ion and water molecule. However, in this structure, there is no type (iii) of the interactions. From the our LRF analysis, type (i) interaction is detected by the threshold, δρ(r)/δv(I)=±0.01, as shown in δρ(r)/δv(O(118)), but type (ii) is not as shown in δρ(r)/δv(124) in Figure 13, Of course, the later interaction is detected by the threshold, δρ(r)/δv(O(124))=±0.001 as shown in Figure S10. Nevertheless, whether a hydrogen bond between water molecules is detected or not depends on the positions and orientations of the two water molecules: See δρ(r)/δv(128) and δρ(r)/δv(136) in Figure 13. Further the extensive hydrogen bonding network over many water molecules and other anionic or cationic parts of residues and cluster are detected by the threshold, δρ(r)/δv(I)=±0.001: see, for instance δρ(r)/δv(O(127)) in Figure S10. Thus, if the QM/MM modeling is to search for the reaction mechanism, involving movements of water molecules near the cluster, it is better to include all water molecules in the QM region.

**Figure 14:**
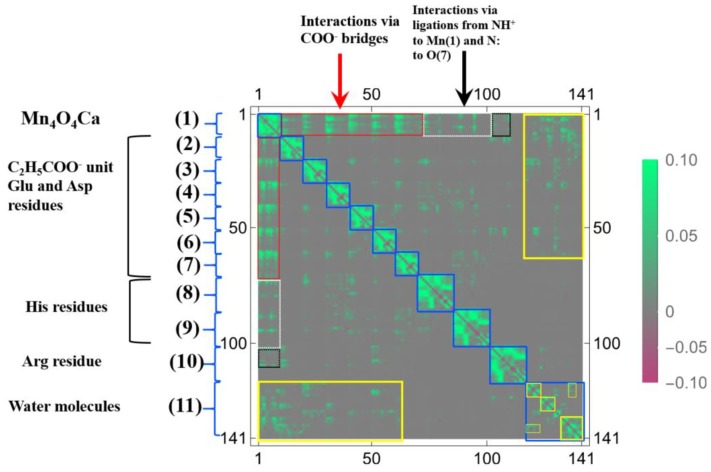
The heat map for the matrix representation of condensed version of linear response functions of electron density, ${\left\{\delta \rho \left(I\right)/\delta v\left(J\right)\right\}}_{I,J=1∼141}$, for OEC. The number of the axes corresponds to the numbering of the atoms indicated in Figure 11. The functional units and the couplings among units are also indicated (see text for the details).

The more compact representation of Figures S9–S11 is shown in Figure 14 as a heat-map representation of the matrix, ${\left\{\delta \rho \left(I\right)/\delta v\left(J\right)\right\}}_{I,J=1∼141}$. Obviously we can see from this figure that there are 11 diagonal sub-blocks, corresponding to the Mn_4_O_5_Ca core (1), the amino residues (2)~(10), and the group of water molecules (11), which are encircled by blue squares. As for the interactions among the sub-blocks, we first noticed the periodic structures encircled in the red rectangles. These are the coupling units where carboxylates bridge Mn and Mn, or Mn and Ca, in the Mn_4_O_5_Ca core. Among them, the off-diagonal part between (1) and (3) corresponds to δρ(r)/δv(C(23)), shown in Figure 13. Also the interactions between the Mn_4_O_5_Ca core and the His residues via the ligations of NH^+^ to Mn(1) and N to O(7), are observed in the white rectangles. In addition, the interactions between the Arg residue and the Mn_4_O_5_Ca core are also observed, but the magnitudes of them are considerably weaker, as shown in the dotted black rectangles. In the sub-block consisting of water molecules (11), there are several hydrogen bonding networks, which are indicated by the small yellow rectangles and squares. In addition, there are other hydrogen bonding networks in the off-diagonal region that are encircled by yellow rectangles, which includes the ligations of the water molecules to Mn and to Ca, respectively as δρ(r)/δv(O(118)) and δρ(r)/δv(O(124)) shown in Figure 13.

**Figure 15:**
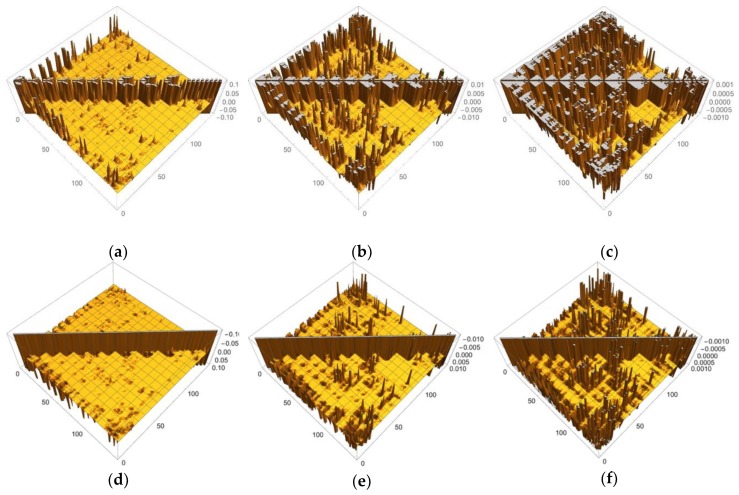
Matrix representation of condensed version of linear response functions of electron density for OEC with the six cutting thresholds. (**a**) δρ(I)/δv(J)<0.1. (**b**) δρ(I)/δv(J)<0.01. (**c**) δρ(I)/δv(J)<0.001, (**d**) δρ(I)/δv(J)>−0.1. (**e**) δρ(I)/δv(J)>−0.01. (**f**) δρ(I)/δv(J)>−0.001.

In order to discuss our results from the viewpoint of the QM/MM modeling more clearly, the sections of the LRF of the density with six thresholds are summarized in the matrix forms, as shown in Figure 15a–f. First, we can see from Figure 15d–f that the increases of electron density, due to attractive perturbations (δρ(I)/δv(J)<0), are considerably localized in the diagonal region, ${\left\{\delta \rho \left(I\right)/\delta v\left(I\right)\right\}}_{I=1∼141}$. Next, we again confirm from Figure 15a–c that there are distinct sub-blocks in the diagonal region, which correspond to the Mn_4_O_5_Ca core, 9 amino acid residues, and the group of the water molecules. The important point is that the number of off-diagonal parts increases as the threshold becomes tighter from Figure 15a (δρ(I)/δv(J)<0.1) to Figure 15c (δρ(I)/δv(J)<0.001). As described above, the criterion, δρ(I)/δv(J)<0.1, detects the strong couplings such as covalent and ionic bonds: We should note that the sp3 junctions of the residues clearly cut the islands at this level and that the weak ionic bonds such as hydrogen bonds is difficult to detect. In other words, the QM/MM modeling under this criterion is very rough. On the other hand, the model that is constructed according to the guideline, δρ(I)/δv(J)<0.01, the core part is described with the correct surrounding effects such as hydrogen bonds and the hyperconjugation effects. In fact, almost all models that are employed by most QM/MM researchers seem to satisfy this criterion [5,6,7,8,9,10,11,12,13,14,15,16,17,50,51]. As shown Figure 15c, the criterion, δρ(I)/δv(J)<0.001, leads to a greater number of interaction islands, which interconnect among units, and so, in other words, it requires a large QM region. In fact, the extent to which the LRF of density spreads over is shown in Figure S11. If such an accurate QM computational method is available and is needed to describe the electronic structure of the core part of an enzyme, we had better employ this criterion. However, we do not recommend this accurate criterion for actual QM/MM calculations using DFT because, even in the pure QM calculations, the approximated exchange-correlation terms always leads to errors on the description of the density.

#### 3.2.3. Linear Response Functions of Spin Density of OEC

**Figure 16:**
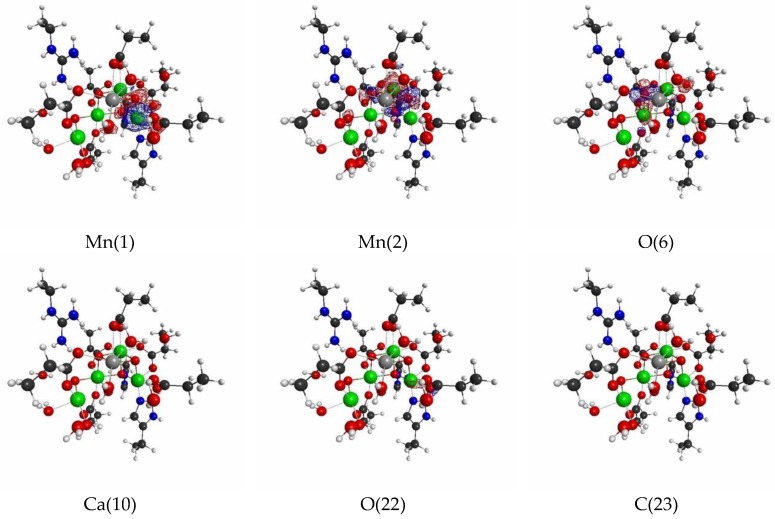
Isosurfaces of linear response functions of spin density for the perturbations on atomic sites of OEC (pdb entry:5b66). The threshold of $\delta \rho_{z}\left(r\right)/\delta v_{m}\left(I\right)$ is ±0.01 (+: blue, −: red). The atomic symbols and those numbers in parentheses below the figures indicate the atoms (I) to which the virtual perturbations are applied.

Next, we show the results of LRFs of spin density for the OEC. In fact, it is known that the ground-state of the dark stable state has anti-ferromagnetic coupling, consisting of two Mn(III) ions and Mn(IV) ions, that are coupled with carboxylate and di-μ-oxo ions bridges, which require the correct treatment of the spin state. Figure 16 shows selected important isosurfaces of LRFs of spin density for the threshold, $\delta \rho_{z}\left(r\right)/\delta v_{m}\left(I\right)=\pm 0.01$. Before discussing the results shown in Figure 16, we should note that almost all LRFs of spin density for the threshold, $\rho_{z}\left(r\right)/\delta v_{m}\left(I\right)=\pm 0.1$, become negligible as shown in Figure S12: Only the perturbations to Mn ions and bridged O ions yield the apparent fluctuations of spin density. As shown in Figure 16 ($\delta \rho_{z}\left(r\right)/\delta v_{m}\left(Mn\left(1\right)\right)$) and $\delta \rho_{z}\left(r\right)/\delta v_{m}\left(Mn\left(2\right)\right)$), the isosurfaces for$\;\delta \rho_{z}\left(r\right)/\delta v_{m}\left(I\right)=\pm 0.01\;$spread over the core and bridged units. Not surprisingly, the perturbations to atoms of water molecules (for instance, O(118), O(124), O(136)) do not emerge spin fluctuations, because there are negligibly spin polarized. However, it is unexpected that, at this threshold, all the isosurfaces for the perturbations to the N(71) and C(74) atoms of His that ligates to Mn(1), and to O(118) and O(121) atoms of H_2_O that ligates to Mn(4), are negligible. Further, we can see from Figure 16a–c that the LRF of spin density for OEC is considerably localized, compared with that of density shown in Figure 13a–c.

**Figure 17:**
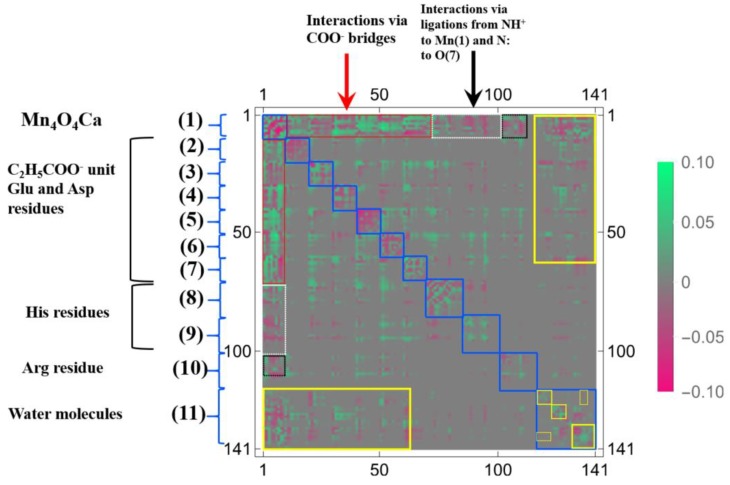
The heat map for the matrix representation of condensed version of linear response functions of spin density,$\;{\left\{\delta \rho_{z}\left(I\right)/\delta v_{m}\left(J\right)\right\}}_{I,J=1∼141}$, for OEC. The number of the axes corresponds to the numbering of the atoms indicated in Figure 11. The functional units and the couplings among units are also indicated (see text for the details).

**Figure 18:**
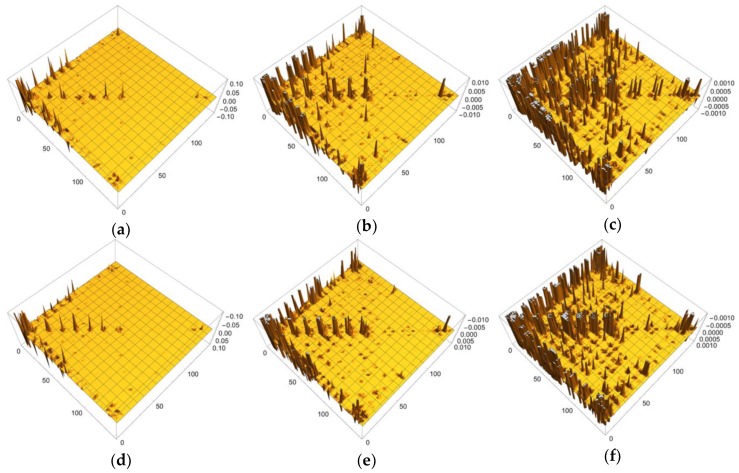
Matrix representation of condensed version of linear response functions of spin density for OEC with the six cutting thresholds. (**a**) $\delta \rho_{z}\left(I\right)/\delta v_{m}\left(J\right)<0.1.$ (**b**) $\delta \rho_{z}\left(I\right)/\delta v_{m}\left(J\right)<0.01$. (**c**) $\delta \rho_{z}\left(I\right)/\delta v_{m}\left(J\right)<0.001$, (**d**) $\delta \rho_{z}\left(I\right)/\delta v_{m}\left(J\right)>-0.1.$ (**e**) $\delta \rho_{z}\left(I\right)/\delta v_{m}\left(J\right)>-0.01$. (**f**) $\delta \rho_{z}\left(I\right)/\delta v_{m}\left(J\right)>-0.001$.

All other non-negligible isosurfaces for $\delta \rho_{z}\left(r\right)/\delta v_{m}\left(I\right)=\pm 0.01$ and $\delta \rho_{z}\left(r\right)/\delta v_{m}\left(I\right)=\pm 0.001$ are shown in Figures S13 and S14, respectively. As an overview, we plot a heat-map representation of the matrix, ${\left\{\delta \rho_{z}\left(I\right)/\delta v_{m}\left(J\right)\right\}}_{I,J=1∼141}$ in Figure 17. In addition, we plot the sections for the six thresholds in Figure 18a–f. We can see that the gray region in the heap-map for ${\left\{\delta \rho_{z}\left(I\right)/\delta v_{m}\left(J\right)\right\}}_{I,J=1∼141}$ shown in Figure 17 is larger than that in the heat-map for ${\left\{\delta \rho \left(I\right)/\delta v\left(J\right)\right\}}_{I,J=1∼141}$ shown in Figure 14. Since the gray region corresponds to the region where there is nearly no response, i.e., $\delta \rho_{z}\left(I\right)/\delta v_{m}\left(J\right)≅0.0$, the almost all region of the OEC model is not sensitive to the virtual magnetic perturbations. Also as shown in Figure 18a,d, the sections do not exist for both $\delta \rho_{z}\left(I\right)/\delta v_{m}\left(J\right)<0.1\;$and $\delta \rho_{z}\left(I\right)/\delta v_{m}\left(J\right)>-0.1$, being consistent with the above-mentioned fact that almost all LRFs of spin density for the threshold, $\rho_{z}\left(r\right)/\delta v_{m}\left(I\right)=\pm 0.1$, become negligible, as shown in Figure S11. However as the threshold becomes tighter, the number of sections increase, as shown in Figure 18b,c,e,f. These results are consistent with the fact that the isosurfaces for$\;\delta \rho_{z}\left(r\right)/\delta v_{m}\left(I\right)=\pm 0.01\;$spread over the Mn ions in core and bridged carboxylate units as described above (see figures $\delta \rho_{z}\left(r\right)/\delta v_{m}\left(I\right)=\pm 0.01,$ I=Mn(1), Mn(2), O(6),O(22),C(23) in Figure 16). Furthermore, as shown in Figure 18, for the threshold,$\;\delta \rho_{z}\left(I\right)/\delta v_{m}\left(J\right)<0.001\;$and $\delta \rho_{z}\left(I\right)/\delta v_{m}\left(J\right)>-0.001$, the sections appear in the off-diagonal regions, (i) between the Mn_4_O_5_Ca core and the His residue, (ii) the Mn_4_O_5_Ca core and the Arg residue, and (iii) Mn_4_O_5_Ca core and the group of water molecules. These interactions, together with the intra residue and the intra water cluster interactions are shown as dull green and dull red in Figure 17.

If we consider the threshold, $\delta \rho_{z}\left(r\right)/\delta v_{m}\left(I\right)=\pm 0.01$ is appropriate for the guideline to construct the QM/MM model; the small QM model consist of the Mn_4_O_5_Ca core and the carboxylates, that ligate to the Mn_4_O_5_Ca core enough to describe the magnetic systems embedded in non-magnetic environments such as proteins and waters. One of the theoretical reasons in raising such a question, is that the degree of freedom of charges basically does not couple with that of the spins. As most of the biological environments are affected electrostatically, and not magnetically, a large QM region does not always improve the descriptions of the magnetic systems of the active site. In fact, ab initio DFT calculations showed that the counter anions in molecular solids, including di-nuclear Mn complexes do not affect the intra-molecular magnetic interactions of the Mn complexes considerably [53,54,55]. However, we should note that our guidelines given by Equation (17) are not only of the spin density, but also of the density. In addition, we should note that the energy resolution to correctly estimate the relative stability of degenerate states for the Mn complexes, such as OEC, is less than that of the so-called “chemical accuracy”(1 kcal/mol). Because the magnitude of the magnetic interactions between the Mn ions bridged with carboxylate and di-μ-oxo ions are usually in the order of 100 cm^−1^ or less (<0.3 kcal/mol)[53], which is less than one-tenth of the binding energy of a usual hydrogen bond. Thus in order to determine the spin state of the ground state among many degenerate states, we usually need a far more accurate method to describe the electrostatic interactions correctly. Therefore small electrostatic interactions might affect the relative stabilities among degenerate spin states, due to the higher order effects on the electronic structure of the active site. This implies that the criterion of the LRF of spin density must not be $\rho_{z}\left(r\right)/\delta v_{m}\left(I\right)=\pm 0.01$, but $\delta \rho_{z}\left(r\right)/\delta v_{m}\left(I\right)=\pm 0.001$ or tighter criterions for describing nearly degenerate spin states. However, we would like to emphasize again that, for estimating the correct stable spin states, the problem remains rather in the approximation of the exchange-correlation functional we used than how to model the system by the QM/MM method [53,55]. In the next section, we examine the specific spin state and oxidation state that were proposed to be as the ground state of the OEC core [50,51] to evade the issue, such as the performance of the XC functional for estimating the relative stability among many degenerate states. In other words, we focus our attention only on the errors of densities and spin densities from the full QM model, which is given by Figure 11 in this study.

#### 3.2.4. QM Cluster and QM/MM Calculations for Several Models of OEC

**Figure 19:**
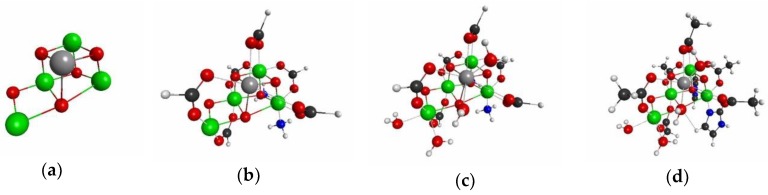
Geometries of the three models for the active site of OEC: (**a**) model 1, (**b**) model 2, (**c**) model 3, (**d**) model 4. We assumed that the full model is the model shown in Figure 11.

We now show the actual QM/MM calculation results for several models shown in Figure 19. Here we assumed that the reaction center (RC) of OEC is the Mn_4_O_5_Ca core, which is considered as model 1 shown in Figure 19a. In model 2, presented in Figure 19b, we approximate all the residues of glutamic acids and aspartic acids residues as formic acids, the His residue as NH_3_, and the protonated His residue as NH_4_^+^. Model 3 further includes water molecules that ligate to the Ca ion or the Mn ion in the Mn_4_O_5_Ca core, as shown in Figure 19c, resulting in the coordination saturation model. Figure 19d shows Model 4, which suits the criteria |δρ(I)/δv(RC)|<0.01 and $\left|\delta \rho_{z}^{}\left(I\right)/\delta v_{m}\left(\mathrm{RC}\right)\right|<0.01$. In all of the models, we replaced the original parts in the full model by capped H atoms. We computed both the QM cluster model and the QM/MM model with the link atom method for the models 1–4 [3,4,5]. The details of the calculations are described in the Materials and Methods section.

Then, we listed the errors of densities and spin densities of the Mn_4_O_5_Ca core in Table 1, and Table 2, respectively. For complete data of the errors on densities and spin densities, please refer to Tables S3 and S4, respectively. We can see from tables Table 3 Table 4 that the errors due to the simplest model (Model 1) are too large to discuss the oxidation state and spin state: The maximum errors are 1.158 (Ca(10)) for densities and −0.858 (Mn(2)) for the spin densities. Such large errors are not improved by the QM/MM model for Model 1. Introducing all the carboxylates that bridge among the Mn ions and the Ca ion improve the results considerably, in particular—the spin densities. The errors of spin densities and electron densities reduce to, at most ±0.2, and ±0.4, respectively. In Model 2, the effective point charges of the surrounding atoms do not improve the accuracy of the QM cluster model. The acceptable accuracy is obtained with Model3, of which the magnitudes of all errors reduce to less than 0.1. Furthermore, Model 4 yields quite accurate results compared with the full model. In addition, the QM/MM model based on Model 4 out-perform the QM cluster model based on Model 4. In particular, almost all of the errors of $\Delta \rho_{z}^{}$ of the Mn_4_O_5_Ca core are less than 0.01, implying that the QM/MM model based on Model4 is nearly equivalent to the full QM model. We can also see from Table S3 that for Model 4, that artificial polarizations occur on the peripheral atoms (see the densities of the terminal carbons of amino residues, {C(x)|, x = 14,243,444, 54,647,490} and the terminal hydrogen atoms of water molecules {H(x)|x = 120,126,128,129}); the atoms in the inner region are nearly completely protected from the artificial electric fields and the edge effects (the QM cluster) because of the screening effects due the electrons in the outer regions. Since Model 4 is the model that satisfies our guidelines given by Equation (17), this result exemplifies the validity of our method to determine the QM region in the QM/MM calculation for the reaction center embedded in proteins.

## 4. Materials and Methods

### 4.1. Numerical Details for Computations of “Condensed” Versions of the Linear Response Function

Here we describe the numerical details to compute condensed versions of the linear response function (LRF). The purpose is to obtain the density change at r due to the perturbation that is applied to the J-th atom, δρ(r)/δv(J),

(18) $$\frac{\delta \rho \left(r\right)}{\delta v\left(J\right)}=\int_{J-th\;atom}^{}dr\prime \frac{\delta \rho \left(r\right)}{\delta v\left(r\prime \right)}$$

and the density change at the I-th atom due to the perturbation that is applied to the J-th atom, δρ(I)/δv(J),

(19) $$\frac{\delta \rho \left(I\right)}{\delta v\left(J\right)}=\int_{I-th\;atom}^{}dr\int_{J-th\;atom}^{}dr\prime \frac{\delta \rho \left(r\right)}{\delta v\left(r\prime \right)}.$$

Here the point is the numerical integration over all space {r}, and {r′}. We employ the Wigner-Seitz cell for the domain of integration for the I-th (and J-th) atom, where the cell for a specific atom in the molecule can be defined as a region encircled by all perpendicular bisectors with neighboring atoms. Then the 50 point Euler-Maclaurin quadrature and the 302 point Lebedev quadrature are used for radial, and spherical integrations [56,57], respectively. This is a similar quadrature scheme to that used in Kohn-Sham DFT code used in quantum chemistry program [58], but the fuzzy cell scheme is not used. The above numerical scheme is also used for the LRF of spin density.

### 4.2. Computational Details of Transition Metal Complexes

We used the B3LYP functional for the exchange-correlation (XC) function for all spin-unrestricted DFT calculations. The basis sets used in the computations are follows. The Wachter+f basis is used for Fe and Mn for all transition metal complexes [59,60]. The 6-31G** basis are used for other atoms of P450. For C, N, O, H atoms of reaction center of PS II, 6-31G* basis is used. The missing protons in pdb files are added and the partial optimization calculations are performed using similar XC and basis sets with fixing the positions of all heavy atoms. In order to facilitate the convergences of self-consistent and geometry optimization partial procedures, we used Gaussian 09 Rev. C first [60,61], and then using molecular orbitals obtained after diagonalization of the Kohn-Sham Hamiltonian constructed from the converged molecular orbitals. We performed linear response computations by using the modified version of GAMESS [41]. We employed MacMolPlt [62] to visualize the equi-valued surfaces of LRF, and Mathematica Ver. 11 [63] for visualizing the matrix form of LRFs, respectively. For the QM/MM calculations described in Section 3.1.4 and Section 3.2.4, we employed the standard approach of the QM/MM method with the link-atom method [3,4,5]: i.e., we approximated the MM parts except the frontier MM atoms by the point charges that are determined by the electro-static potential fitting (ESP) method [64]. The positions of the capped H atoms are partially optimized. For the computation of the partially optimization of the capped H atoms and the computation of the ESP charges of the MM parts of P450 and OEC models, we used Gaussian 09 Rev. C [60].

## 5. Conclusions

We computed the linear response functions (LRFs) of mono- and poly-nuclear transition metal complexes, which have open-shell transition metal ions. From our computations, it is concluded that the order of the LRF values for the virtual perturbations, that are applied to the surrounding atoms, i.e., δρ(r)/δv(J) and $\delta \rho_{z}^{}\left(r\right)/\delta v_{m}\left(J\right)$, can be used to determine the approximation level of the QM/MM modeling for such systems. The modeling that satisfies $\left|\delta \rho \left(r\right)/\delta v\left(J\right)\right|\;\left(and\;\left|\delta \rho_{z}^{}\left(r\right)/\delta v_{m}\left(J\right)\right|\right)<0.1$ is a very rough model. The model covers the environments that are strongly coupled with the active spot, but weak couplings, due to hydrogen bonding and hyper-conjugations are missed. The residues that directly ligate to the active site should be included in the model. Even the partitioning of the poly-nuclear transition metal cores is often possible if we employed this criterion. This was often used for biochemical systems until the 1990s [52]. If the model satisfies the criterion, $\left|\delta \rho \left(r\right)/\delta v\left(J\right)\right|\;\left(and\;\left|\delta \rho_{z}^{}\left(r\right)/\delta v_{m}\left(J\right)\right|\right)<0.01$, it is consistent with almost all QM/MM modeling [5,50,51]. In order to satisfy the guidelines given by Equation (17), i.e., |δρ(r)/δv(J)| <0.01 and $\left|\delta \rho_{z}^{}\left(r\right)/\delta v_{m}\left(J\right)\right|<0.01$, the next surrounding environments beyond the weakly coupled interconnections, such as hydrogen bonds and hyperconjugations over sp3 junction, should be included in the QM region. In fact, our QM cluster and QM/MM models, based on these guidelines, yield accurate densities and spin densities of the reaction centers of P450 and OEC (see Section 3.1.4 and Section 3.2.4), compared with the full QM model.

Of course, the use of δρ(r)/δv(J) and $\delta \rho_{z}^{}\left(r\right)/\delta v_{m}\left(J\right)$ implies that the above suggestions would become meaningless if we developed an accurate methods to reduce δv and $\delta v_{m}$ to nearly zero: What we should treat with the QM approach would only be the actual spot that is responsible for the QM phenomenon. However, it would be very difficult to develop such a “nearly exact” approximation, by which the environmental effects, except the active spot, can be re-normalized in some effective fields. In addition, before we proceed to such an accurate level, we need to compare the errors due to QM/MM modeling with those resulting from the choice of the XC functional or the basis set that we used.

Comparing the LRF of density and the LRF of spin density, the conclusion is that the density of the active site is more sensitive than the spin density of the active site for the effects from the perturbations of surrounding environments. However, this does not imply that the small QM model is enough for an accurate description of the transition metal complexes, having open-shell transition metal ions embedded in proteins. To obtain accurate electron densities and spin densities for such systems, the model is required to satisfy not only the condition for electron densities, but also that for spin densities. In fact, our QM cluster and QM/MM calculations showed that the models which satisfy both |δρ(r)/δv(J)| <0.01 and $\left|\delta \rho_{z}^{}\left(r\right)/\delta v_{m}\left(J\right)\right|<0.01$ yields accurate results that are nearly equivalent to those of the full QM model. Furthermore, in order to determine the spin-state of the ground-state correctly, more accurate computations would be needed. This would require both a large QM region to satisfy the criterion, $\left|\delta \rho_{z}^{}\left(r\right)/\delta v_{m}\left(J\right)\right|<0.001$, and an accurate QM method.

## Figures and Tables

**Table 1:** The errors of the calculated values, obtained by the quantum mechanics (QM) cluster and the quantum mechanics/molecular mechanics (QM/MM) models and the reference values on densities (Δρ) of the Fe ion, the N atoms of the porphyrin ring, and the S ion of the Cys residue. The reference values are those of the full model shown in Figure 1.

Atom	Model 1		Model 2		Model 3	
	QM	QM/MM	QM	QM/MM	QM	QM/MM
Fe	0.069	0.099	−0.006	−0.013	0.001	0.014
N	0.025	0.034	0.001	0.001	0.000	−0.001
N	0.020	0.029	0.000	0.000	0.000	0.000
N	0.017	0.028	0.000	0.001	0.000	−0.001
N	0.020	0.027	0.004	0.003	0.000	−0.003
S	0.082	-0.018	−0.006	0.033	−0.001	−0.015

**Table 2:** The errors of the calculated values obtained by the QM cluster and the QM/MM models and the reference values on spin densities ($\Delta \rho_{z}^{}$) of the Fe ion, the N atoms of the porphyrin ring, and the S ion of the Cys residue. The reference values are those of the full model shown in Figure 1.

Atom	Model 1		Model 2		Model 3	
	QM	QM/MM	QM	QM/MM	QM	QM/MM
Fe	0.023	−0.070	0.007	0.017	−0.002	−0.016
N	0.015	0.002	0.002	0.005	−0.001	−0.004
N	0.018	0.004	0.000	0.002	0.000	−0.002
N	0.013	−0.002	0.000	0.001	0.000	−0.001
N	0.025	0.014	−0.001	0.000	0.000	−0.001
S	−0.113	0.024	−0.018	−0.039	0.003	0.035

**Table 3:** The errors of the calculated values obtained by the QM cluster and the QM/MM models from the reference values on densities (Δρ) of the Mn_4_O_5_Ca core. The reference values are those of the full model shown in Figure 11.

Atom	Model 1		Model 2		Model 3		Model 4	
	QM	QM/MM	QM	QM/MM	QM	QM/MM	QM	QM/MM
Mn(1)	0.941	1.069	0.051	0.032	0.041	0.025	0.004	0.005
Mn(2)	0.835	1.050	0.036	0.026	0.030	0.021	0.017	0.014
Mn(3)	0.571	0.687	0.041	0.013	0.024	−0.001	0.019	0.001
Mn(4)	1.071	1.134	0.237	0.365	0.026	0.043	0.009	0.024
O(5)	0.185	0.192	−0.003	0.024	−0.001	0.025	−0.013	0.005
O(6)	0.230	0.158	0.036	−0.006	0.042	−0.003	0.037	−0.014
O(7)	0.236	0.033	−0.015	−0.034	−0.019	−0.036	−0.008	−0.007
O(8)	0.380	0.178	0.071	0.021	0.021	−0.002	0.011	−0.016
O(9)	0.224	0.153	−0.004	0.002	−0.009	0.002	−0.011	0.004
Ca(10)	1.158	1.175	0.364	0.412	0.022	0.004	0.014	−0.005

**Table 4:** The errors of the calculated values obtained by the QM cluster and the QM/MM models from the reference values on densities ($\Delta \rho_{z}^{}$) of the Mn_4_O_5_Ca core. The reference values are those of the full model shown in Figure 11.

Atom	Model 1		Model 2		Model 3		Model 4	
	QM	QM/MM	QM	QM/MM	QM	QM/MM	QM	QM/MM
Mn(1)	0.321	0.305	−0.012	−0.015	−0.012	−0.015	−0.003	−0.002
Mn(2)	−0.858	−0.634	−0.012	−0.026	−0.026	−0.033	−0.020	−0.004
Mn(3)	0.267	0.272	−0.010	0.027	−0.027	0.015	−0.033	0.008
Mn(4)	−0.630	−0.507	−0.162	−0.141	−0.007	−0.001	−0.006	0.000
O(5)	0.112	0.148	−0.005	0.004	−0.002	0.005	0.000	0.002
O(6)	0.224	0.157	0.012	0.003	0.033	0.014	0.031	0.004
O(7)	0.167	0.116	−0.017	−0.015	−0.008	−0.011	−0.004	−0.001
O(8)	0.627	0.306	0.092	0.036	0.019	−0.006	0.021	0.001
O(9)	−0.111	−0.048	−0.040	−0.060	0.021	−0.016	0.026	−0.011
Ca(10)	−0.003	−0.001	0.002	0.000	0.001	0.000	0.001	0.000

## Supplementary Materials

The following are available online, Figures S1–S14; Tables S1–S4.
