# Supplementary material for: Linear Response Functions of Densities and Spin Densities for Systematic Modeling of the QM/MM Approach for Mono- and Poly-Nuclear Transition Metal Systems
Source: Molecules. 2019 Feb 25;24(4):821. doi: 10.3390/molecules24040821 (PMC6412466; doi:10.3390/molecules24040821)
Supplement: Supplementary file 1 [file molecules-24-00821-s001.pdf]

Linear Response Functions of Densities and Spin Densities for Systematic Modeling of the QM/MM Approach for Mono- and Poly-Nuclear Transition Metal Systems

Colin K. Kitakawa [1], Tomohiro Maruyama [1], Jinta Oonari [1], Yuki Mitsuta [1], Takashi Kawakami [1], Mitsutaka Okumura [1], Kizashi Yamaguchi [2] and Shusuke Yamanaka [1,3,*]

[1] Graduate School of Science, Osaka University, Osaka 565-0871, Japan; kitakawac15@chem.sci.osaka-u.ac.jp (C.K.K.), maruyamat16@chem.sci.osaka-u.ac.jp (T.M.), oonarij16@chem.sci.osaka-u.ac.jp (J.O.), mitsutay13@chem.sci.osaka-u.ac.jp (Y.M.), kawakami@chem.sci.osaka-u.ac.jp (T.K.), ok@chem.sci.osaka-u.ac.jp (M.O.)
[2] Institute for Nanodesign, Osaka University, Osaka 560-8531, Japan, yama@chem.sci.osaka-u.ac.jp
[3] Quantum information, Quantum Biology division, Institute for Open and Transdisciplinary Research Initiatives, Osaka University, Osaka 560-8531, Japan
* Correspondence: syama@chem.sci.osaka-u.ac.jp; Tel.:+81-6-6850-5406

Supplementary Materials:

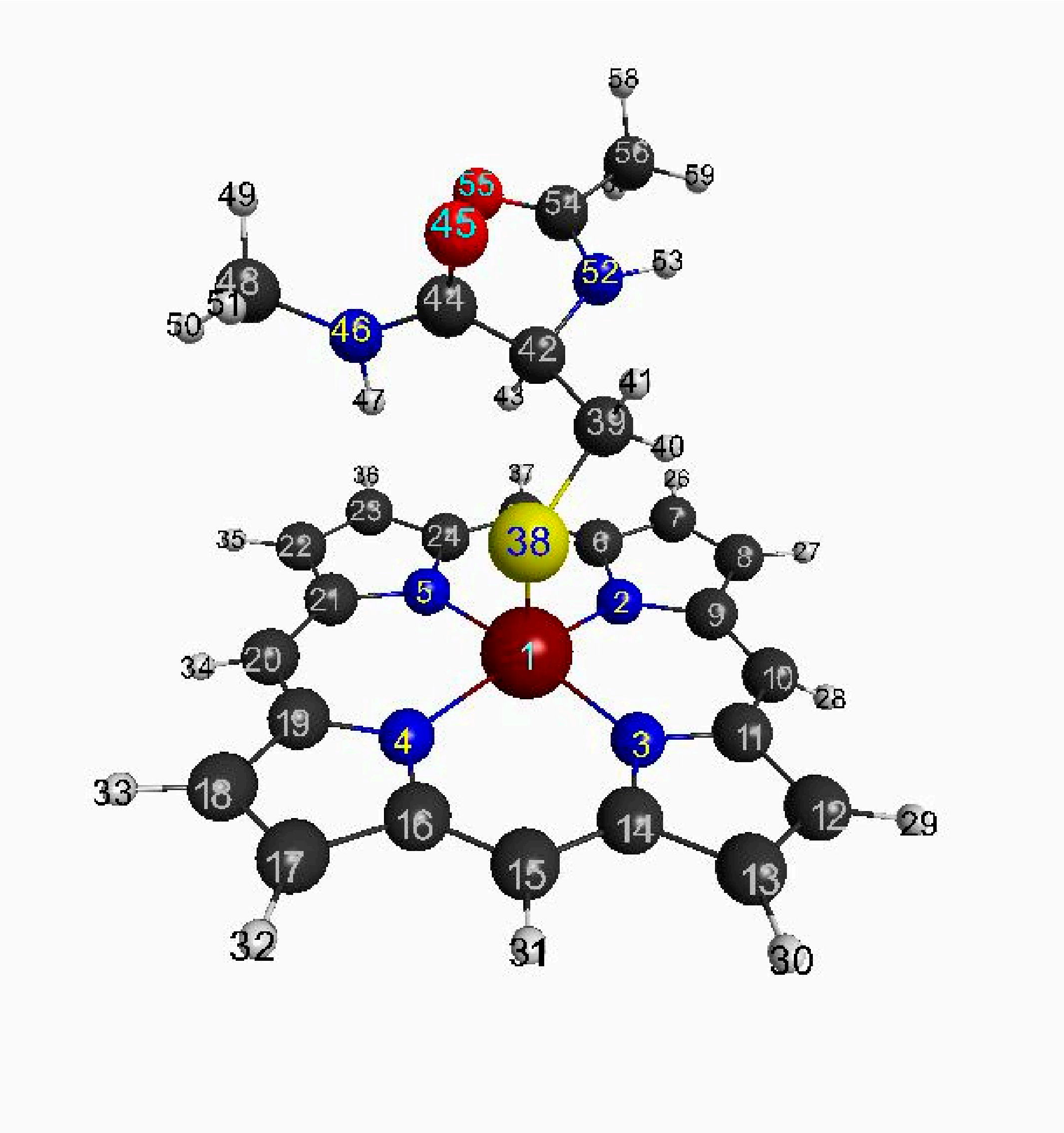

Figure S-1. Geometry of the active site of P450 (pdb entry:5vws). The numbering of the atoms is used for the following figures to show linear response functions. This figure is the same as figure 1 , but the size is much larger than figure 1.

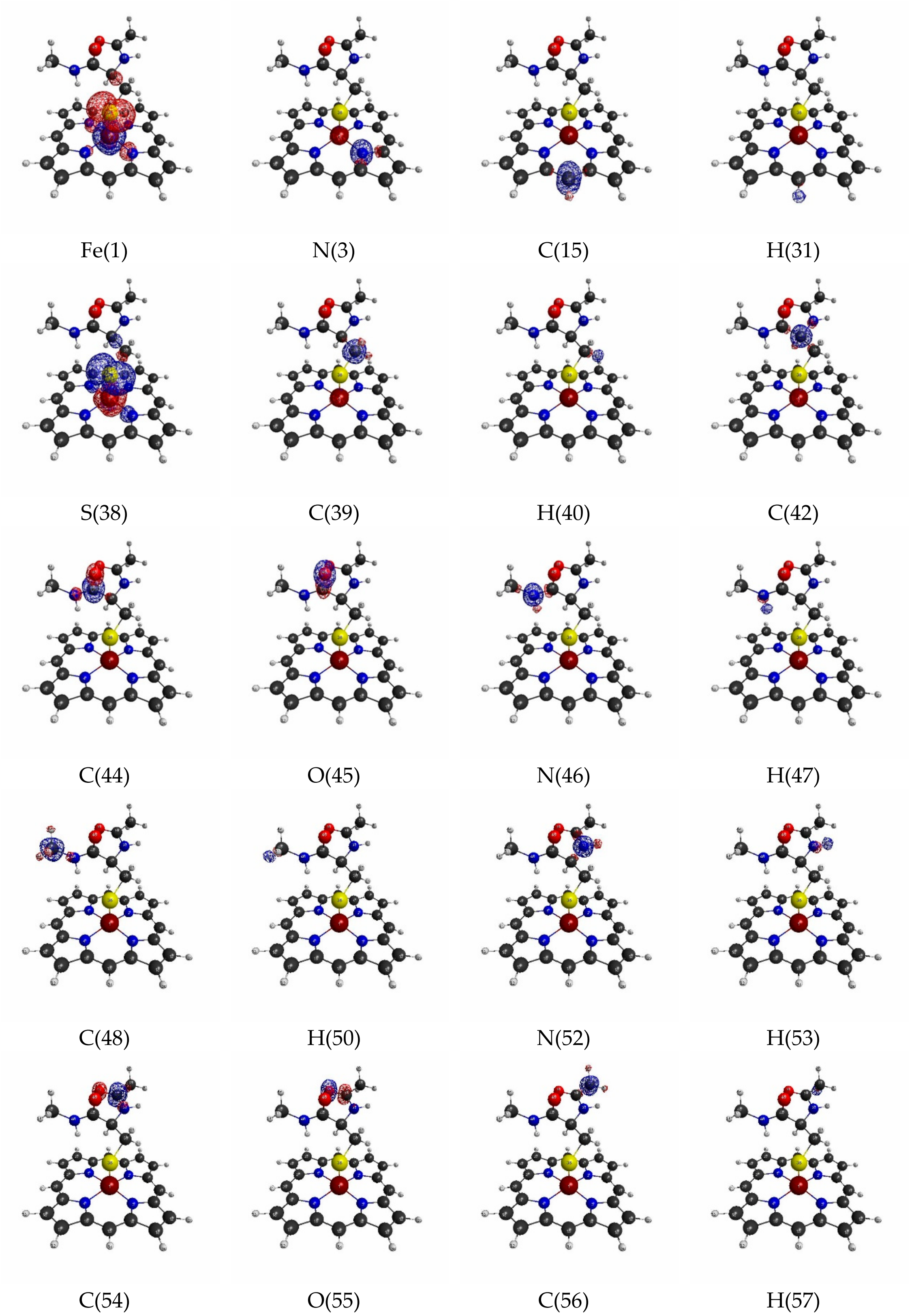

Figure S-2. Isosurfaces of linear response functions of density for the perturbations on atomic sites of P450 (pdb entry:5vws). The threshold of $\delta\rho(\boldsymbol{r})/\delta v(I)$ are ± 0.1 (+: blue, −: red). The atomic symbols and those numbers in parentheses below the figures indicate the atoms (I) to which the virtual perturbations are applied.

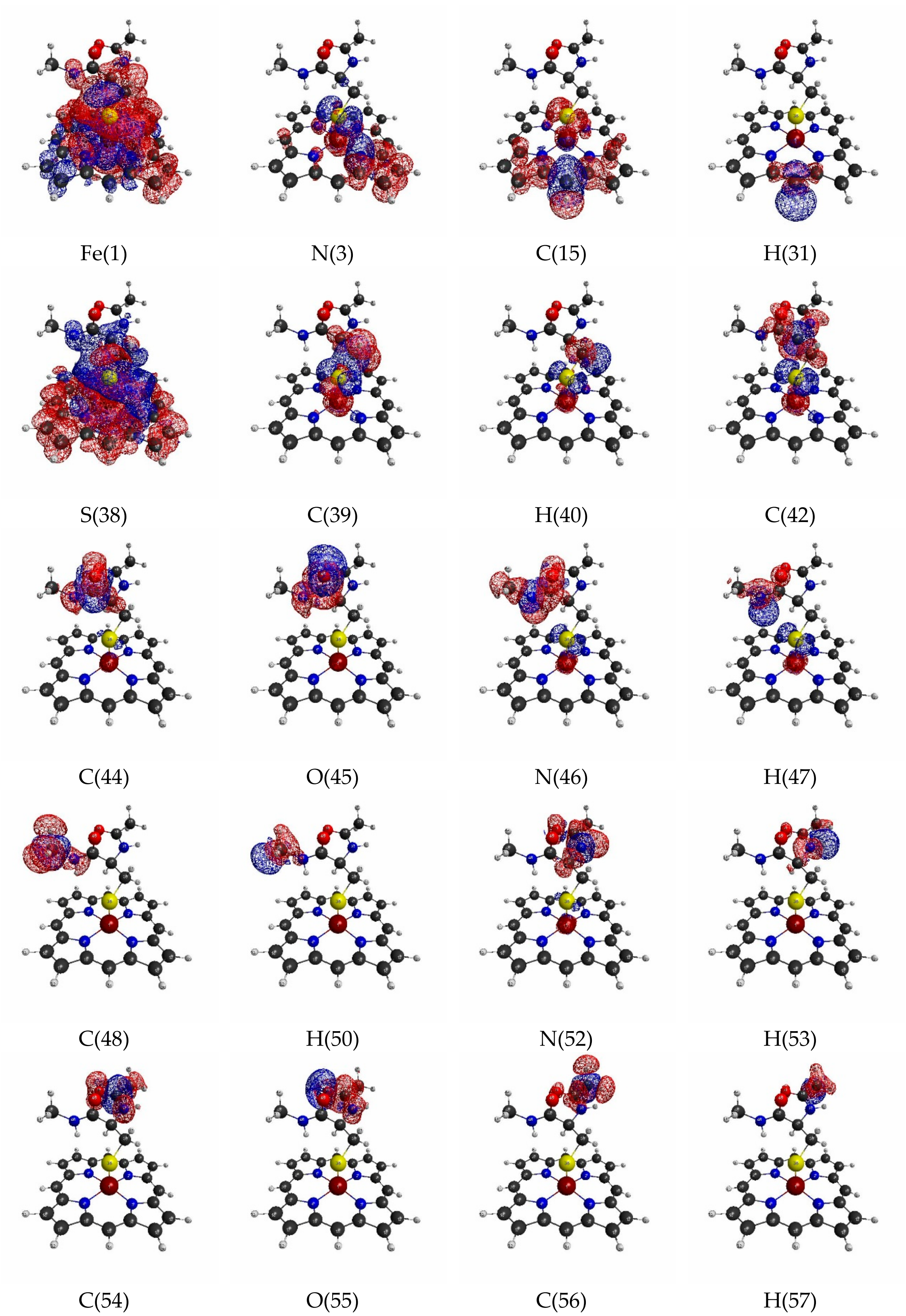

Figure S-3. Isosurfaces of linear response functions of density for I-the perturbations on atomic sites of P450 (pdb entry:5vws). The threshold of $\delta\rho(\boldsymbol{r})/\delta v(I)$ are ± 0.01 (+: blue, −: red). The atomic symbols and those numbers in parentheses below the figures indicate the atoms (I) to which the virtual perturbations are applied.

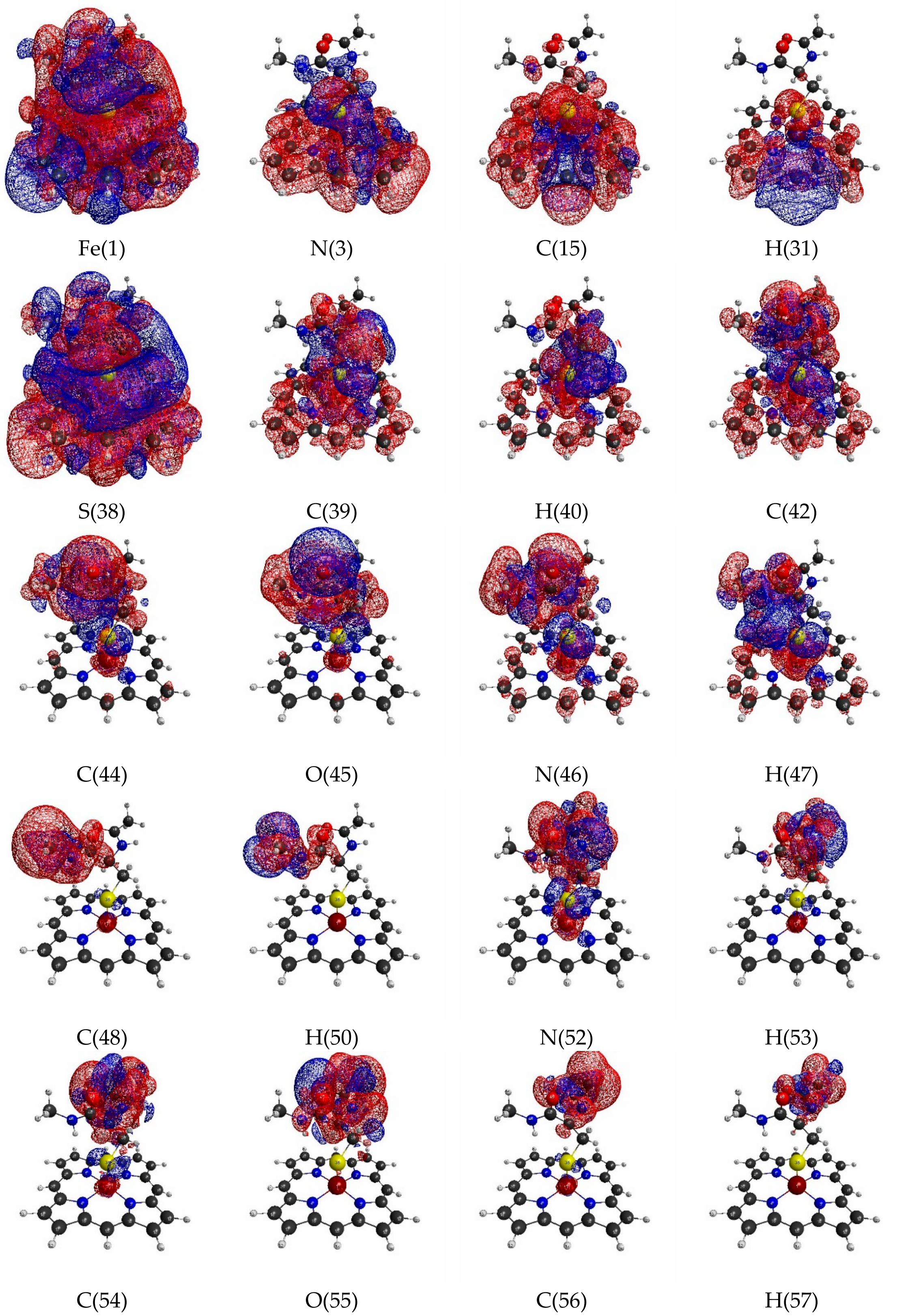

Figure S-4. Isosurfaces of linear response functions of density for the perturbations on atomic sites of P450 (pdb entry:5vws). The threshold of $\delta\rho(\boldsymbol{r})/\delta v(I)$ are ± 0.001 (+: blue, −: red). The atomic symbols and those numbers in parentheses below the figures indicate the atoms (I) to which the virtual perturbations are applied.

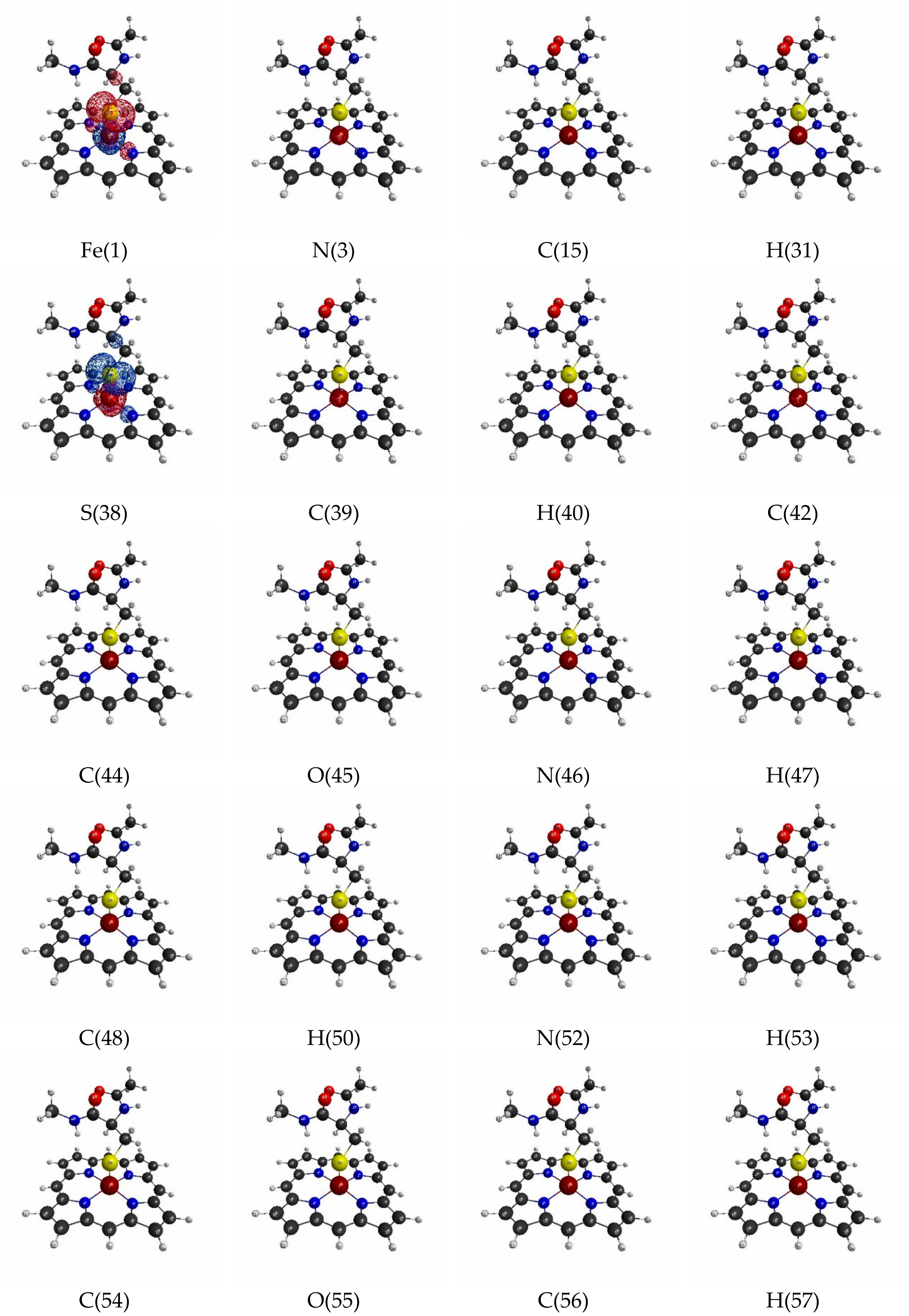

Figure S-5. Isosurfaces of linear response functions of density for the perturbations on atomic sites of P450 (pdb entry:5vws). The threshold of $\delta\rho_z(\boldsymbol{r})/\delta v_m(I)$ are ± 0.1 (+: blue, −: red). The atomic symbols and those numbers in parentheses below the figures indicate the atoms (I) to which the virtual perturbations are applied.

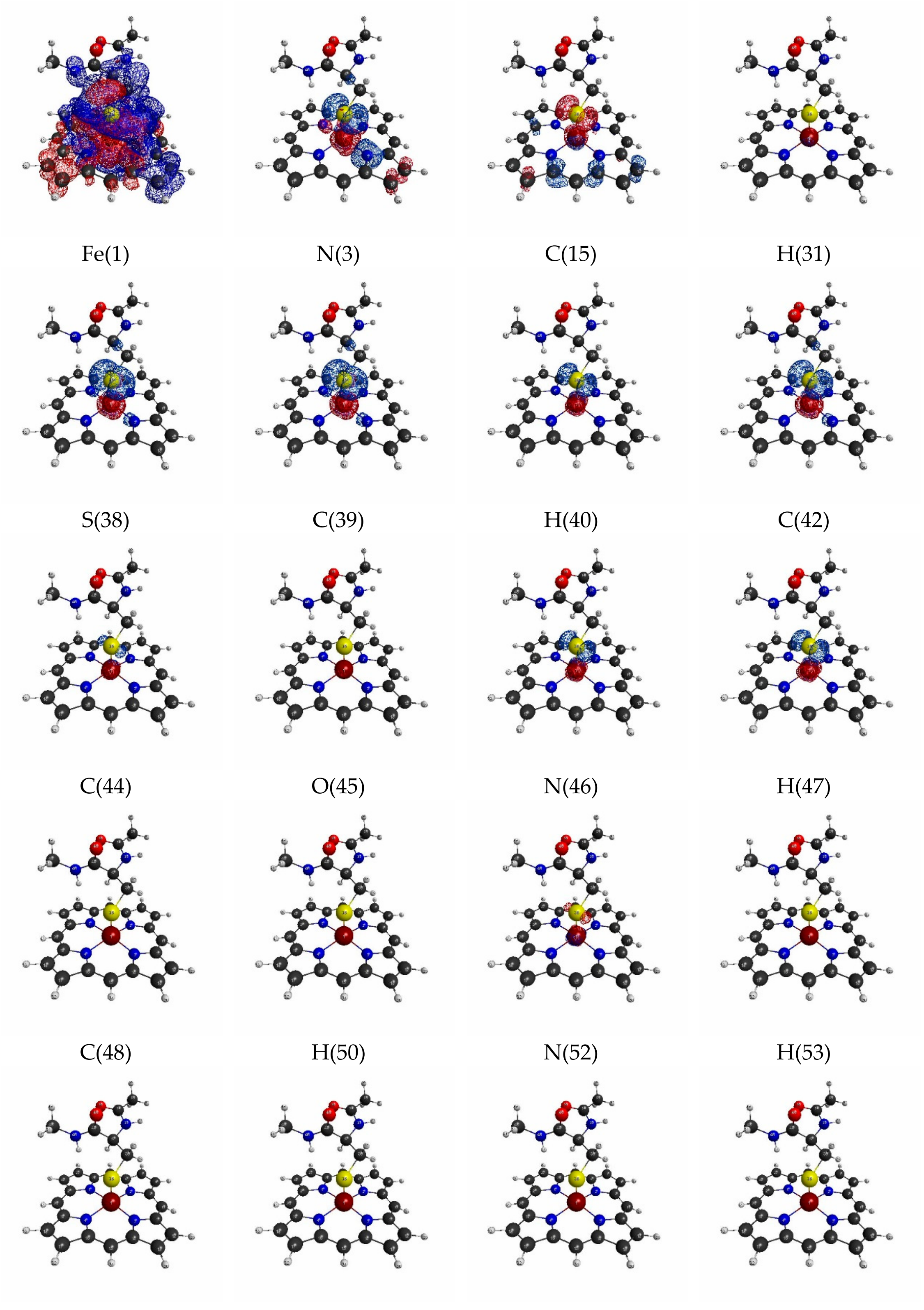

Figure S-6. Isosurfaces of linear response functions of density for the perturbations on atomic sites of P450 (pdb entry:5vws). The threshold of $\delta\rho_z(\boldsymbol{r})/\delta v_m(I)$ are ± 0.01 (+: blue, −: red). The atomic symbols and those numbers in parentheses below the figures indicate the atoms (I) to which the virtual perturbations are applied.

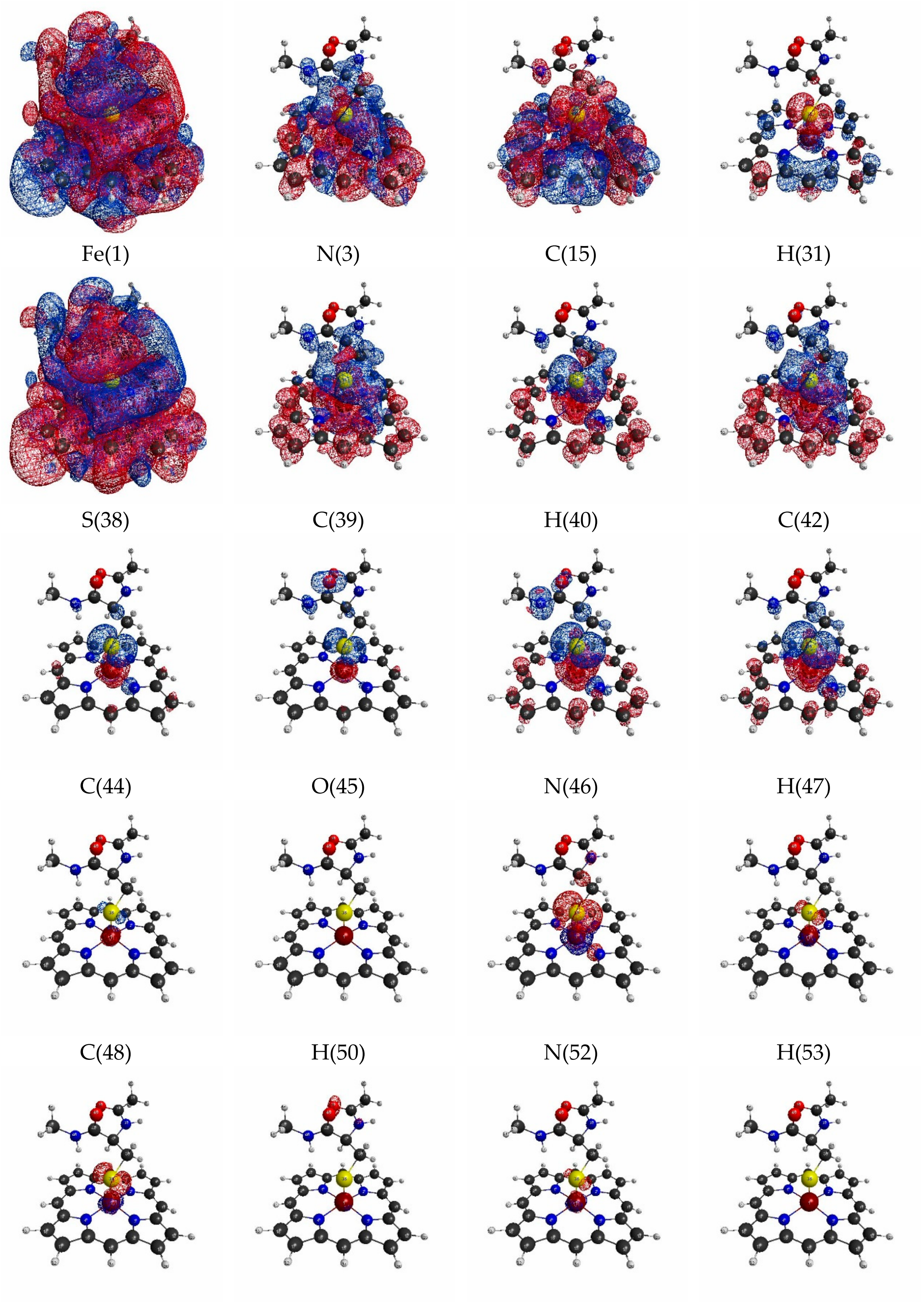

Figure S-7. Isosurfaces of linear response functions of density for the perturbations on atomic sites of P450 (pdb entry:5vws). The threshold of $\delta\rho_z(\boldsymbol{r})/\delta v_m(I)$ are ± 0.001 (+: blue, −: red). The atomic symbols and those numbers in parentheses below the figures indicate the atoms (I) to which the virtual perturbations are applied.

Table S-1. The errors of the calculated values obtained by the QM cluster and the QM/MM models from the reference values on densities ($\Delta\rho$) of P450. The reference values are those of the full model shown in figure 1.

	Model 1		Model 2		Model 3	
	QM	QM/MM	QM	QM/MM	QM	QM/MM
Fe(1)	0.069	0.099	-0.006	-0.013	0.001	0.014
N(2)	0.025	0.034	0.001	0.001	0.000	-0.001
N(3)	0.020	0.029	0.000	0.000	0.000	0.000
N(4)	0.017	0.028	0.000	0.001	0.000	-0.001
N(5)	0.020	0.027	0.004	0.003	0.000	-0.003
C(6)			0.001	-0.002	0.000	-0.003
C(7)			0.000	-0.008	0.000	-0.001
C(8)			-0.001	-0.007	0.000	0.003
C(9)			-0.001	-0.003	0.000	0.002
C(10)			-0.002	-0.005	0.000	0.005
C(11)			-0.002	-0.003	0.000	0.003
C(12)			-0.003	-0.006	0.000	0.007
C(13)			-0.003	-0.005	0.000	0.007
C(14)			-0.002	-0.003	0.000	0.004
C(15)			-0.003	-0.004	0.000	0.006
C(16)			-0.003	-0.002	0.000	0.003
C(17)			-0.003	-0.004	0.000	0.006
C(18)			-0.003	-0.002	0.000	0.003
C(19)			-0.002	0.001	0.000	0.000
C(20)			-0.001	0.002	0.000	-0.003
C(21)			0.003	0.004	0.000	-0.004
C(22)			-0.001	0.000	0.000	-0.002
C(23)			-0.004	-0.001	0.000	-0.002
C(24)			0.004	0.003	0.000	-0.004
C(25)			0.000	-0.001	0.000	-0.004
H(26)			-0.001	-0.006	0.000	0.000
H(27)			-0.001	-0.005	0.000	0.004
H(28)			-0.001	-0.004	0.000	0.004
H(29)			-0.002	-0.004	0.000	0.005
H(30)			-0.002	-0.004	0.000	0.006
H(31)			-0.002	-0.003	0.000	0.004
H(32)			-0.002	-0.004	0.000	0.005
H(33)			-0.002	-0.002	0.000	0.003
H(34)			-0.001	-0.001	0.000	0.002
H(35)			-0.002	-0.002	0.000	0.002
H(36)			-0.005	-0.003	0.000	0.001

H(37)			-0.002	-0.003	0.000	-0.002
S(38)	0.082	-0.018	-0.006	0.033	-0.001	-0.015
C(39)	0.000	-0.033	-0.002	0.002	0.000	0.000
H(40)	0.025	0.016	0.000	-0.016	0.001	0.004
H(41)	0.042	-0.006	-0.008	0.006	0.001	0.002
C(42)			-0.147	-0.123	0.002	0.005
H(43)			-0.002	-0.008	0.001	0.006
C(44)					0.001	0.010
O(45)					-0.006	0.011
N(46)					-0.048	-0.052
H(47)					0.001	0.008
N(52)					0.005	0.018
H(53)					0.013	0.009
C(54)					-0.033	-0.042
O(55)					0.017	0.038

Table S-2. The errors of the calculated values obtained by the QM cluster and the QM/MM models from the reference values on densities ($\Delta\rho_z$) of P450. The reference values are those of the full model shown in figure 1.

	Model 1		Model 2		Model 3	
	QM	QM/MM	QM	QM/MM	QM	QM/MM
Fe(1)	0.023	-0.070	0.007	0.017	-0.002	-0.016
N(2)	0.015	0.002	0.002	0.005	-0.001	-0.004
N(3)	0.018	0.004	0.000	0.002	0.000	-0.002
N(4)	0.013	-0.002	0.000	0.001	0.000	-0.001
N(5)	0.025	0.014	-0.001	0.000	0.000	-0.001
C(6)			0.000	0.001	0.000	-0.001
C(7)			0.000	0.001	0.000	-0.001
C(8)			0.000	0.001	0.000	-0.001
C(9)			0.000	0.000	0.000	0.000
C(10)			0.000	0.001	0.000	-0.001
C(11)			0.000	0.000	0.000	-0.001
C(12)			0.000	0.001	0.000	-0.001
C(13)			0.000	0.000	0.000	-0.001
C(14)			0.000	0.001	0.000	-0.001
C(15)			0.000	-0.001	0.000	0.001
C(16)			0.000	0.001	0.000	-0.001
C(17)			0.000	0.001	0.000	-0.001
C(18)			0.000	0.001	0.000	-0.002
C(19)			0.000	0.001	0.000	-0.001
C(20)			0.000	0.000	0.000	0.000
C(21)			0.000	0.000	0.000	0.000
C(22)			0.000	0.001	0.000	-0.001
C(23)			0.000	0.000	0.000	0.000
C(24)			0.000	0.001	0.000	-0.001
C(25)			0.000	0.001	0.000	-0.001
H(26)			0.000	0.000	0.000	0.000
H(27)			0.000	0.000	0.000	0.000
H(28)			0.000	0.000	0.000	0.000
H(29)			0.000	0.000	0.000	0.000
H(30)			0.000	0.000	0.000	0.000
H(31)			0.000	0.000	0.000	0.000
H(32)			0.000	0.000	0.000	0.000
H(33)			0.000	0.000	0.000	0.000
H(34)			0.000	0.000	0.000	0.000
H(35)			0.000	0.000	0.000	0.000
H(36)			0.000	0.000	0.000	0.000

H(37)			0.000	0.000	0.000	0.000
S(38)	-0.113	0.024	-0.018	-0.039	0.003	0.035
C(39)	-0.015	-0.006	-0.002	-0.003	0.000	0.002
H(40)	-0.004	-0.001	0.000	-0.001	0.000	0.001
H(41)	-0.004	-0.002	-0.001	-0.001	0.000	0.000
C(42)			-0.004	-0.003	0.000	0.001
H(43)			-0.001	-0.001	0.000	0.000
C(44)					0.000	0.000
O(45)					0.000	0.000
N(46)					0.000	0.000
H(47)					0.000	0.000
N(52)					0.000	0.000
H(53)					0.000	0.000
C(54)					0.000	0.000
O(55)					0.000	0.000

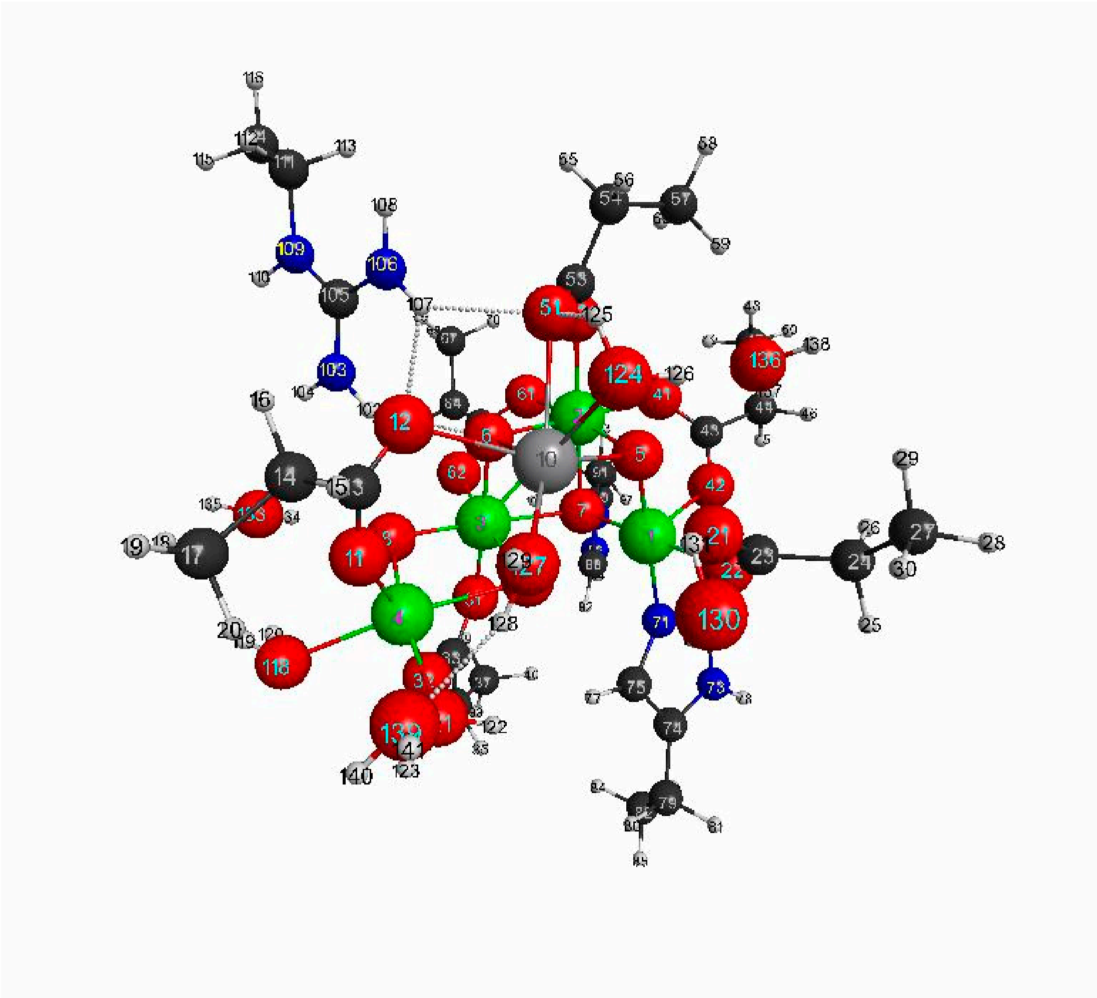

Figure S-8. Geometry of the active site of OEC (pdb entry:5b66). The numbering of the atoms is used for the following figures to show linear response functions. This figure is the same as figure 1 , but the size is much larger than figure 8.

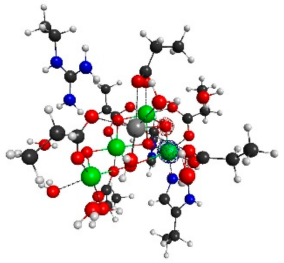

Mn(1)

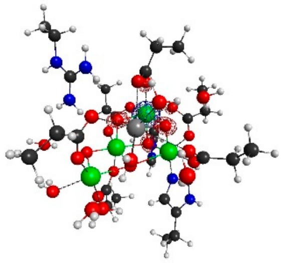

Mn(2)

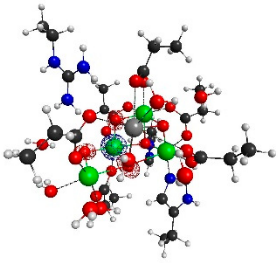

Mn(3)

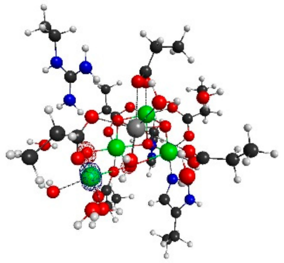

Mn(4)

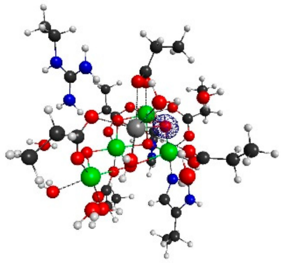

O(5)

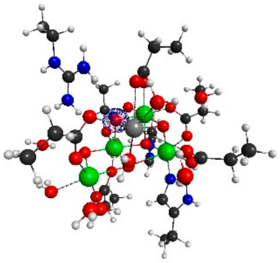

O(6)

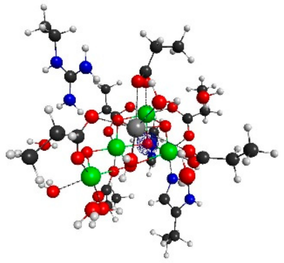

O(7)

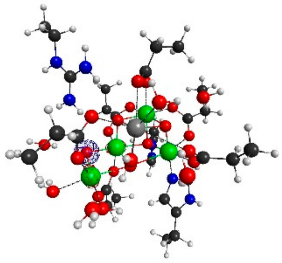

O(8)

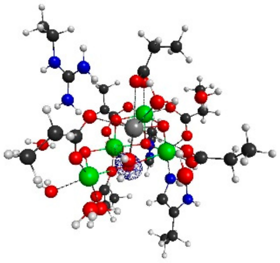

O(9)

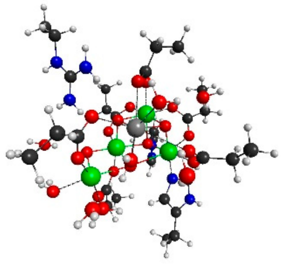

Ca(10)

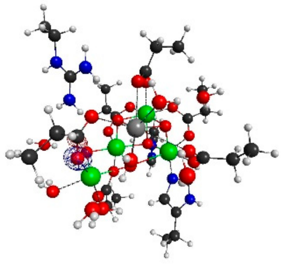

O(11)

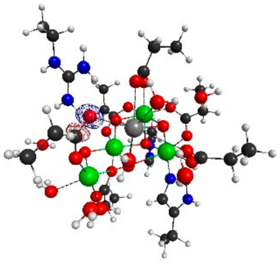

O(12)

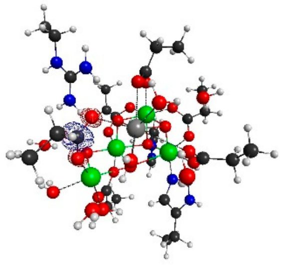

C(13)

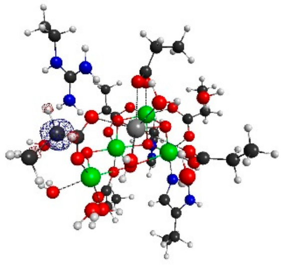

C(14)

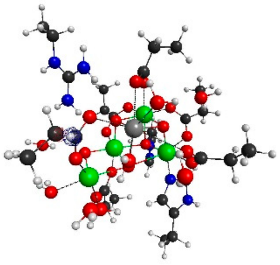

H(15)

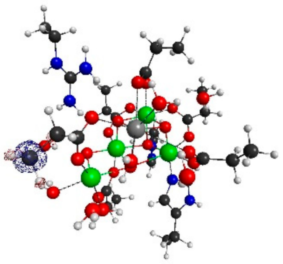

C(17)

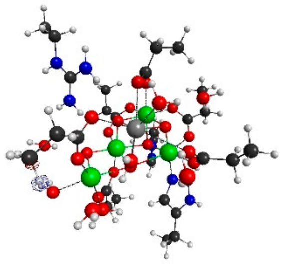

H(20)

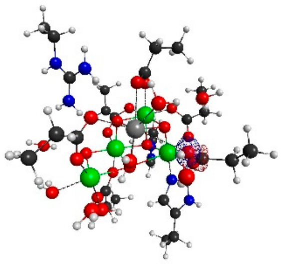

O(21)

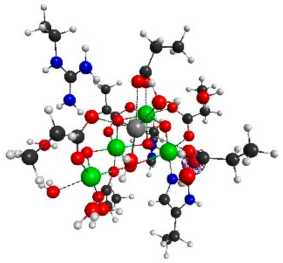

O(22)

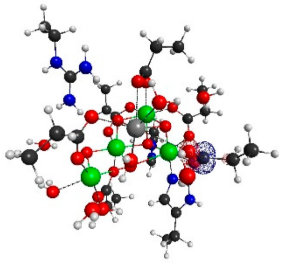

C(23)

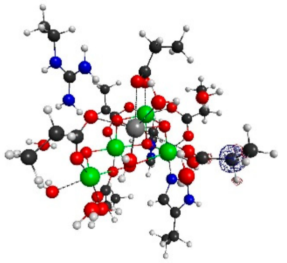

C(24)

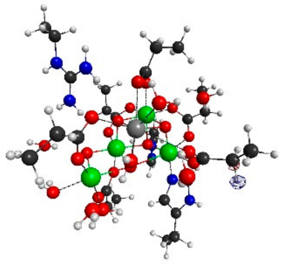

H(25)

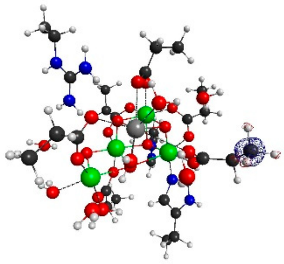

C(27)

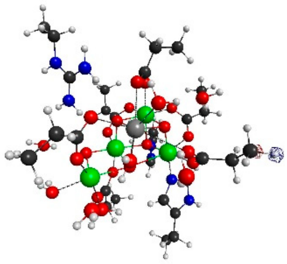

H(28)

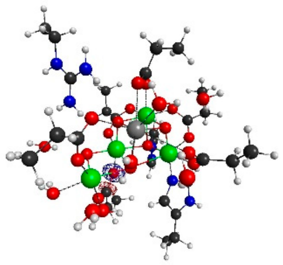

O(31)

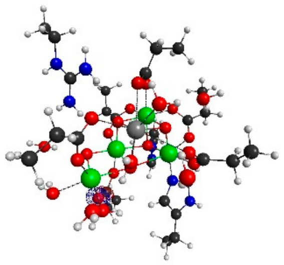

O(32)

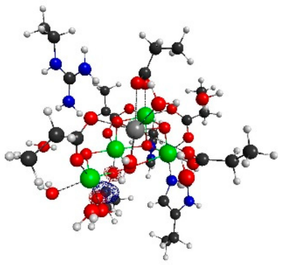

C(33)

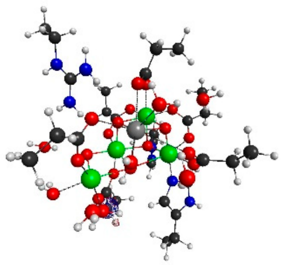

C(34)

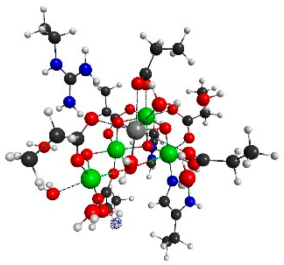

H(35)

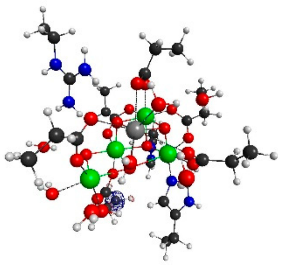

C(37)

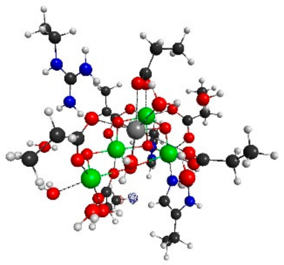
H(40)

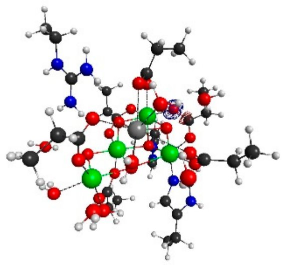
O(41)

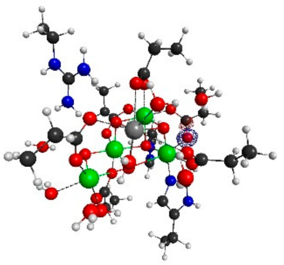
O(42)

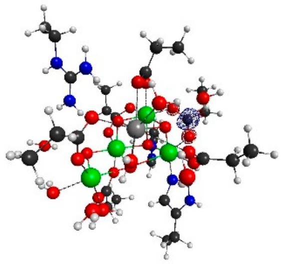
C(43)

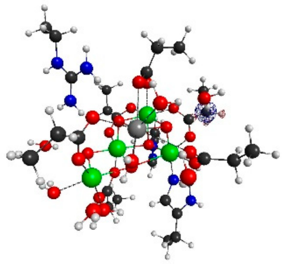
C(44)

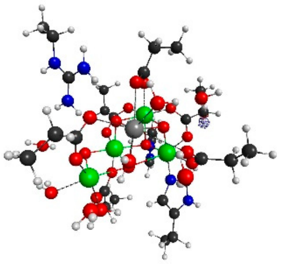
H(45)

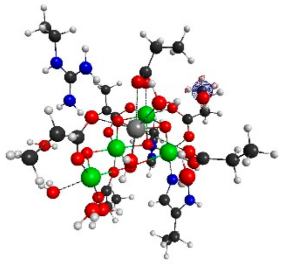
C(47)

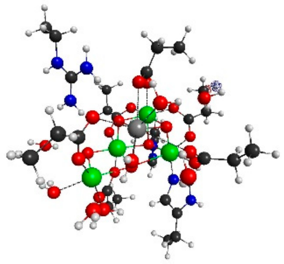
H(50)

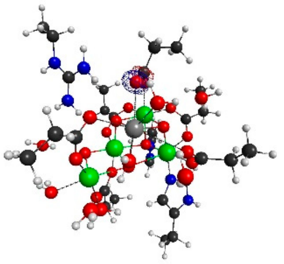
O(51)

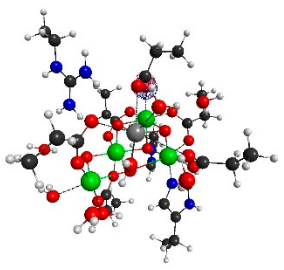
O(52)

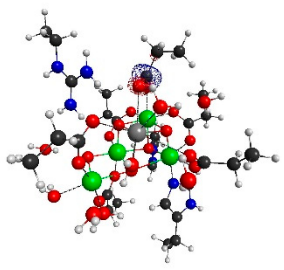
C(53)

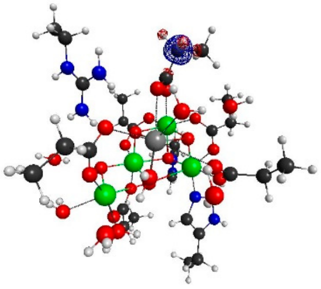
C(54)

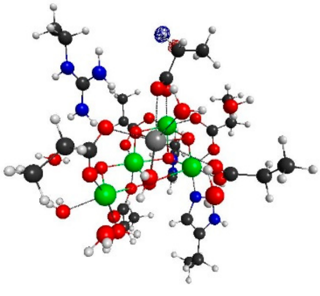
H(55)

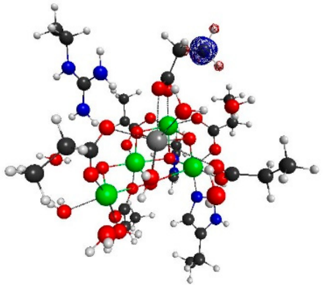
C(57)

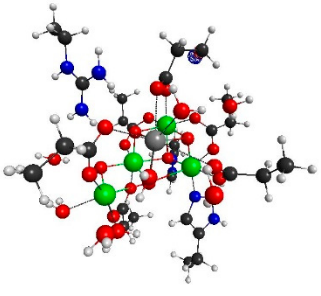
H(60)

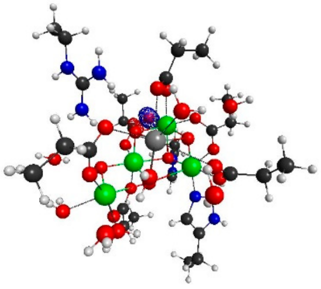
O(61)
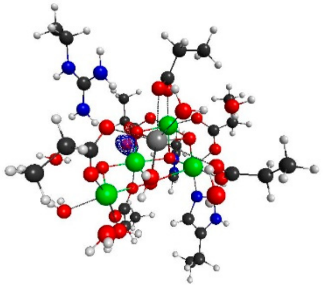
O(62)
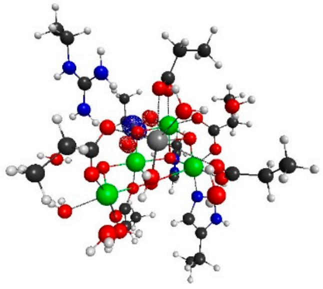
C(63)
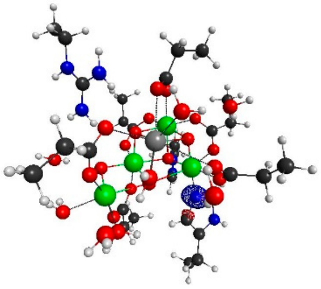
N(71)
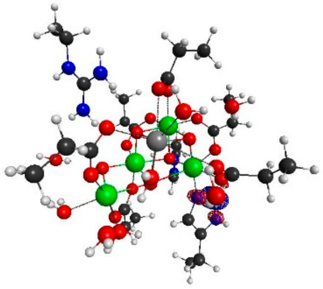
C(72)
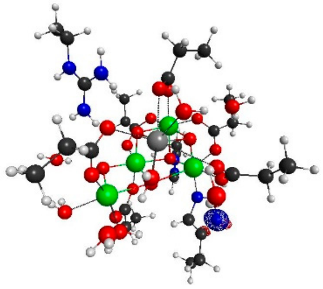
N(73)
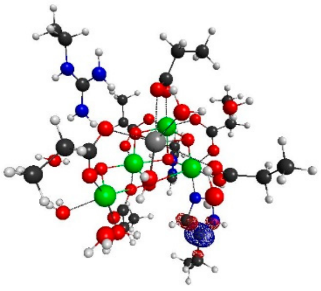
C(74)
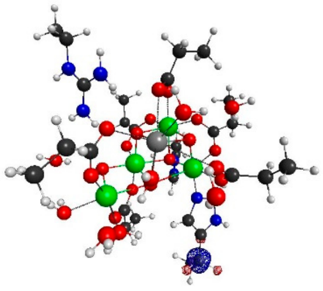
C(79)
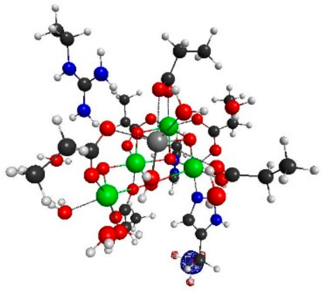
C(82)
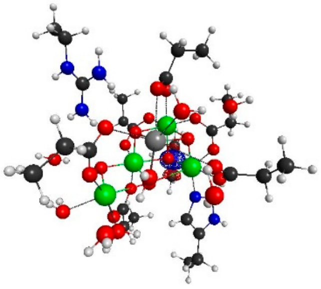
N(87)
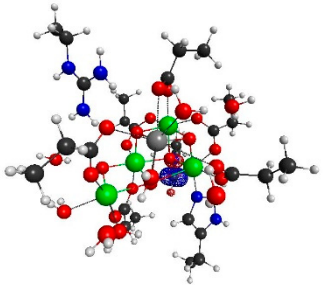
C(88)
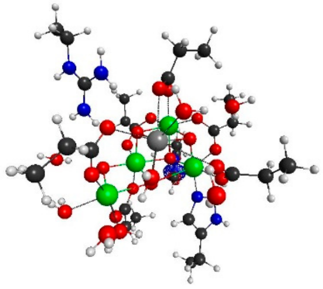
N(89)
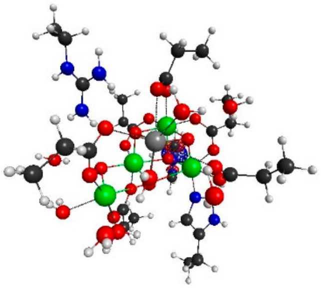
C(90)
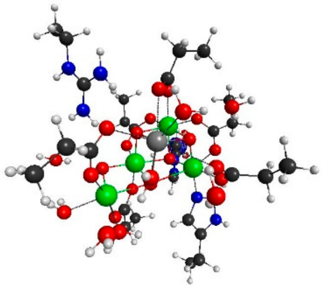
C(95)
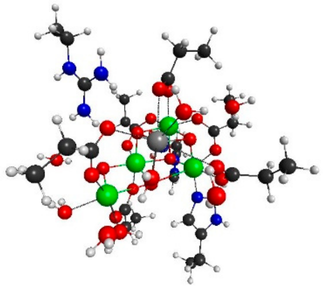
C(98)

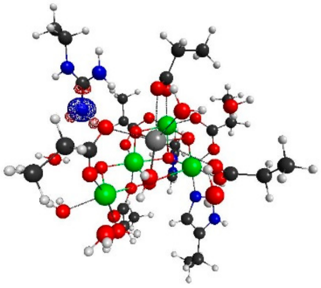

N(103)

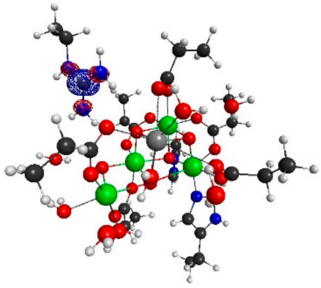

C(105)

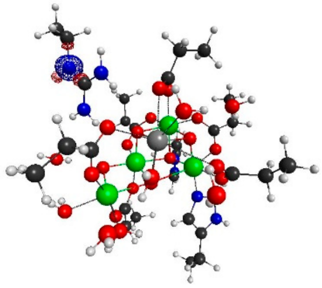

N(109)

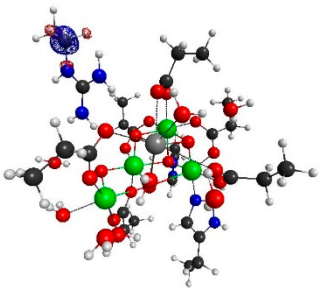

C(111)

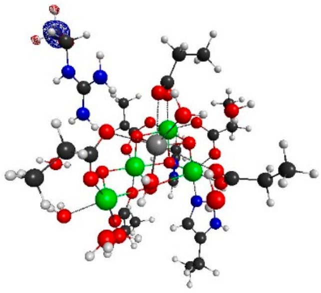

C(114)

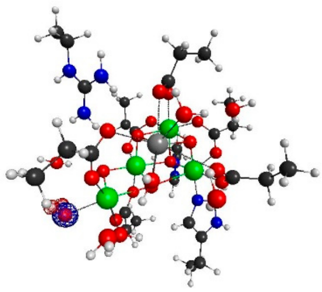

O(118)

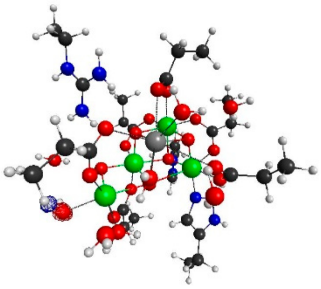

H(119)

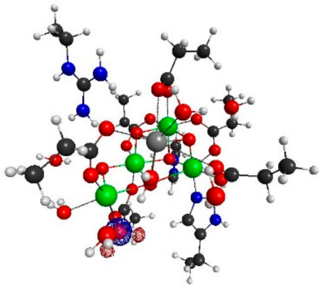

O(121)

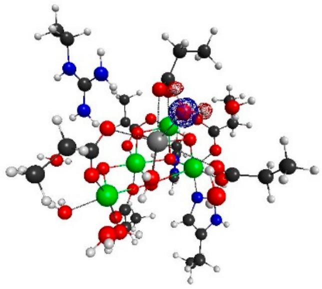

O(124)

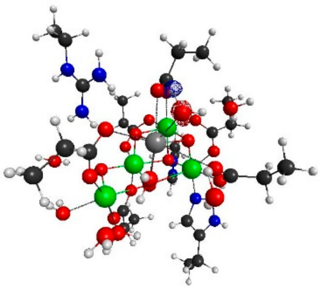

H(125)

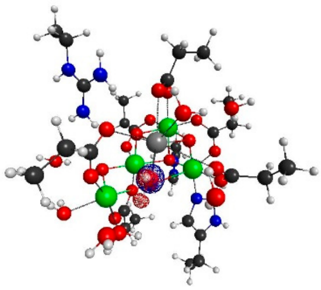

O(127)

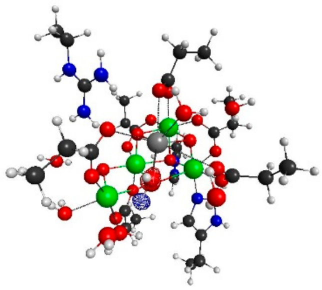

H(128)

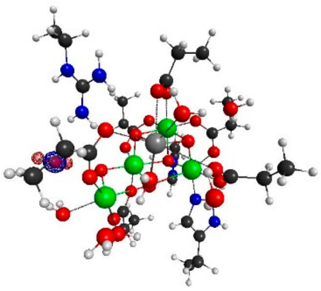

O(133)

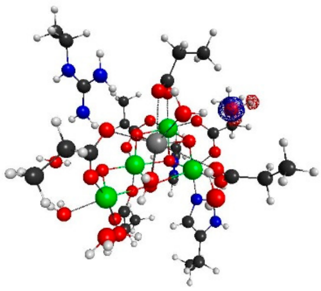

O(136)

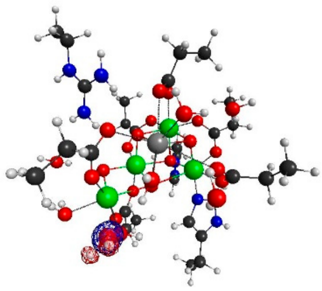

O(139)

Figure S-9. Isosurfaces of linear response functions of density for the perturbations on atomic sites of OEC. The threshold of $\delta\rho(\boldsymbol{r})/\delta v(I)$ are ± 0.1 (+: blue, −: red). The atomic symbols and those numbers in parentheses below the figures indicate the atoms (I) to which the virtual perturbations are applied.

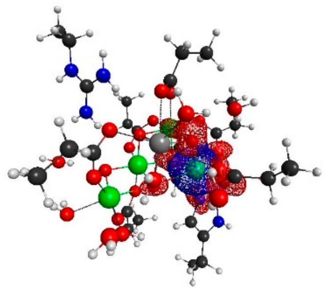

Mn(1)

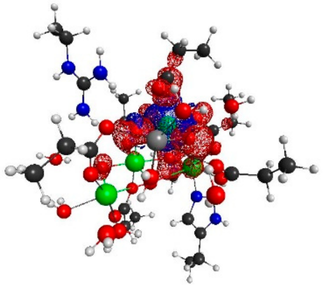

Mn(2)

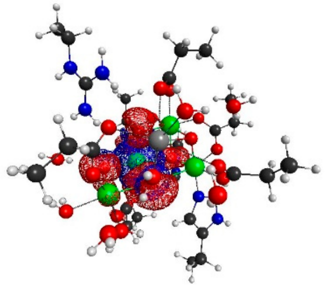

Mn(3)

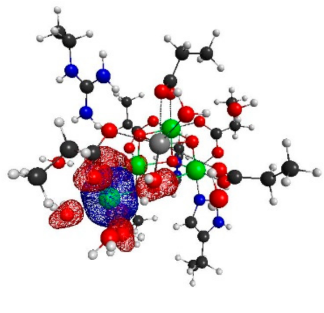

Mn(4)

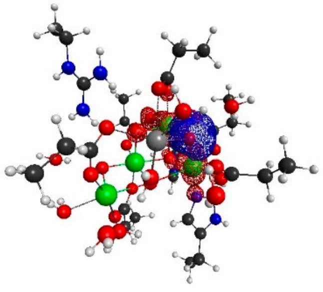

O(5)

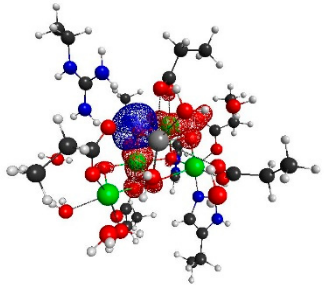

O(6)

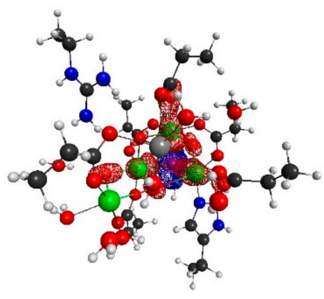

O(7)

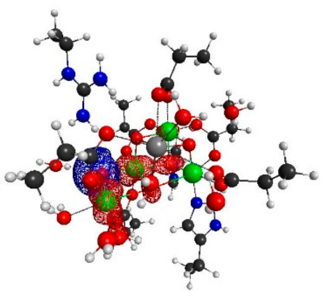

O(8)

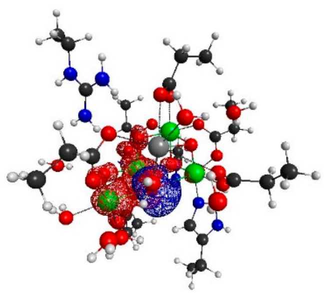

O(9)

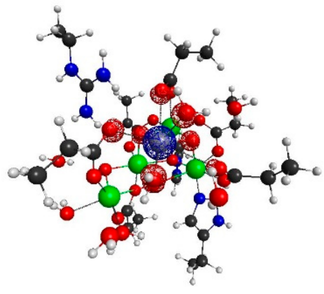

Ca(10)

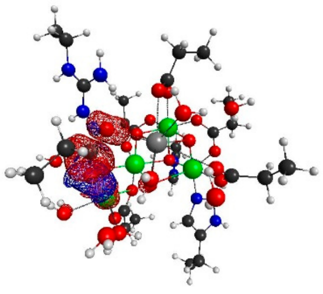

O(11)

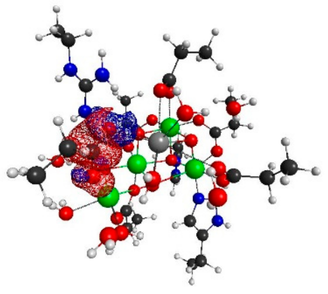

O(12)

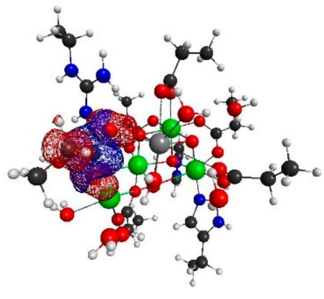

C(13)

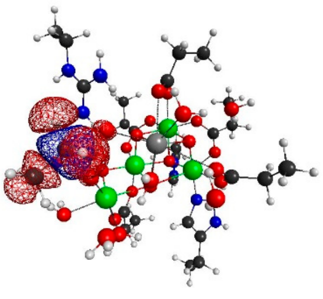

C(14)

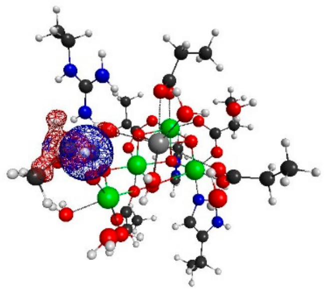

H(15)

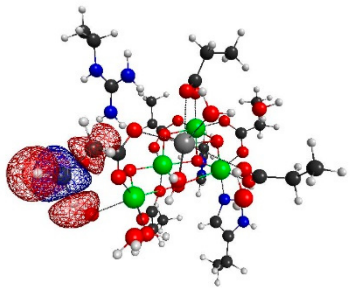

C(17)

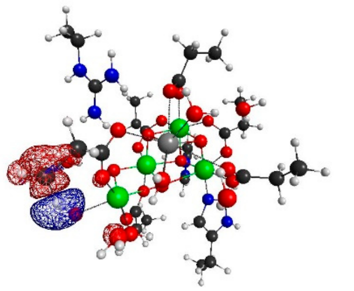

H(20)

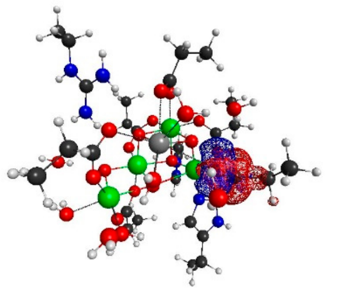

O(21)

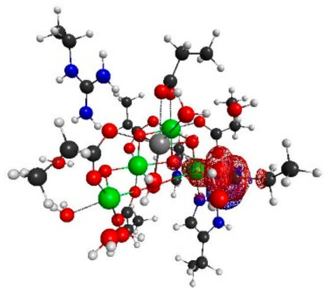

O(22)

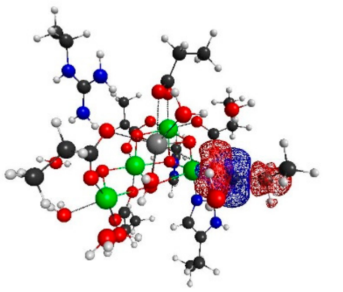

C(23)

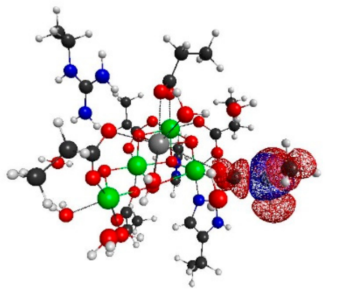

C(24)

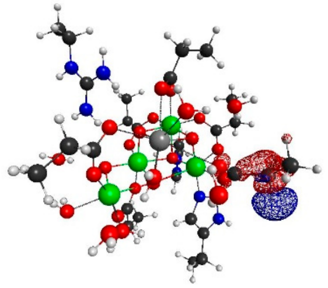

H(25)

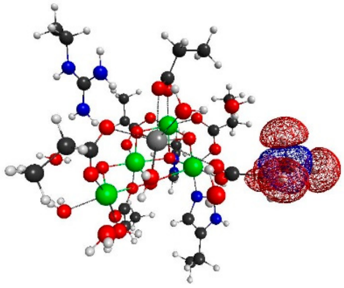

C(27)

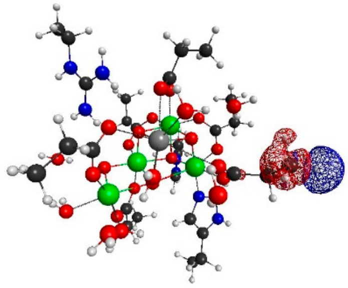

H(28)

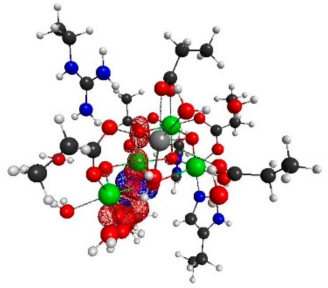

O(31)

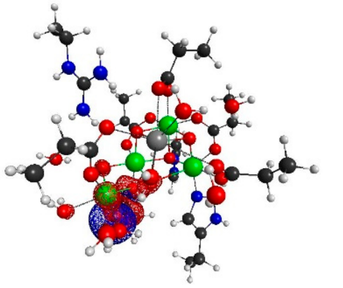

O(32)

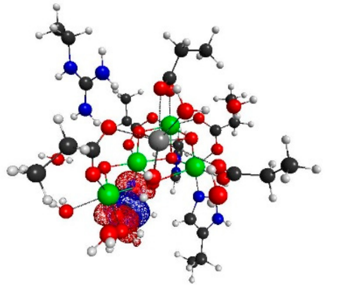

C(33)

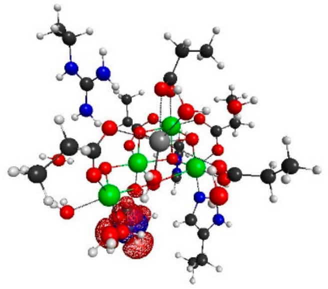

C(34)

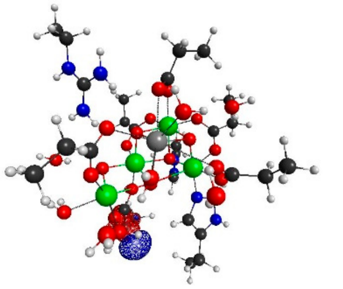

H(35)

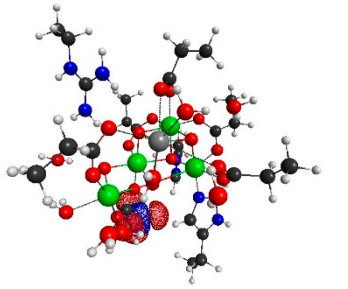

C(37)

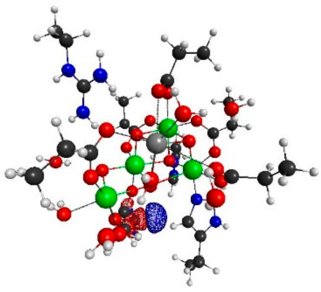
H(40)

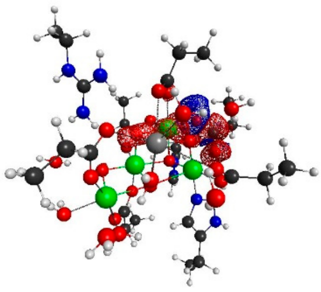
O(41)

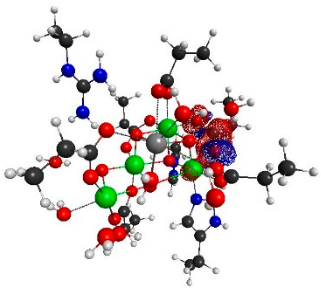
O(42)

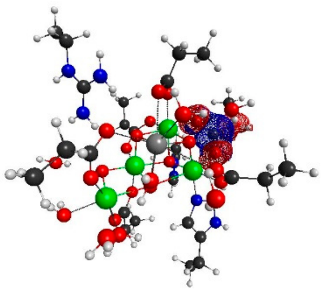
C(43)

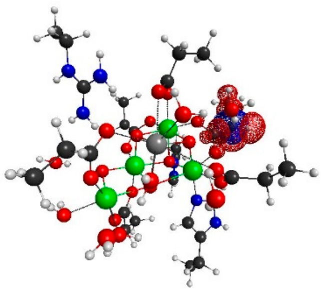
C(44)

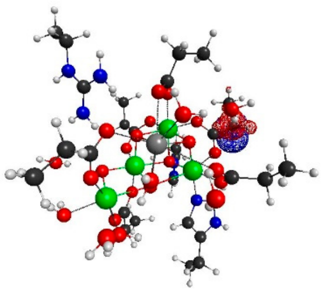
H(45)

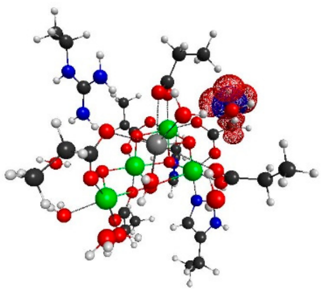
C(47)

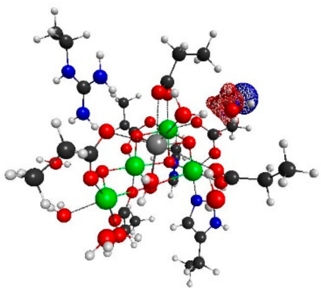
H(50)

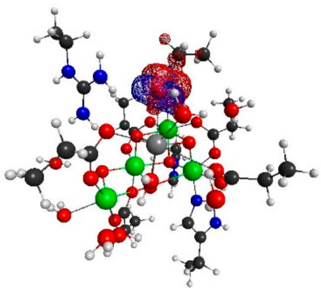
O(51)

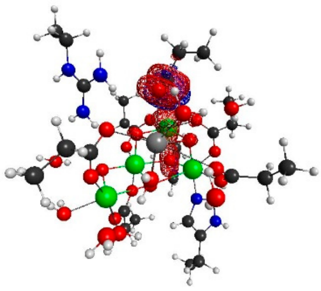
O(52)

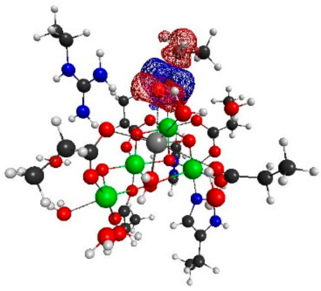
C(53)

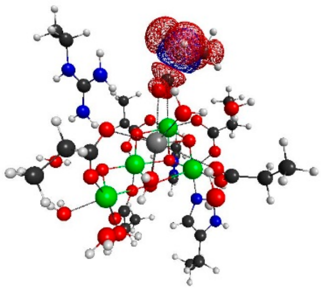
C(54)

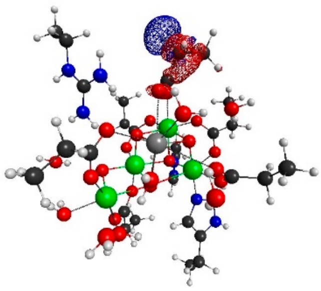
H(55)

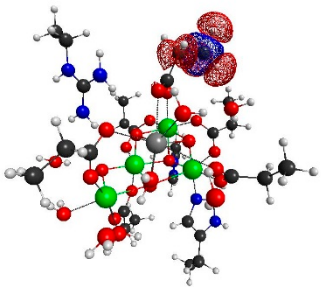
C(57)

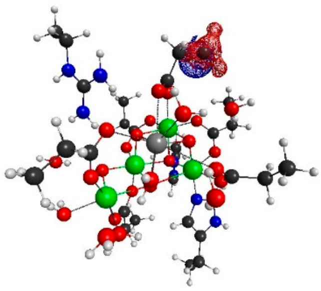
H(60)

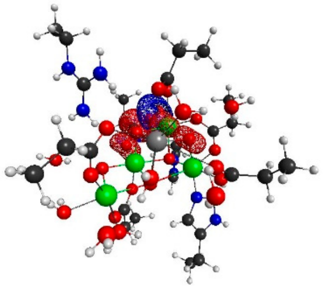

O(61)

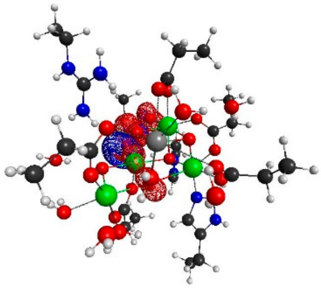

O(62)

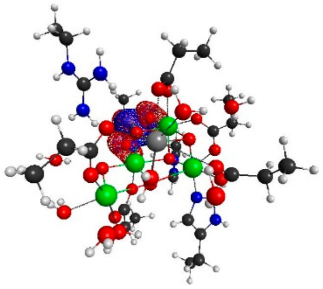

C(63)

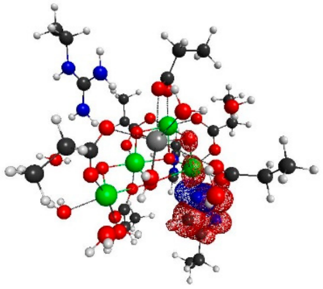

N(71)

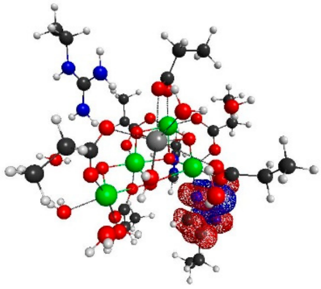

C(72)

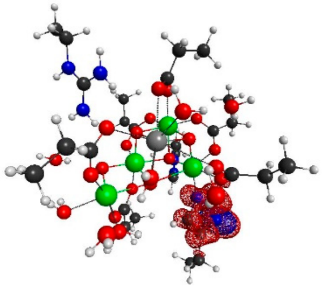

N(73)

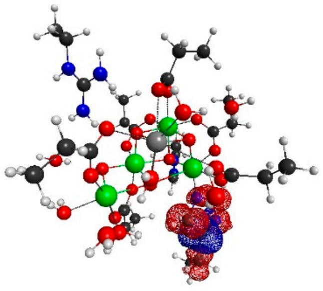

C(74)

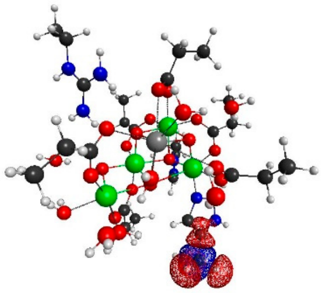

C(79)

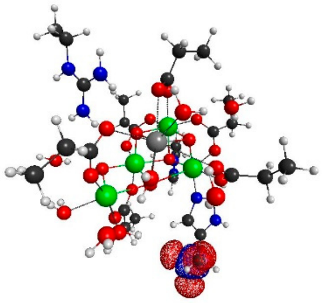

C(82)

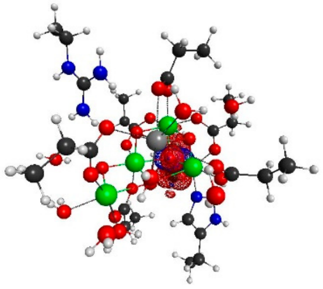

N(87)

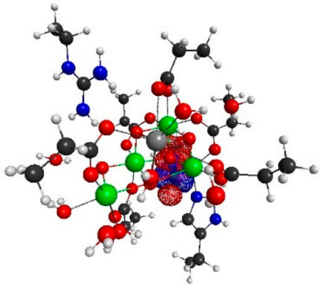

C(88)

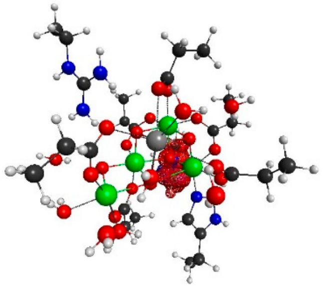

N(89)

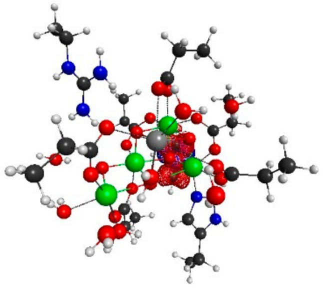

C(90)

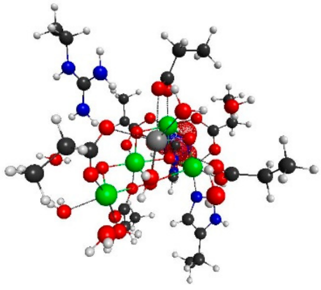

C(95)

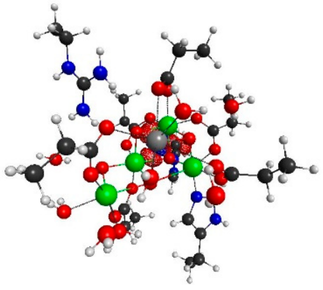

C(98)

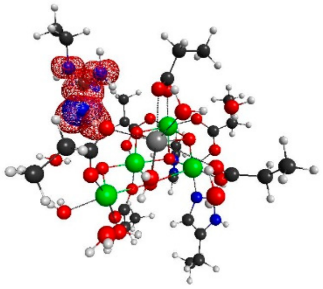

N(103)

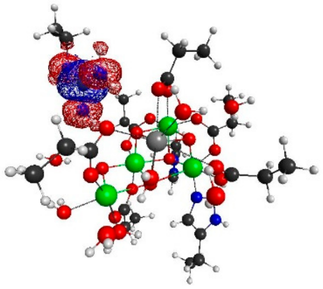

C(105)

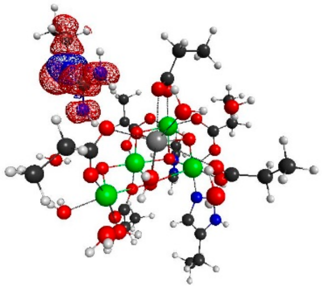

N(109)

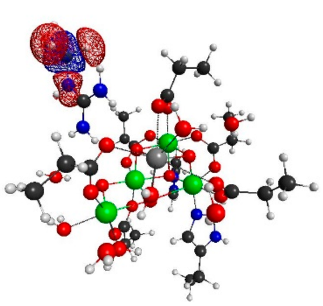

C(111)

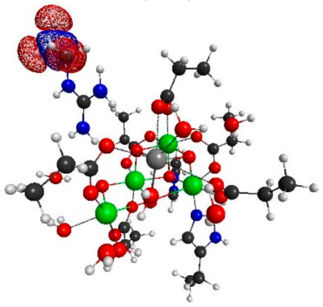

C(114)

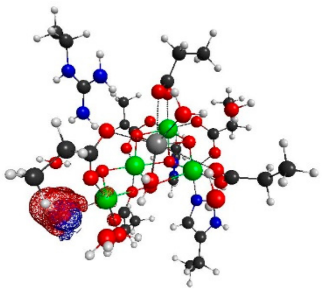

O(118)

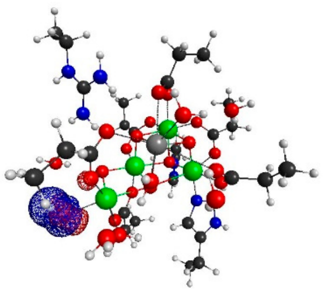

H(119)

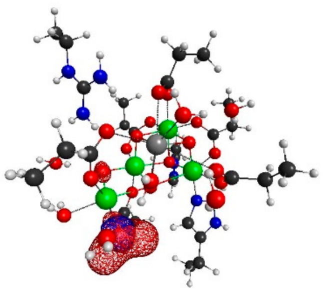

O(121)

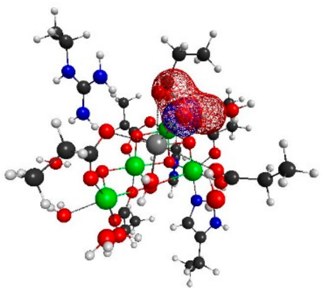

O(124)

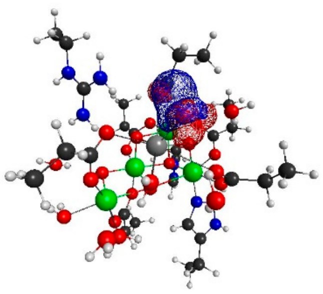

H(125)

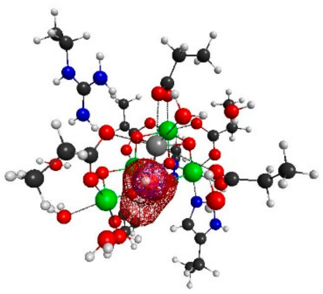

O(127)

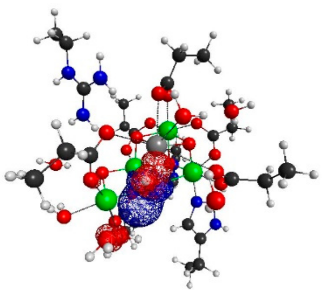

H(128)

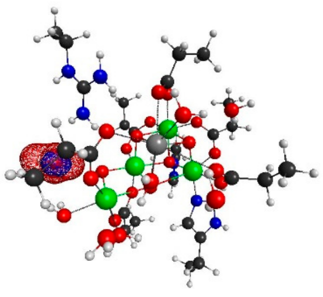

O(133)

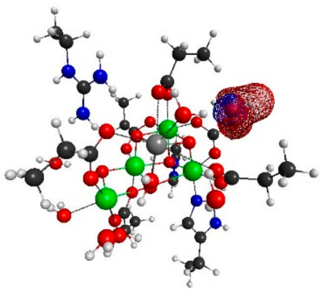

O(136)

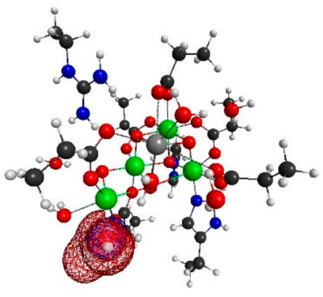

O(139)

Figure S-10. Isosurfaces of linear response functions of density for the perturbations on atomic sites of OEC. The threshold of $\delta\rho(\boldsymbol{r})/\delta v(I)$ are ± 0.01 (+: blue, −: red). The atomic symbols and those numbers in parentheses below the figures indicate the atoms (I) to which the virtual perturbations are applied.

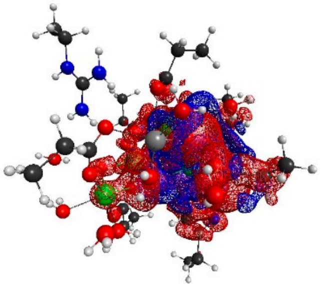

Mn(1)

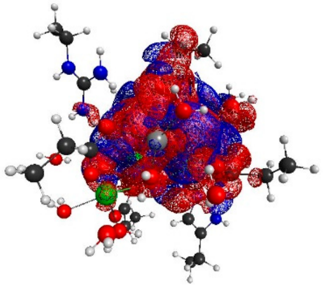

Mn(2)

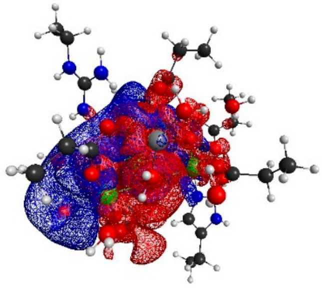

Mn(3)

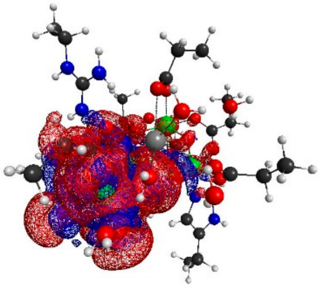

Mn(4)

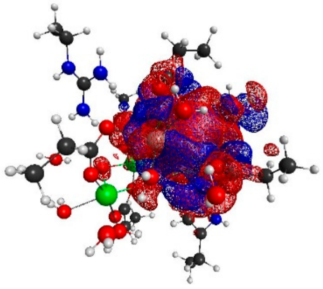

O(5)

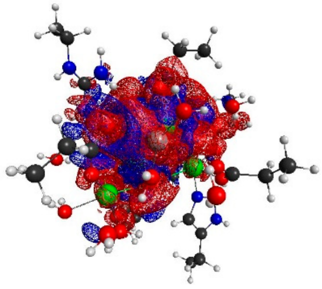

O(6)

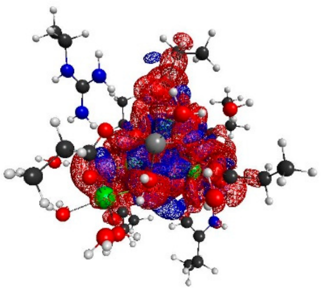

O(7)

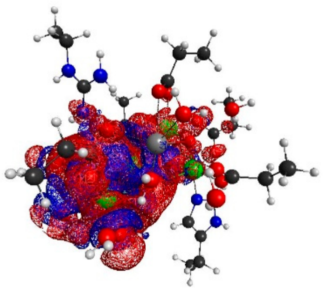

O(8)

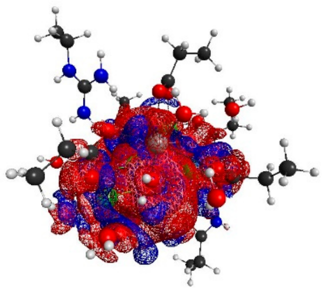

O(9)

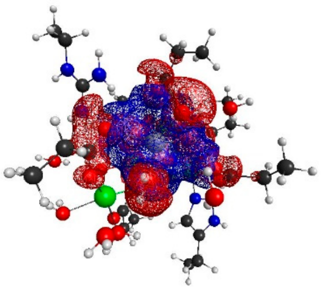

Ca(10)

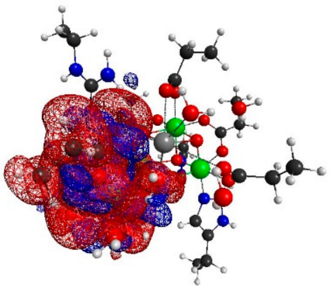

O(11)

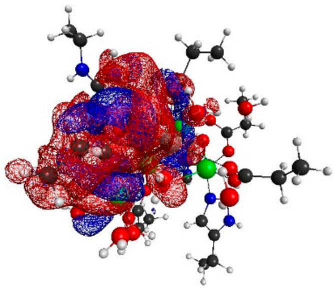

O(12)

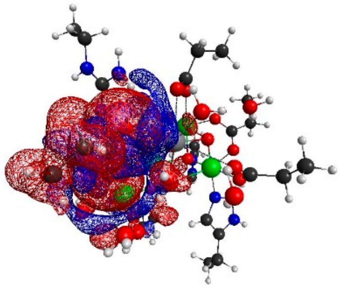

C(13)

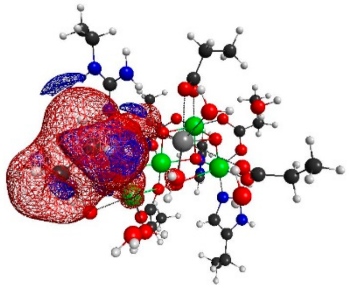

C(14)

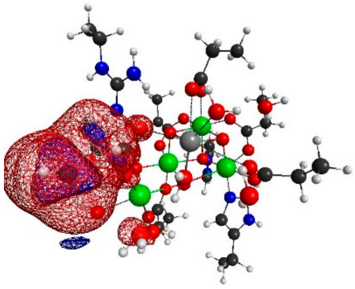

H(15)

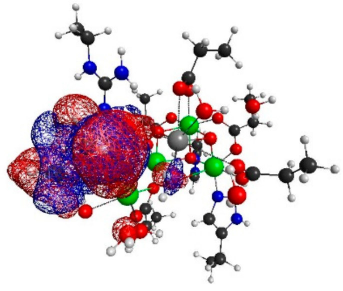

C(17)

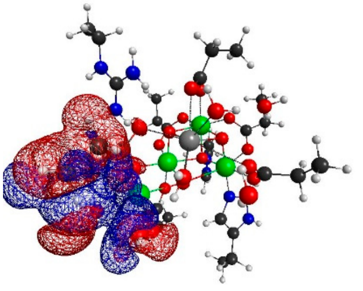

H(20)

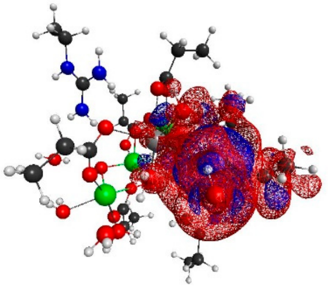

O(21)

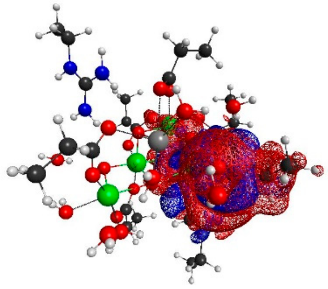

O(22)

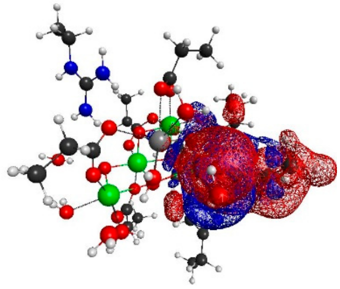

C(23)

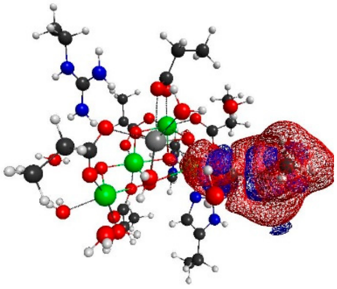

C(24)

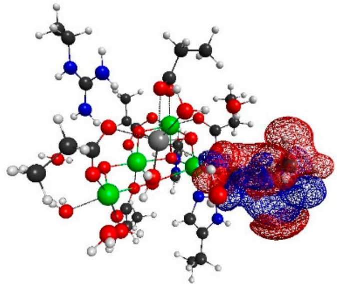

H(25)

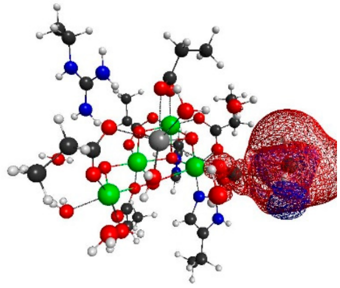

C(27)

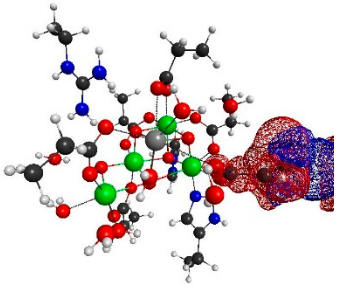

H(28)

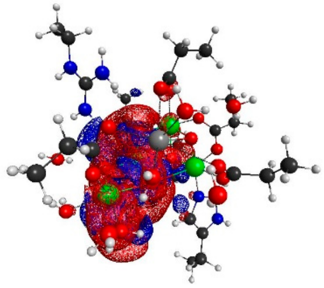

O(31)

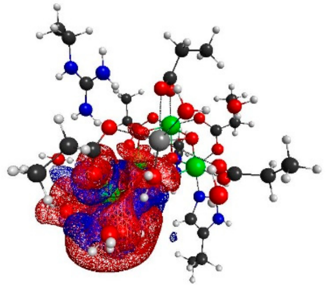

O(32)

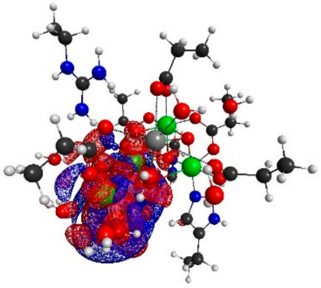

C(33)

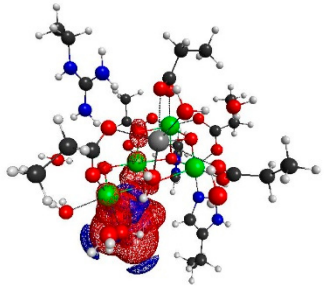

C(34)

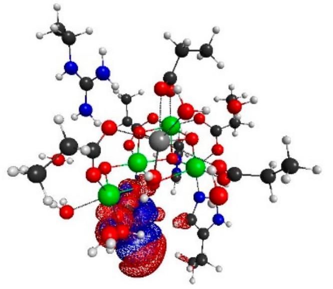

H(35)

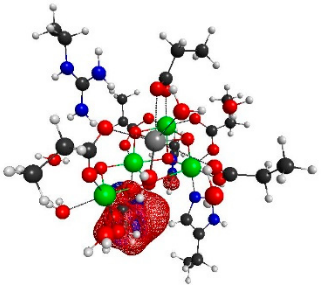

C(37)

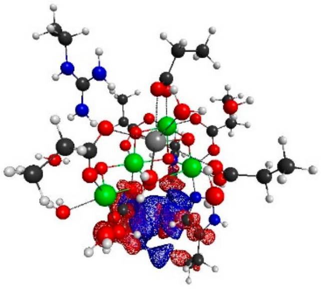 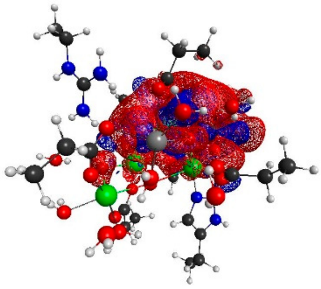 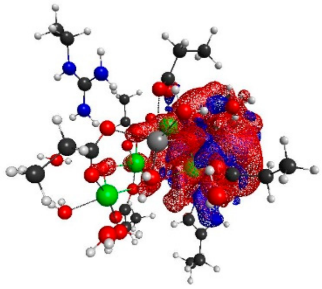

H(40) O(41) O(42)

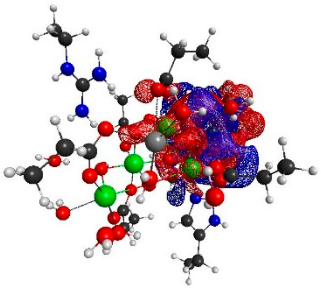 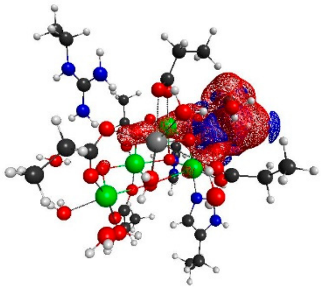 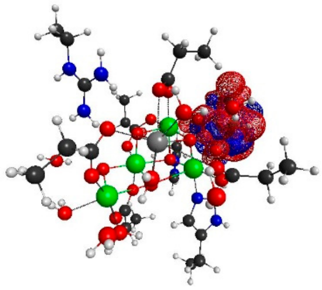

C(43) C(44) H(45)

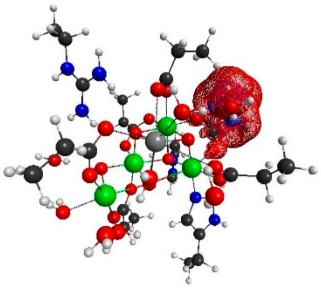 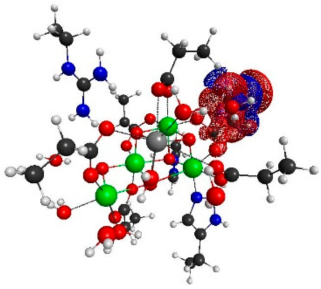 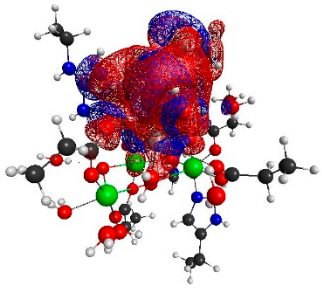

C(47) H(50) O(51)

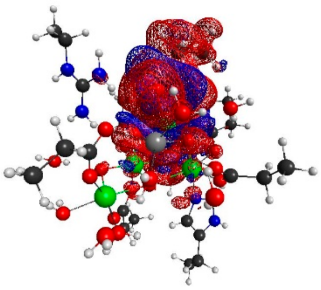 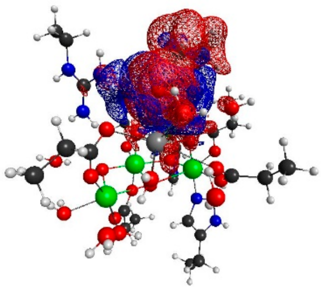 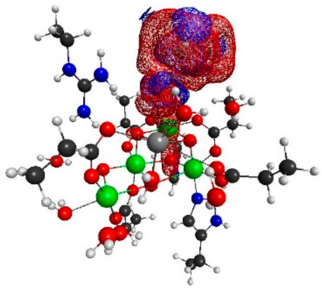

O(52) C(53) C(54)

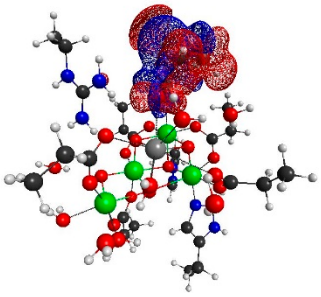 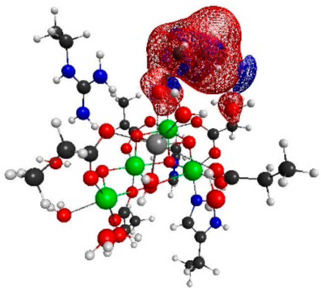 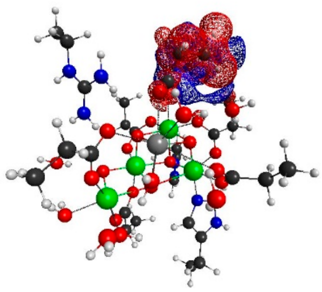

H(55) C(57) H(60)

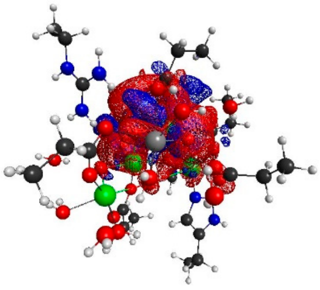

O(61)

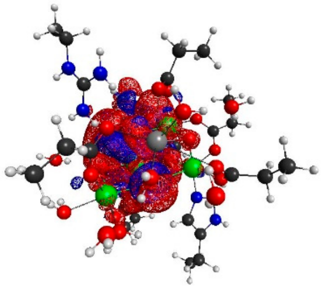

O(62)

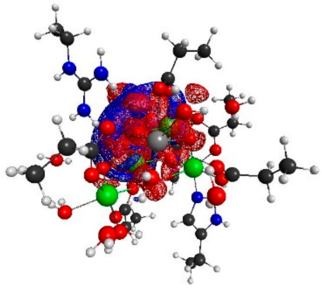

C(63)

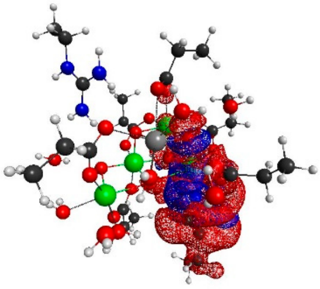

N(71)

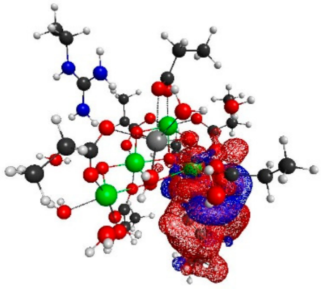

C(72)

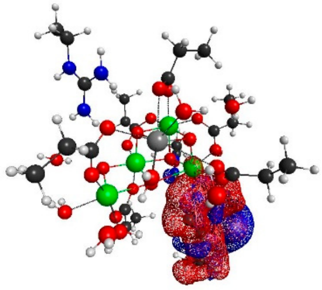

N(73)

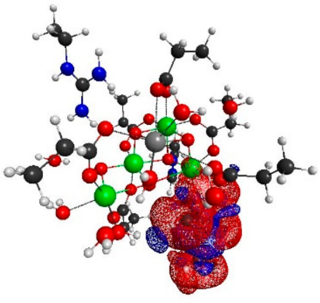

C(74)

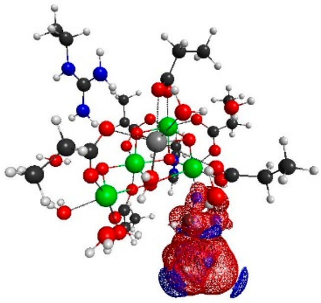

C(79)

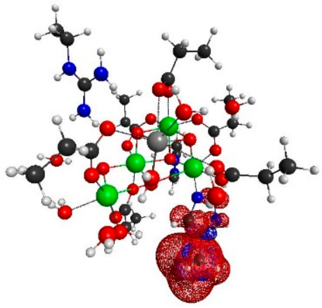

C(82)

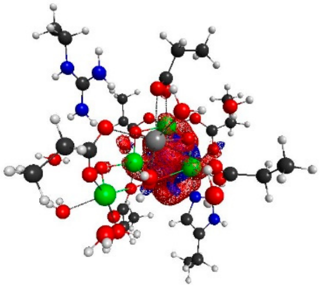

N(87)

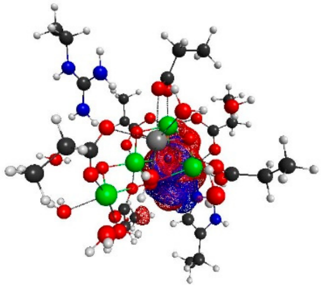

C(88)

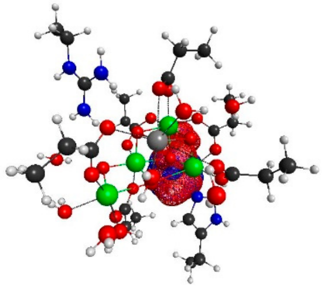

N(89)

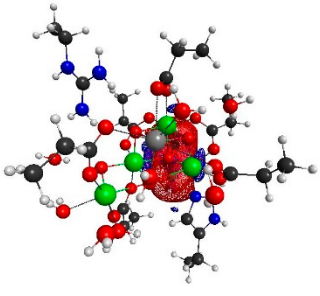

C(90)

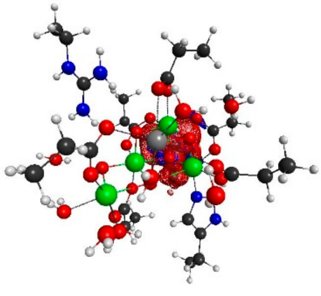

C(95)

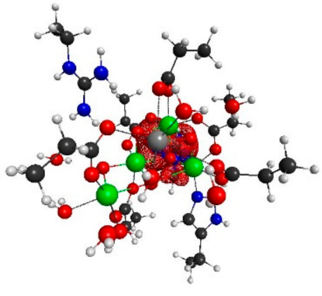

C(98)

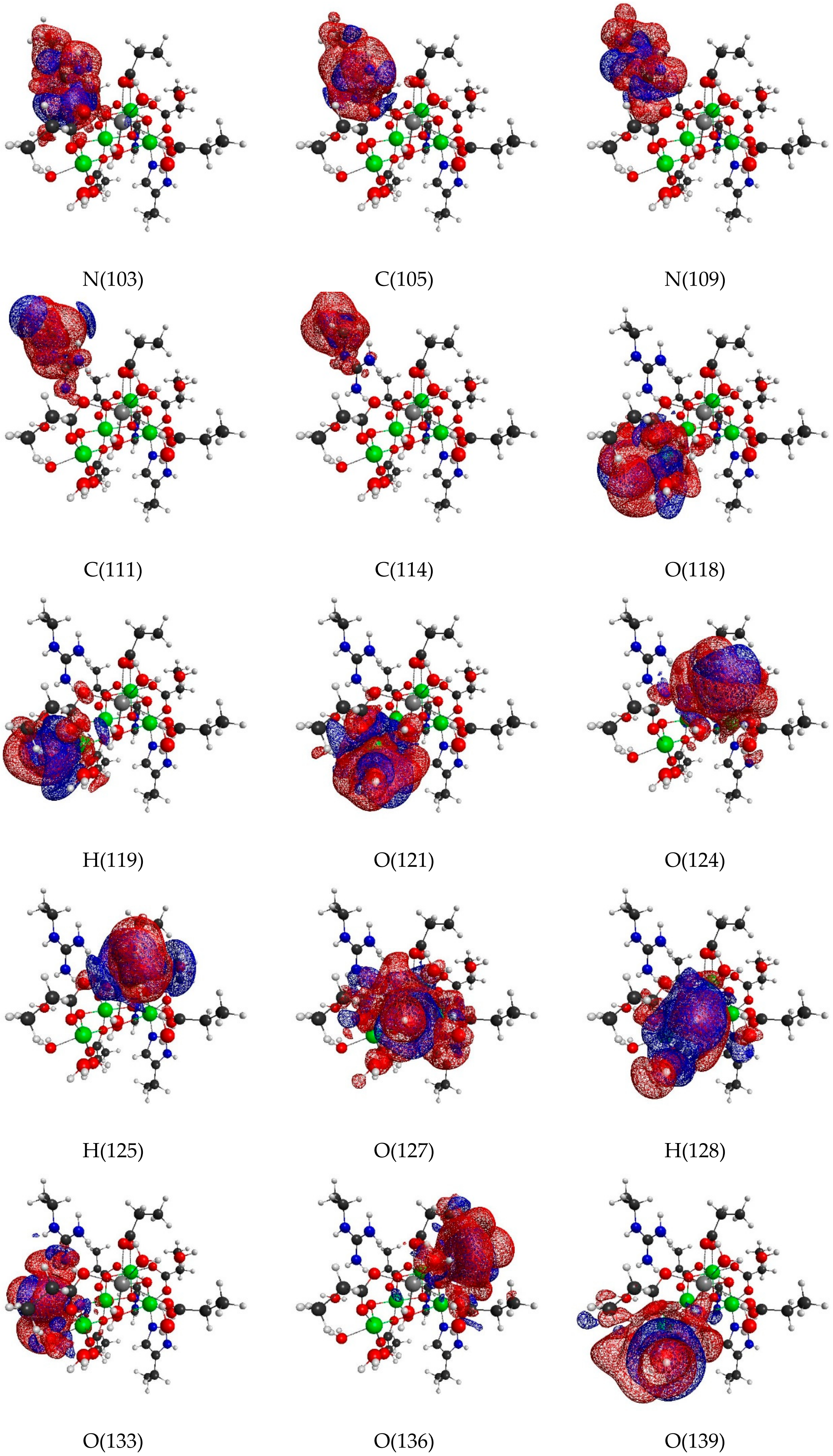

Figure S-11. Isosurfaces of linear response functions of density for the perturbations on atomic sites of OEC. The threshold of $\delta\rho(\boldsymbol{r})/\delta v(I)$ are ± 0.001 (+: blue, −: red). The atomic symbols and those numbers in parentheses below the figures indicate the atoms (I) to which the virtual perturbations are applied.

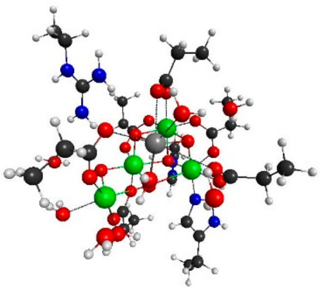

Mn(1)

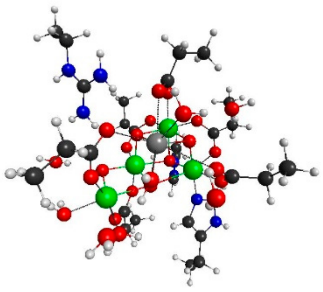

Mn(2)

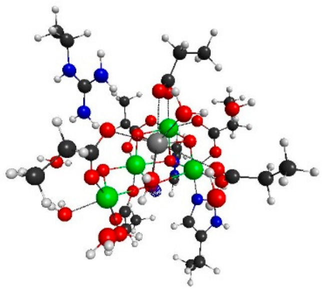

Mn(3)

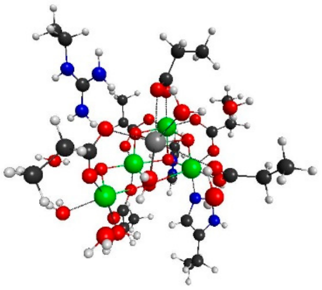

Mn(4)

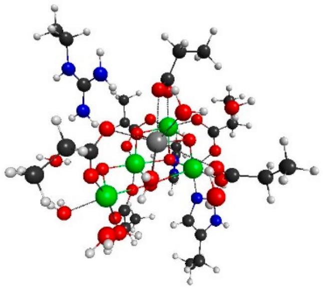

O(5)

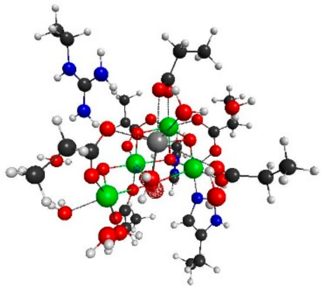

O(9)

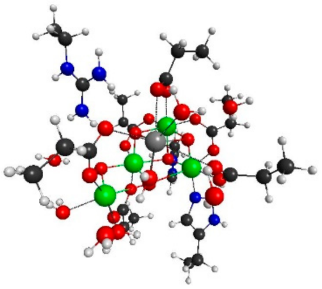

O(32)

Figure S-12. Isosurfaces of linear response functions of density for the perturbations on atomic sites of OEC (pdb entry: 5b66). The threshold of $\delta\rho_z(\boldsymbol{r})/\delta v_m(I)$ are ± 0.1 (+: blue, −: red). The atomic symbols and those numbers in parentheses below the figures indicate the atoms (I) to which the virtual perturbations are applied. The figures of $\delta\rho_z(\boldsymbol{r})/\delta v_m(I)$ that vanishes for this threshold are omitted.

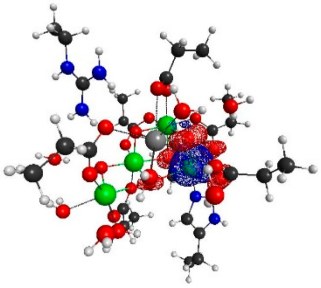
Mn(1)

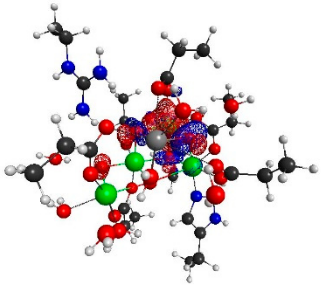
Mn(2)

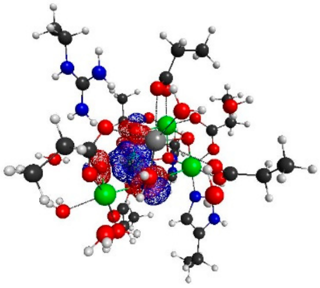
Mn(3)

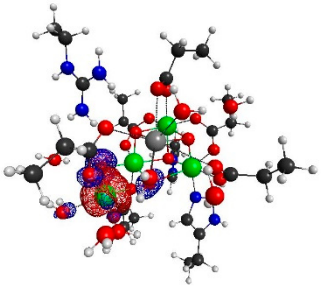
Mn(4)

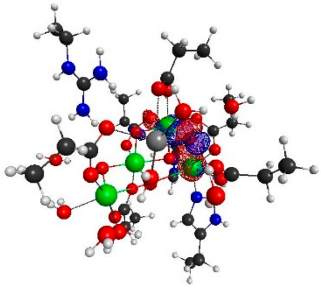
O(5)

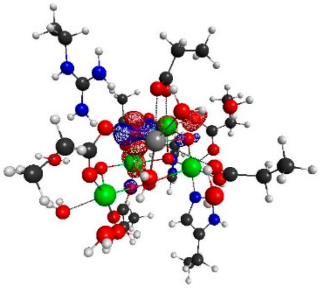
O(6)

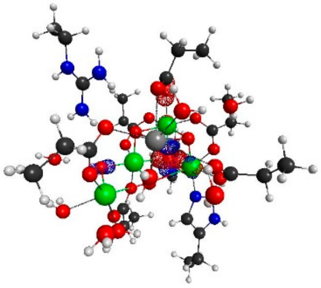
O(7)

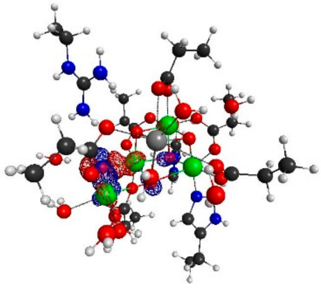
O(8)

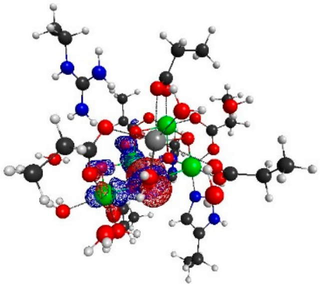
O(9)

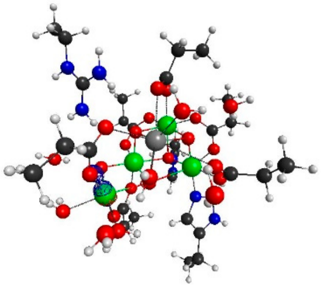
O(11)

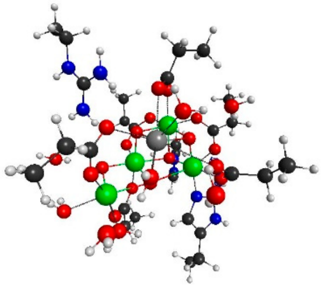
O(21)

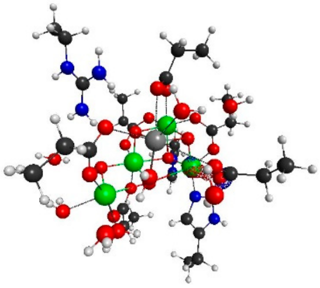
O(22)

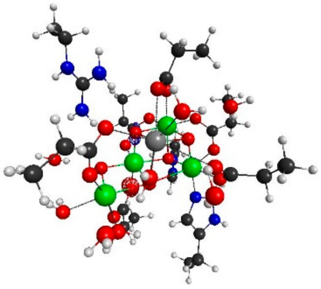
O(31)

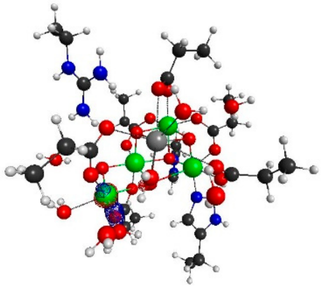
O(32)

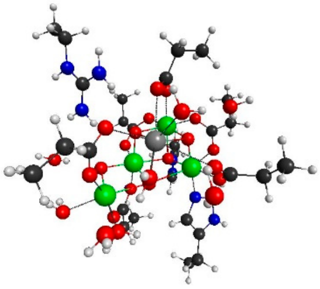
C(33)

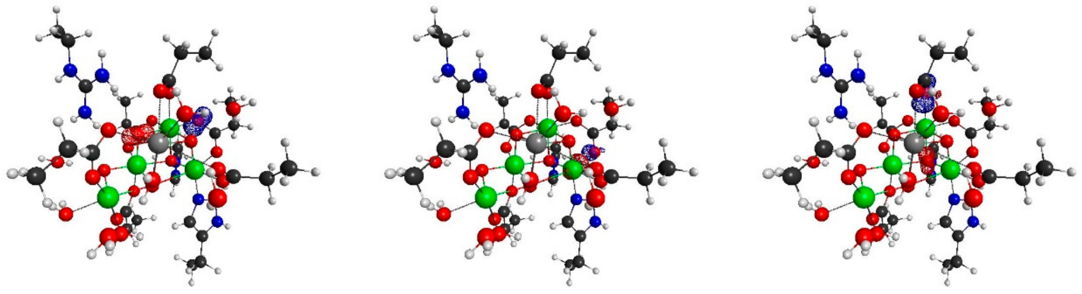

O(41) O(42) O(52)

Figure S-13. Isosurfaces of linear response functions of density for the perturbations on atomic sites of OEC (pdb entry: 5b66). The threshold of $\delta\rho_z(\boldsymbol{r})/\delta v_m(I)$ are ± 0.01 (+: blue, −: red). The atomic symbols and those numbers in parentheses below the figures indicate the atoms (I) to which the virtual perturbations are applied. The figures of $\delta\rho_z(\boldsymbol{r})/\delta v_m(I)$ that vanishes for this threshold are omitted.

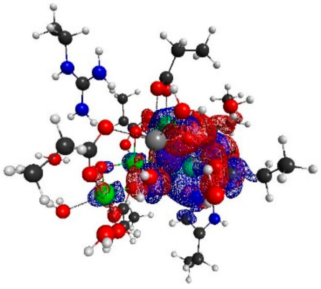
Mn(1)

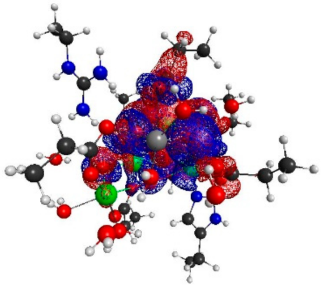
Mn(2)

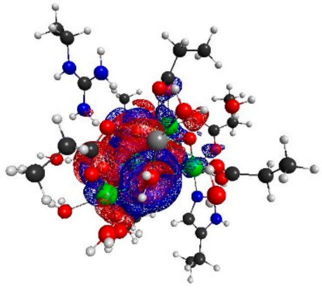
Mn(3)

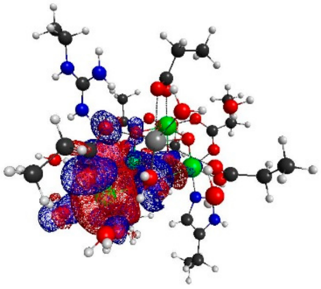
Mn(4)

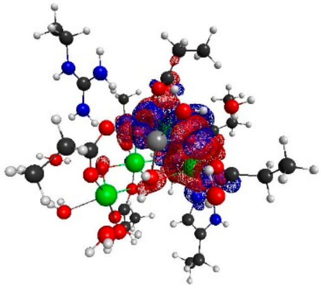
O(5)

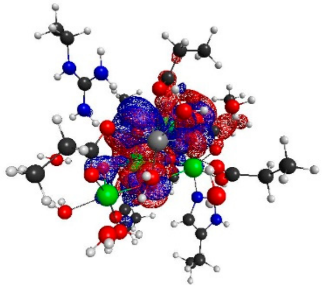
O(6)

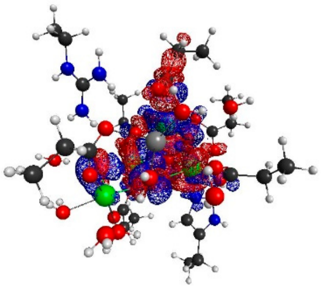
O(7)

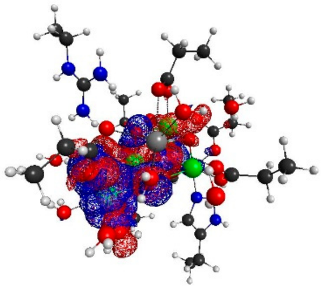
O(8)

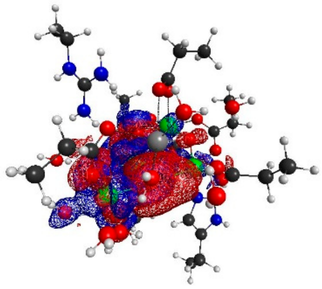
O(9)

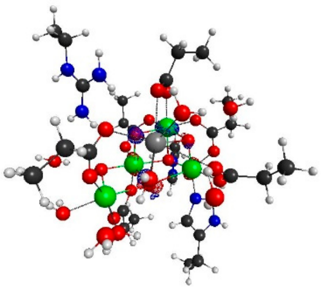
Ca(10)

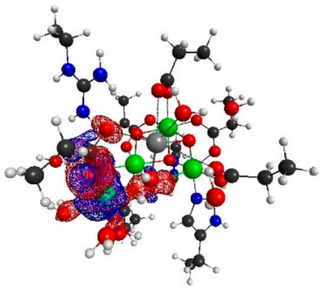
O(11)

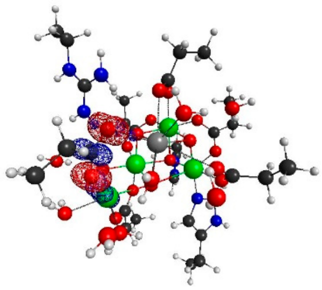
O(12)

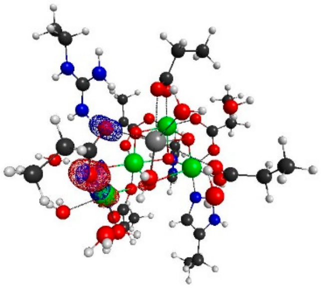
C(13)

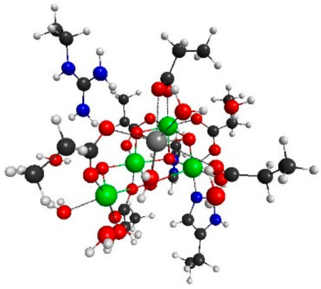
C(14)

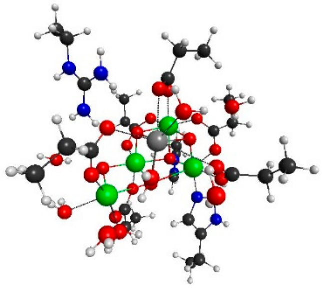
H(16)

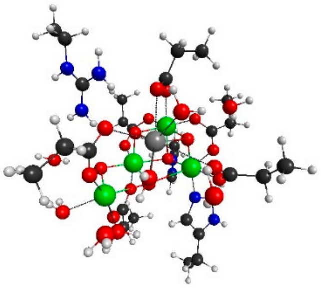
C(17)

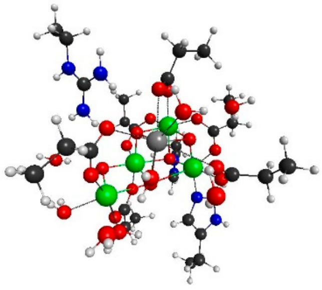
H(20)

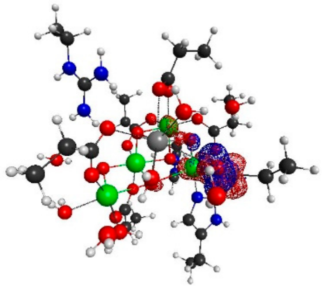
O(21)

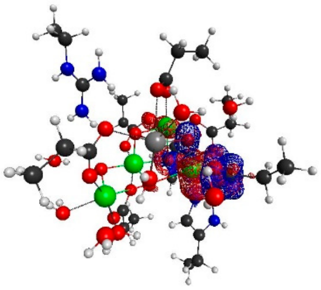
O(22)

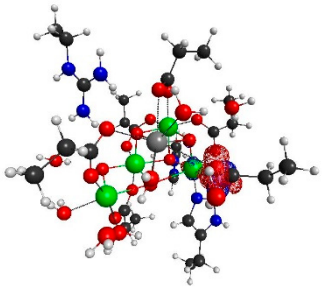
C(23)

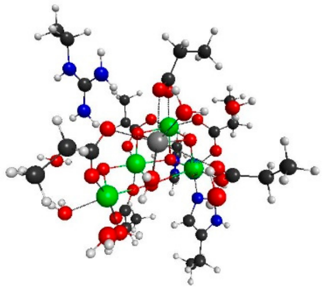
C(24)

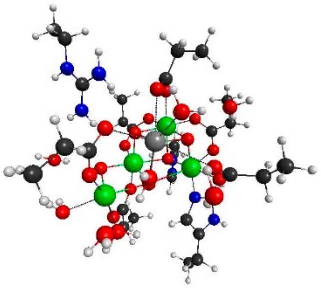
H(26)

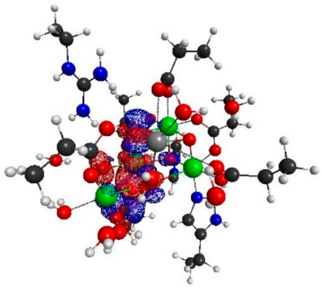
O(31)

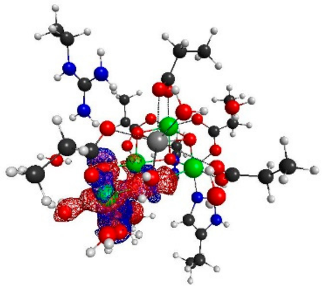
O(32)

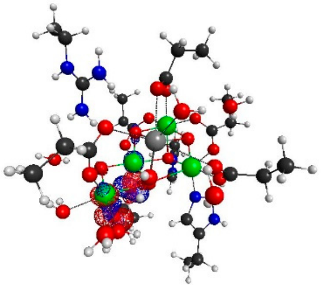
C(33)

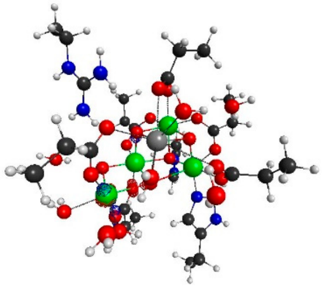
C(34)

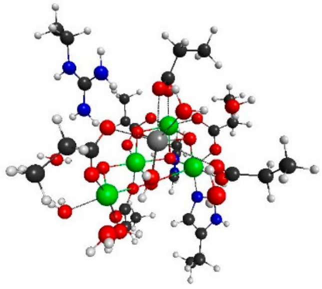
H(36)

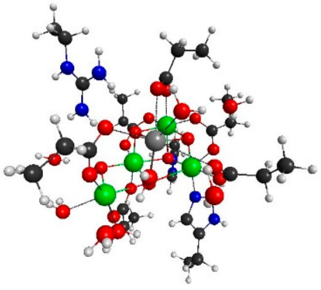
C(37)

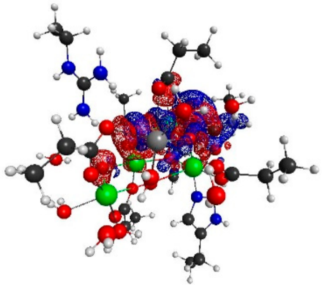
O(41)

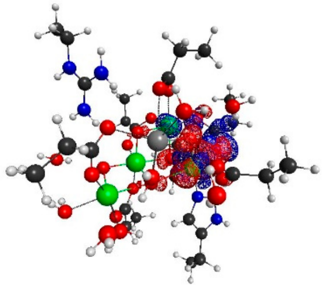
O(42)

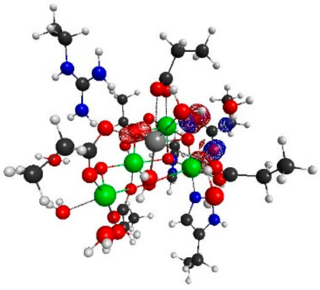

C(43)

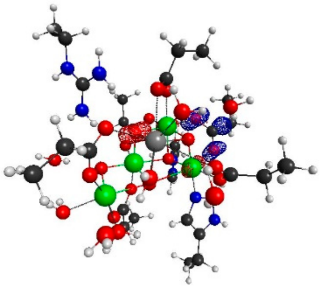

C(44)

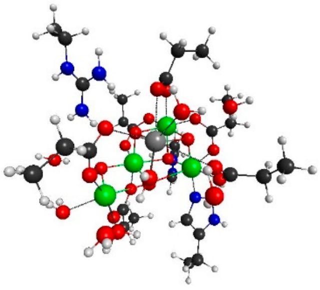

H(45)

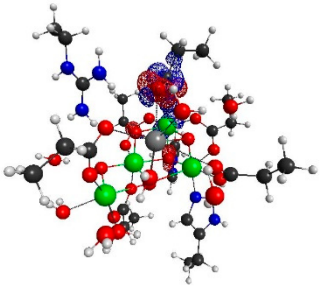

O(51)

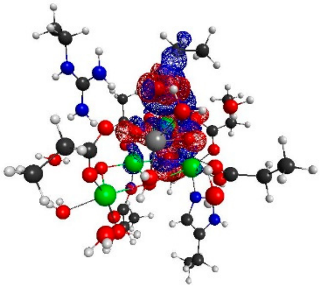

O(52)

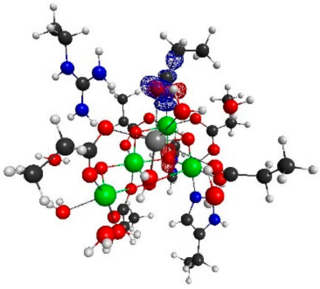

C(53)

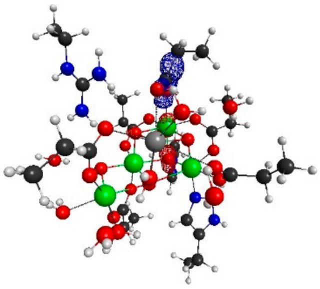

C(54)

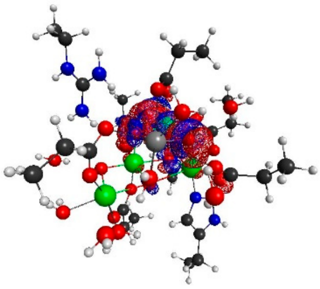

O(61)

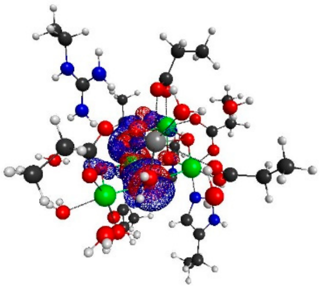

O(62)

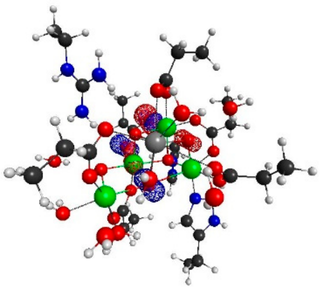

C(63)

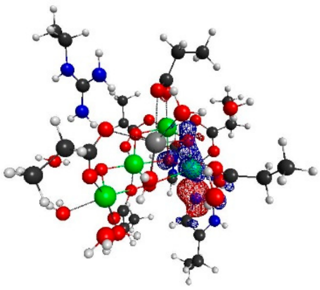

N(71)

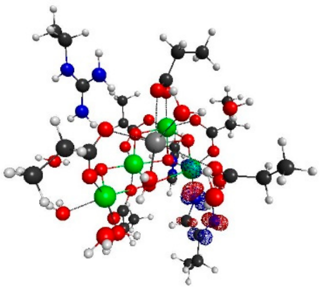

C(72)

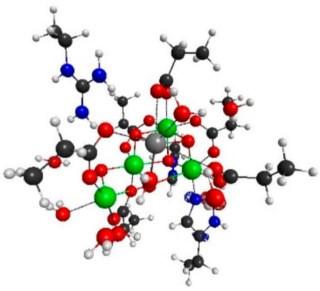

N(73)

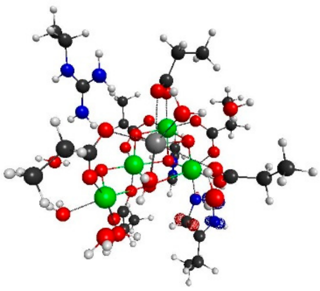

C(74)

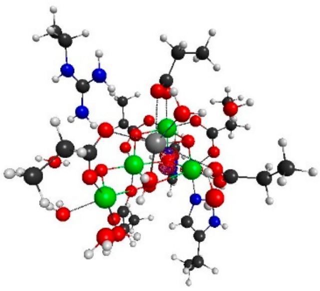

N(87)

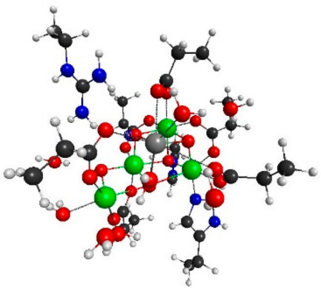

N(103)

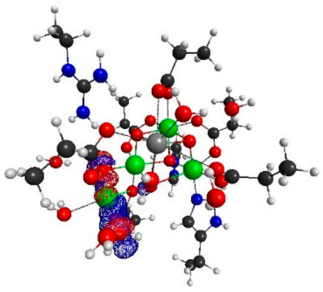

O(124)

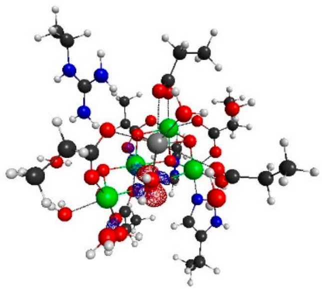

H(125)

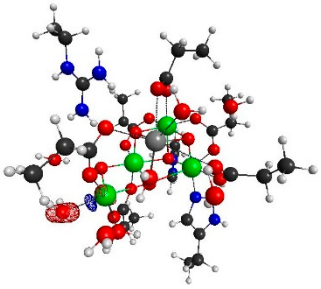

H(137)

Figure S-14. Isosurfaces of linear response functions of density for the perturbations on atomic sites of OEC (pdb entry: 5b66). The threshold of $\delta\rho_z(\boldsymbol{r})/\delta v_m(I)$ are ± 0.001 (+: blue, −: red). The atomic symbols and those numbers in parentheses below the figures indicate the atoms (I) to which the virtual perturbations are applied. The figures of $\delta\rho_z(\boldsymbol{r})/\delta v_m(I)$ that vanishes for this threshold are omitted.

Table S-3. The errors of the calculated values obtained by the QM cluster and the QM/MM models from the reference values on densities ($\Delta\rho$) of OEC. The reference values are those of the full model shown in figure 11.

	**Model 1**		**Model 2**		**Model 3**		**Model 4**	
	**QM**	**QM/MM**	**QM**	**QM/MM**	**QM**	**QM/MM**	**QM**	**QM/MM**
Mn(1)	0.941	1.069	0.051	0.032	0.041	0.025	0.004	0.005
Mn(2)	0.835	1.050	0.036	0.026	0.030	0.021	0.017	0.014
Mn(3)	0.571	0.687	0.041	0.013	0.024	-0.001	0.019	0.001
Mn(4)	1.071	1.134	0.237	0.365	0.026	0.043	0.009	0.024
O(5)	0.185	0.192	-0.003	0.024	-0.001	0.025	-0.013	0.005
O(6)	0.230	0.158	0.036	-0.006	0.042	-0.003	0.037	-0.014
O(7)	0.236	0.033	-0.015	-0.034	-0.019	-0.036	-0.008	-0.007
O(8)	0.380	0.178	0.071	0.021	0.021	-0.002	0.011	-0.016
O(9)	0.224	0.153	-0.004	0.002	-0.009	0.002	-0.011	0.004
Ca(10)	1.158	1.175	0.364	0.412	0.022	0.004	0.014	-0.005
O(11)			0.001	0.018	-0.018	0.004	-0.023	-0.005
O(12)			0.032	-0.017	0.023	-0.030	0.013	-0.037
C(13)			-0.009	0.007	-0.033	-0.014	-0.007	-0.001
C(14)							-0.043	-0.037
O(21)			-0.029	-0.015	-0.013	-0.013	-0.028	-0.035
O(22)			0.013	0.008	0.006	0.005	-0.003	-0.002
C(23)			-0.014	-0.008	-0.026	-0.012	-0.003	0.002
C(24)							-0.039	-0.038
O(31)			-0.002	-0.019	-0.010	-0.024	-0.006	-0.010
O(32)			0.029	0.034	0.011	0.019	-0.005	0.002
C(33)			0.000	-0.008	-0.019	-0.019	0.000	-0.001
C(34)							-0.041	-0.039
O(41)			-0.004	-0.009	-0.012	-0.013	-0.014	-0.011
O(42)			0.003	-0.012	-0.001	-0.014	-0.003	-0.003
C(43)			-0.021	-0.028	-0.026	-0.031	0.003	-0.001
C(44)							-0.040	-0.038
O(51)			0.005	-0.028	0.017	-0.007	0.002	-0.027
O(52)			0.013	0.022	0.006	0.017	-0.004	-0.004
C(53)			-0.007	0.005	-0.017	0.001	0.001	0.003
C(54)							-0.034	-0.032
O(61)			0.008	-0.007	0.003	-0.010	-0.005	-0.007
O(62)			0.017	0.001	0.012	-0.001	0.007	-0.005
C(63)			-0.016	-0.029	-0.021	-0.032	0.005	-0.002
C(64)							-0.035	-0.036
N(71)			-0.029	-0.044	-0.031	-0.045	0.008	0.004
C(72)							0.003	0.004
N(73)							-0.003	0.001
C(74)							-0.049	-0.046
C(75)							0.014	0.012
H(76)							0.001	0.003
H(77)							0.005	0.005
H(78)							0.001	0.005
H(86)			0.003	0.003	0.003	0.002	0.001	0.002
N(87)			0.014	0.005	0.012	0.004	0.009	0.010
C(88)							0.006	0.013
N(89)							0.000	0.003
C(90)							-0.040	-0.048
C(91)							0.019	0.019
H(92)							0.011	0.014

H(93)			0.008	0.009
H(94)			0.002	0.006
O(118)	0.009	0.010	0.005	0.007
H(119)	0.009	0.013	0.005	0.007
H(120)	0.026	0.031	0.023	0.029
O(121)	-0.007	0.010	-0.009	0.006
H(122)	0.001	-0.001	-0.002	0.000
H(123)	0.006	0.006	0.001	0.001
O(124)	0.001	-0.003	0.000	-0.004
H(125)	0.006	0.005	0.003	0.000
H(126)	0.011	0.024	0.009	0.021
O(127)	0.008	-0.003	0.010	-0.001
H(128)	0.015	0.036	0.015	0.037
H(129)	0.017	0.028	0.010	0.019

Table S-4. The errors of the calculated values obtained by the QM cluster and the QM/MM models from the reference values on densities ($\Delta\rho_z$) of OEC. The reference values are those of the full model shown in figure 11.

	Model 1		Model 2		Model 3		Model 4	
	QM	QM/MM	QM	QM/MM	QM	QM/MM	QM	QM/MM
Mn(1)	0.321	0.305	-0.012	-0.015	-0.012	-0.015	-0.003	-0.002
Mn(2)	-0.858	-0.634	-0.012	-0.026	-0.026	-0.033	-0.020	-0.004
Mn(3)	0.267	0.272	-0.010	0.027	-0.027	0.015	-0.033	0.008
Mn(4)	-0.630	-0.507	-0.162	-0.141	-0.007	-0.001	-0.006	0.000
O(5)	0.112	0.148	-0.005	0.004	-0.002	0.005	0.000	0.002
O(6)	0.224	0.157	0.012	0.003	0.033	0.014	0.031	0.004
O(7)	0.167	0.116	-0.017	-0.015	-0.008	-0.011	-0.004	-0.001
O(8)	0.627	0.306	0.092	0.036	0.019	-0.006	0.021	0.001
O(9)	-0.111	-0.048	-0.040	-0.060	0.021	-0.016	0.026	-0.011
Ca(10)	-0.003	-0.001	0.002	0.000	0.001	0.000	0.001	0.000
O(11)			0.016	0.027	-0.001	0.004	-0.001	0.000
O(12)			-0.002	0.000	-0.003	-0.001	-0.002	0.000
C(13)			0.003	0.004	0.001	0.001	0.001	0.000
C(14)							0.000	0.000
O(21)			0.003	0.006	0.003	0.006	0.001	0.001
O(22)			-0.001	0.004	-0.002	0.003	-0.001	0.001
C(23)			0.000	-0.002	-0.001	-0.002	0.000	0.000
C(24)							0.000	0.000
O(31)			0.000	-0.001	0.000	-0.001	0.000	0.000
O(32)			0.017	0.016	0.000	0.002	-0.002	0.000
C(33)			0.001	-0.001	-0.001	-0.002	0.000	0.000
C(34)							0.000	0.000
O(41)			-0.002	0.000	-0.002	0.000	-0.002	-0.001
O(42)			0.019	0.033	0.014	0.030	0.007	0.005
C(43)			0.003	0.004	0.002	0.004	0.001	0.000
C(44)							0.001	0.000
O(51)			-0.002	0.000	-0.002	0.000	-0.002	-0.001
O(52)			0.019	0.033	0.014	0.030	0.007	0.005
C(53)			0.003	0.004	0.002	0.004	0.001	0.000
C(54)							0.001	0.000
O(61)			0.004	-0.003	0.002	-0.003	0.003	-0.001
O(62)			-0.001	0.005	-0.001	0.004	-0.003	0.002
C(63)			0.001	0.001	0.001	0.001	0.000	0.000
C(64)							0.000	0.000
N(71)			0.014	0.015	0.015	0.015	0.002	0.001
C(72)							-0.001	-0.001
N(73)							-0.001	0.000
C(74)							0.000	0.000
C(75)							0.001	0.001
H(76)							0.000	0.000
H(77)							0.000	0.000
H(78)							0.000	0.000
H(86)			0.000	0.000	0.000	0.000	0.000	0.000
N(87)			0.000	0.000	0.000	0.001	0.000	0.000
C(88)							0.000	0.000
N(89)							0.000	0.000
C(90)							0.000	0.000
C(91)							0.000	0.000
H(92)							0.000	0.000

H(93)			0.000	0.000
H(94)			0.000	0.000
O(118)	0.001	-0.003	0.002	0.000
H(119)	0.000	0.000	0.000	0.000
H(120)	0.000	0.000	0.000	0.000
O(121)	-0.001	0.001	-0.002	0.000
H(122)	0.000	0.000	0.000	0.000
H(123)	0.000	0.000	0.000	0.000
O(124)	0.000	0.000	0.000	0.000
H(125)	0.000	0.000	0.000	0.000
H(126)	0.000	0.000	0.000	0.000
O(127)	0.000	0.000	0.000	0.000
H(128)	0.000	0.000	0.000	0.000
H(129)	0.000	0.000	0.000	0.000
